# Widespread use of invalid statistical tests in biomedical machine learning

**DOI:** 10.64898/2026.05.17.724301

**Published:** 2026-05-22

**Authors:** Tianchu Zeng, Hetu Li, Shaoshi Zhang, Yan Quan Tan, Fang Tian, Csaba Orban, Lijun An, Wanyu Che, Jingwen Cheng, Joanna Su Xian Chong, Niousha Dehestani, Zijian Dong, Xin Li, Zhizhou Li, Mervyn Jun Rui Lim, Yi Lin, Qinrui Ling, Zijie Ling, Xi Zhi Low, Sina Mansour L., Eric Kwun Kei Ng, Thuan Tinh Nguyen, Leon Qi Rong Ooi, Shreya Pande, Xing Qian, Jingxuan Ruan, Ziwen Wang, Yapei Xie, Chen Zhang, Yichi Zhang, Kaustubh Patil, Linden Parkes, Elvisha Dhamala, Sidhant Chopra, Andrew Zalesky, Avram Holmes, Simon Eickhoff, Juan Helen Zhou, Olivier Renaud, Nico Dosenbach, Konrad Kording, Danilo Bzdok, Thomas E. Nichols, B.T. Thomas Yeo

**Affiliations:** 1 Centre for Sleep & Cognition & Centre for Translational Magnetic Resonance Research, Yong Loo Lin School of Medicine, National University of Singapore, Singapore; 2 Department of Electrical and Computer Engineering, National University of Singapore, Singapore; 3 N.1 Institute for Health, National University of Singapore, Singapore; 4 Department of Medicine, Yong Loo Lin School of Medicine, National University of Singapore, Singapore; 5 Healthy Longevity Translational Research Programme, Yong Loo Lin School of Medicine, National University of Singapore, Singapore; 6 Human Potential Translational Research Programme, Yong Loo Lin School of Medicine, National University of Singapore, Singapore; 7 Institute for Digital Medicine (WisDM), Yong Loo Lin School of Medicine, National University of Singapore, Singapore; 8 Integrative Sciences and Engineering Programme (ISEP), National University of Singapore, Singapore; 9 Department of Clinical Sciences Malmö, SciLifeLab, Lund University, Lund, Sweden; 10 Division of Neurosurgery, Department of Surgery, National University Hospital, Singapore, Singapore; 11 Department of Electronic Engineering and Information Science, University of Science and Technology of China, Hefei, China; 12 Systems Neuroscience Lab, Department of Psychiatry, The University of Melbourne, Parkville, Victoria, Australia; 13 Institute of Neuroscience and Medicine, Brain & Behaviour (INM-7), Research Center Jülich, Jülich, Germany; 14 Institute for Systems Neuroscience, Medical Faculty, Heinrich-Heine University Düsseldorf, Düsseldorf, Germany; 15 Department of Psychiatry, Brain Health Institute, Rutgers University, Piscataway, NJ, USA; 16 Institute of Behavioral Sciences, Feinstein Institutes for Medical Research, Manhasset, USA; 17 Department of Psychiatry, Donald and Barbara Zucker School of Medicine at Hofstra/Northwell, Uniondale, Hempstead, USA; 18 Orygen, The National Centre of Excellence in Youth Mental Health, Melbourne, Victoria, Australia; 19 Center for Youth Mental Health, University of Melbourne, Melbourne, Victoria, Australia; School of Medicine, St Louis, MO, USA; 20 Department of Biomedical Engineering, The University of Melbourne, Parkville, Victoria, Australia; 21 Department of Psychiatry, Rutgers University, Piscataway, NJ, USA; 22 Center for Advanced Human Brain Imaging Research, Rutgers University, Piscataway, NJ, USA; 23 Department of Psychology, University of Geneva, Genève, Switzerland; 24 Mallinckrodt Institute of Radiology, Washington University School of Medicine, St Louis, MO, USA; 25 Allied Labs for Imaging Guided Neurotherapies (ALIGN), Washington University School of Medicine, St Louis, MO, USA; 26 Department of Neurology, Washington University School of Medicine, St Louis, MO, USA; 27 Department of Paediatrics, Washington University School of Medicine, St Louis, MO, USA; 28 Department of Biomedical Engineering, Washington University, St Louis, MO, USA; 29 Department of Psychological and Brain Sciences, Washington University, St Louis, MO, USA; 30 CIFAR Learning in Machines and Brains program, Toronto, Ontario, Canada; 31 Departments of Bioengineering and Neuroscience, University of Pennsylvania, Philadelphia, PA, USA; 32 The Neuro, McConnell Brain Imaging Centre, Department of Biomedical Engineering, Montreal, Quebec, Canada; 33 Faculty of Medicine, School of Computer Science, McGill University, Montreal, Quebec, Canada; 34 Mila–Quebec Artificial Intelligence Institute, Montreal, Quebec, Canada; 35 Big Data Institute, Li Ka Shing Centre for Health Information and Discovery, Nuffield Department of Population Health, University of Oxford, Oxford, UK; 36 Centre for Integrative Neuroimaging (OxCIN), FMRIB, Nuffield Department of Clinical Neurosciences, University of Oxford, Oxford, UK; 37 Athinoula A. Martinos Center for Biomedical Imaging, Massachusetts General Hospital, Charlestown, USA

## Abstract

Machine learning is accelerating biomedical research. Cross-validation is widely used to compare predictive performance – not only to benchmark algorithms, but also to inform scientific applications, such as ranking biomarkers. However, prediction performance estimates across cross-validation folds are not independent. Standard tests for comparing prediction performance (e.g., paired t-test) assume independence and can therefore inflate false positive rates. In a PRISMA-guided meta-analysis of 210 studies (impact factor ≥15, 1 June 2020 – 1 June 2025), we find that 97% ignored fold dependence when comparing prediction performance. This problem is ubiquitous across scientific fields and unaffected by impact factor, rigor-promoting policies, or open science practices. Simulations across 420 scenarios spanning four diverse datasets show that ignoring fold dependence leads to invalid false positive control in most settings. Repeated cross-validation further compounds this problem, with false positive rates rising toward 100% as the number of repetitions grows. Existing fold-dependence-aware tests rely on strong assumptions because the variance of fold-level statistics and the between-fold correlation cannot be disentangled under standard cross-validation. We therefore propose the SHARP (Split-HAlf RePeated) test, a simple modification to standard cross-validation that enables direct estimation of variance and correlation. Benchmarked against 12 tests, SHARP provides the best overall balance of false-positive control, statistical power, and confidence-interval calibration across simulation schemes. We conclude by providing best practices and reporting guidelines for valid model comparison inference in biomedical machine learning and beyond.

## 1 Introduction

**Figure 1.**
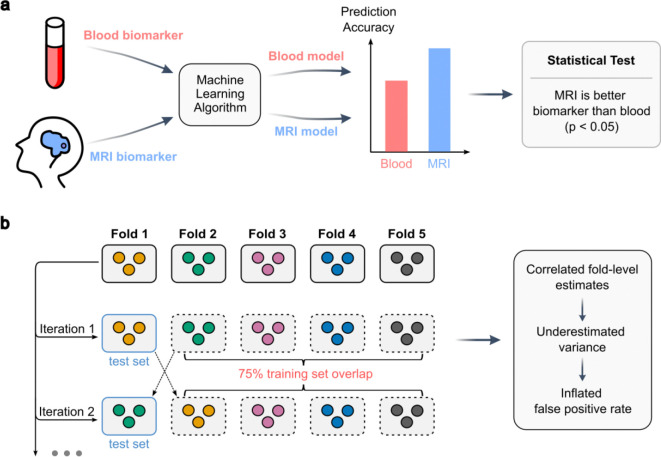
Comparing the predictive power of biomarkers using machine learning and statistical dependence induced by cross-validation. **a.** Machine learning model comparison can inform scientific and clinical applications. In this hypothetical example, the same algorithm is trained to predict dementia progression from blood or MRI biomarkers, and a statistical test compares their performance on held-out data. Because the algorithm is held constant, superior performance of the MRI model would suggest that MRI is more informative than blood. **b.** K-fold cross-validation (CV), illustrated for K=5. The dataset is partitioned into five folds. In each iteration, one fold serves as the test set (blue outline) and the remaining four folds form the training set (dashed outline). Training sets overlap substantially across iterations, and each test fold contributes to the training set in all other iterations. This induces positive correlations across fold-level estimates. Treating the fold-level estimates as independent underestimates the true variance, which inflates false positive rates (Nadeau & Bengio, 2003). Methods Section 4.1 discusses the properties of other variants of standard cross-validation.

Biomedical research increasingly relies on machine learning to extract predictive information from complex, high-dimensional data (Rajpurkar et al., 2022; Moor et al., 2023; Bzdok et al., 2024; Perez-Lopez et al., 2024). As algorithms and data modalities proliferate, empirical comparisons of predictive performance have become routine, guiding decisions about which algorithms to deploy or which biomarkers to prioritize (Greene et al., 2018; Wagner et al., 2023; Carrasco-Zanini et al., 2024; Yoo et al., 2025). Consider a hypothetical study in which the same algorithm is trained to predict dementia progression from either blood or MRI biomarkers (Fig. 1a). Because the algorithm is held constant, superior performance of the MRI-based model would indicate that MRI carries more information about dementia progression than blood. Here, the goal extends beyond benchmarking algorithms to informing scientific and clinical applications.

A standard approach for comparing predictive performance is K-fold cross-validation. The dataset is partitioned into *K* non-overlapping subsets (“folds”) of observations, such as brain scans or blood samples. In each iteration, one fold serves as the test set while the remaining *K* − 1 folds form the training set (Fig. 1b). When applied to two competing biomarkers (or algorithms), this procedure yields *K* pairs of prediction performance estimates that can be compared using a statistical test. Although test folds do not overlap across iterations, training sets overlap substantially, and each test fold contributes to the training set in all other iterations (Fig. 1b). The prediction performance estimates are therefore statistically dependent across folds (Dietterich, 1998; Bates et al., 2024). Conventional statistical tests (e.g., paired t-tests or Wilcoxon signed-rank tests) assume independence, so they underestimate variability and inflate false positive rates (Fig. 1b; Nadeau & Bengio, 2003; Jafrasteh et al., 2025). Repeated cross-validation, often recommended to improve stability (Bouckaert & Frank, 2004; Varoquaux et al., 2017), introduces further overlap across training and test sets, which can worsen the problem (Nadeau & Bengio, 2003).

Although fold-dependence has been recognized for decades (Dietterich, 1998; Nadeau & Bengio, 2003), its prevalence and consequences remain unclear. We ourselves have inadvertently applied statistical tests that ignore fold dependence (Yeo et al., 2010; Mansour L. et al., 2021; Chopra et al., 2024), raising a broader question: how often is fold dependence ignored in biomedical research, and does this vary across scientific fields? Furthermore, if existing publishing safeguards – editorial selectivity, statistical review, methodological reporting standards, and the scrutiny enabled by open data and code – were catching invalid tests, prevalence should be lower in studies meeting these criteria.

Beyond prevalence, fully addressing the fold-dependence problem requires confronting a fundamental statistical ambiguity. A single run of standard K-fold cross-validation yields K fold-level estimates of prediction performance differences, and three unknowns: true mean, true variance, and true between-fold correlation. The K fold-level differences, however, provide only two sources of information – their sample mean and sample variance. The sample variance underestimates the true variance, and is on average equal to the true variance × (1 – true correlation). Thus the true variance and correlation cannot be disentangled without additional assumptions (Nadeau & Bengio, 2003; Bengio & Grandvalet, 2004). Existing tests cope by making strong (implicit or explicit) assumptions about the true variance or correlation rather than estimating both quantities from data. When those assumptions fail, the result can be false positive inflation or lower statistical power.

Here we perform a PRISMA-guided meta-analysis of 210 PubMed-listed studies (impact factor ≥ 15), quantifying how often fold dependence is ignored, and evaluating whether prevalence varies by scientific field, impact factor, journal policy, or open science practice. We then develop and apply a battery of 420 simulation scenarios spanning image recognition, neuroimaging, ecology, and systems biology. Invalid statistical tests fail to control false positives in most settings, with false positive rates rising toward 100% under repeated cross-validation. We propose the SHARP (Split-HAlf RePeated) test, which modifies standard cross-validation to generate pairs of independent statistics, enabling direct estimation of variance and correlation. Benchmarking against 12 existing tests, SHARP reliably controls false positives and yields well-calibrated confidence intervals, while matching or exceeding the statistical power of valid tests. Finally, our meta-analysis also reveals that over half the studies comparing model performance either did not apply a statistical test or report confidence intervals, or described their procedure too vaguely to evaluate, so we also provide a concrete set of reporting recommendations for predictive model comparison.

## 2 Results

### 2.1 Invalid statistical tests in 97% of studies

**Figure 2.**
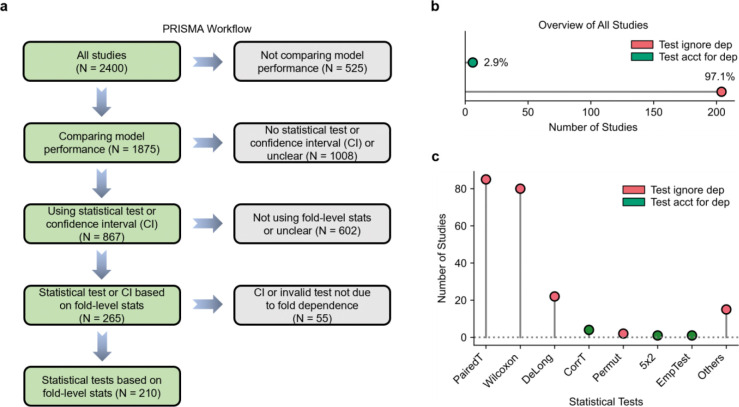
Widespread use of invalid statistical tests in biomedical studies using cross-validation to compare predictive performance. **a.** PRISMA flow diagram of the meta-analysis. A PubMed search (1 June 2020–1 June 2025; journals with impact factor ≥15) identified 2,400 records, of which 1,875 compared model performance. Among these, 867 clearly applied statistical tests or confidence intervals. We further excluded 602 studies without fold-level statistics or with unclear procedures, one study with a permutation test invalid for reasons unrelated to fold dependence, and 54 studies reporting only confidence intervals, leaving 210 studies for analysis. **b.** Breakdown of the 210 studies by statistical approach: tests assuming fold independence (red) versus tests accounting for fold dependence (green). **c.** Breakdown of specific statistical tests across the 210 studies. Red indicates tests that assume fold independence, i.e., invalid tests. Green indicates tests that account for fold dependence. “PairedT” refers to the uncorrected resampled paired t-test (Nadeau & Bengio, 2003); “Wilcoxon” refers to either the Wilcoxon signed-rank test (Wilcoxon, 1945) or the Wilcoxon rank-sum test (Mann & Whitney, 1947); “DeLong” refers to DeLong’s test (DeLong et al., 1988); “Permut” refers to paired permutation test (Edgington & Onghena, 2007); “CorrT” refers to the corrected resampled paired t-test (Nadeau & Bengio, 2003); “5×2” refers to the “5×2 paired t-test” (Dietterich, 1998); and “EmpTest” refers to a variant of the empirical test of differences (Parkes et al., 2021a).

To assess how often invalid statistical tests are used, we conducted a meta-analysis following the Preferred Reporting Items for Systematic Reviews and Meta-Analyses (PRISMA) guidelines (Page et al., 2021; Fig. 2a; Methods Section 4.2). Using PubMed, we identified original research articles published between 1 June 2020 and 1 June 2025 that used cross-validation to compare predictive performance. We restricted inclusion to journals with impact factor ≥ 15, which typically have the most stringent reporting standards, statistical review, and transparency policies. This is a conservative criterion: if the problem is prevalent even in venues with the strongest methodological safeguards, it is likely to be as prevalent elsewhere.

The initial search yielded 2,400 studies, of which 1,875 compared model performance (Fig. 2a). Of these, 1,008 studies (54%) did not clearly apply a statistical test or report confidence intervals when comparing model performance. In some cases, models were compared based on point estimates alone; in others, reporting was too vague to determine whether any statistical test was performed. We focused on the remaining 867 studies that clearly applied a statistical test or reported confidence intervals (Fig. 2a). Of these, 602 were excluded because the actual statistical procedure was unclear or did not use fold-level statistics (Methods Section 4.2.2), resulting in 265 studies (Fig. 2a).

We excluded one study using a permutation test that was invalid for reasons unrelated to fold dependence (see Supplementary Results). We also excluded studies reporting only confidence intervals, because the method of computation was rarely reported with sufficient clarity to assess validity (see Methods Section 4.2). This resulted in a final set of 210 studies (Fig. 2a).

The winnowing from 1,875 to 210 reflects broader reporting deficiencies in the ML literature — from studies where it was unclear whether any statistical test was performed, to studies with insufficiently described statistical procedures. These deficiencies are outside the scope of our fold-dependence analysis but warrant attention in their own right (see Discussion Section 3.4). Our meta-analysis focuses specifically on the 210 studies with sufficient clarity to assess fold dependence.

Of the 210 studies (Fig. 2b), 204 (97%) used statistical tests that assumed independent fold-level estimates. The paired t-test and Wilcoxon signed-rank test were the most common invalid tests (Fig. 2c). Further breakdown of meta-analysis results is found in Supplementary Results and supporting quotations from each study are provided in Supplementary File 1. As we show below (Section 2.4), these tests inflate FPR to 19% on average – nearly 4× the nominal 5% level – under a single round of 10-fold cross-validation, and break down entirely (FPR → 100%) under repeated cross-validation, a commonly recommended practice.

### 2.2 Widespread invalid statistical tests over time and scientific fields

We next examined whether the prevalence of invalid fold-based statistical tests (among the 210 studies) showed any discernible pattern over time or across scientific fields. Here, scientific fields are operationalized as Web of Science Journal Citation Reports subject categories (Methods Section 4.2.3).

**Figure 3.**
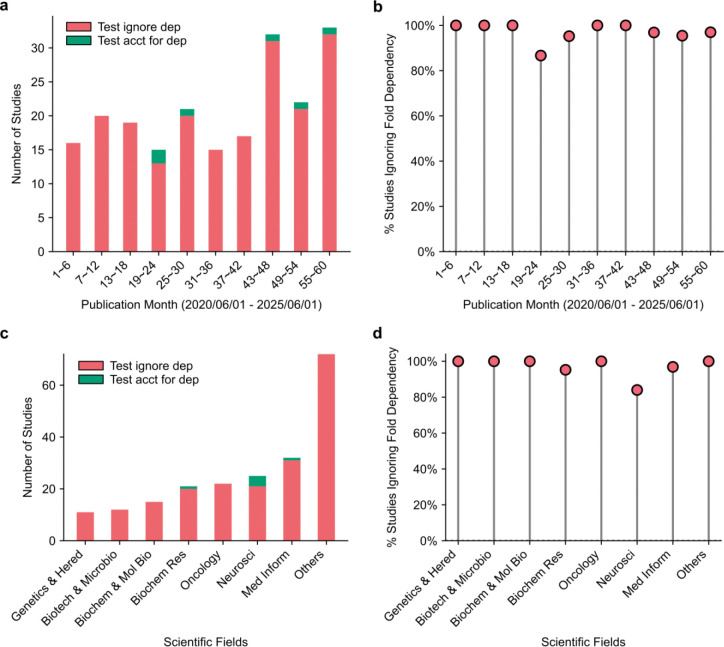
Persistent risk of inflated false positives from fold dependence over time and across scientific fields. **a.** Number of studies by publication period, in 6-month intervals from 1 June 2020 to 1 June 2025 (N = 210). **b.** Proportion of studies ignoring fold dependence over time. The proportion showed no detectable change (permutation test, p = 0.63). **c.** Number of studies by scientific field. The 210 studies spanned 33 fields. Fields with at least 10 studies are shown separately, while the remaining 26 fields are grouped as “Others”. **d.** Proportion of studies ignoring fold dependence by scientific field. The proportion of studies ignoring fold dependence did not differ significantly across scientific fields (permutation test, p=0.07). Note that the “Others” category was excluded from the statistical comparison across fields. Details about statistical tests are found in Methods Section 4.2.7.

The number of studies grew by 14.4% per year from 1 June 2020 to 1 June 2025 (Poisson regression p=6e-3; Fig. 3a), reflecting the increasing use of cross-validation to compare machine learning models. However, the proportion of studies ignoring fold dependence showed no detectable change over time, remaining persistently high across all periods (permutation test p=0.63; Fig. 3b).

Across scientific fields, reliance on invalid fold-independence assumptions was ubiquitous (Figs. 3c,d). Among fields with at least 10 studies, 100% of studies in “Genetics & Heredity”, “Biotechnology & Applied Microbiology”, and “Biochemistry & Molecular Biology” ignored fold dependence (Fig. 3d). Neuroscience had the lowest proportion, at 84.0%. Nevertheless, the proportion did not differ significantly across fields (permutation test p=0.07). Together, these results show that the use of invalid tests has neither declined over time nor varied meaningfully across scientific fields.

### 2.3 Invalid tests used regardless of impact factor, policy or open science practices

We next examined whether the use of invalid statistical tests was associated with journal impact factor, journal policies promoting scientific rigor, or open science practices. If existing publishing safeguards were filtering out invalid tests, we would expect lower prevalence in journals or studies satisfying these criteria — through editorial selectivity, explicit methodological policies, or the post-hoc scrutiny enabled by shared data and code.

**Figure 4.**
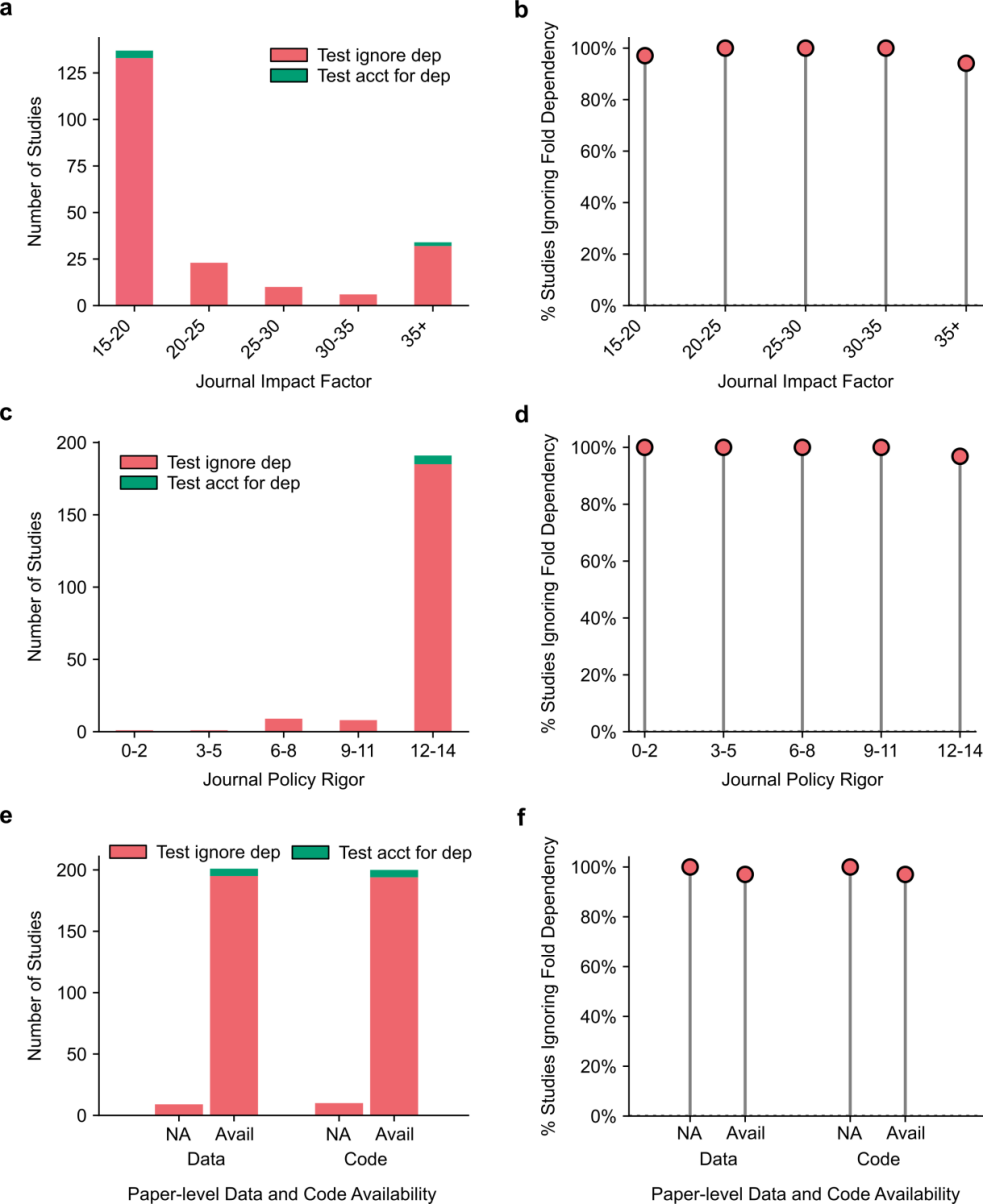
Neglect of fold-dependence persists across impact factor, journal policies for scientific rigor and open science practices. **a.** Distribution of studies by journal impact factor. **b.** Proportion of studies ignoring fold dependence by impact factor bins, calculated as the number of studies using statistical tests ignoring fold dependence divided by the total number of studies in each bin. No trend was detected (permutation test p=0.59). **c.** Distribution of studies by journal policy rigor score, ranging from 0 (least rigorous) to 14 (most rigorous). **d.** Proportion of studies ignoring fold dependence by journal policy rigor score. Increasing rigor did not reduce the use of invalid statistical tests (permutation test p=0.86). **e.** Number of studies ignoring fold dependence stratified by data and code availability. Most studies provided data, code or both. **f.** Proportion of studies ignoring fold dependence stratified by data and code availability. The proportion was not significantly associated with data or code availability (permutation test p=1.00 for both).

First, we stratified studies by the impact factor of the publishing journal (Fig. 4a). The proportion of studies ignoring fold dependence did not vary across impact factor bins (permutation test p=0.59; Fig. 4b).

For each journal, we assigned a rigor score (0 to 14) based on policies for transparency and reproducibility (e.g., code and data availability) and policies for statistical and methodological guidance (e.g., reporting checklists). Most journals had relatively stringent policies (Fig. 4c). Nevertheless, the proportion of studies ignoring fold dependence remained uniformly high regardless of journal rigor score (permutation test p=0.86; Fig. 4d).

Finally, most studies provided data, code, or both (Fig. 4e). Among studies that did not provide data, code, or both (N=16), 100% ignored fold dependence (Fig. 4f). However, the proportion of studies ignoring fold dependence was not associated with data availability (permutation test p=1.00) or code availability (permutation test p=1.00).

Together with Section 2.2, these results show that the prevalence of invalid tests is not explained by year, field, journal impact factor, journal rigor policies, or open science practices, pointing instead to a systemic gap in fold dependence awareness (see Discussion Section 3.3).

### 2.4 Ignoring fold dependence leads to poor control of false positive rate (FPR)

To assess the impact of invalid tests on false positive rates (FPR), we analyzed four datasets spanning image recognition (EMNIST Digits; Cohen et al., 2017), neuroimaging (UK Biobank; Alfaro-Almagro et al., 2018), ecological classification (Covertype; Blackard, 1998), and systems biology (KEGG Metabolic Pathway; Muhammad Naeem, 2011). Further details about the datasets are provided in Methods Section 4.3.

**Figure 5.**
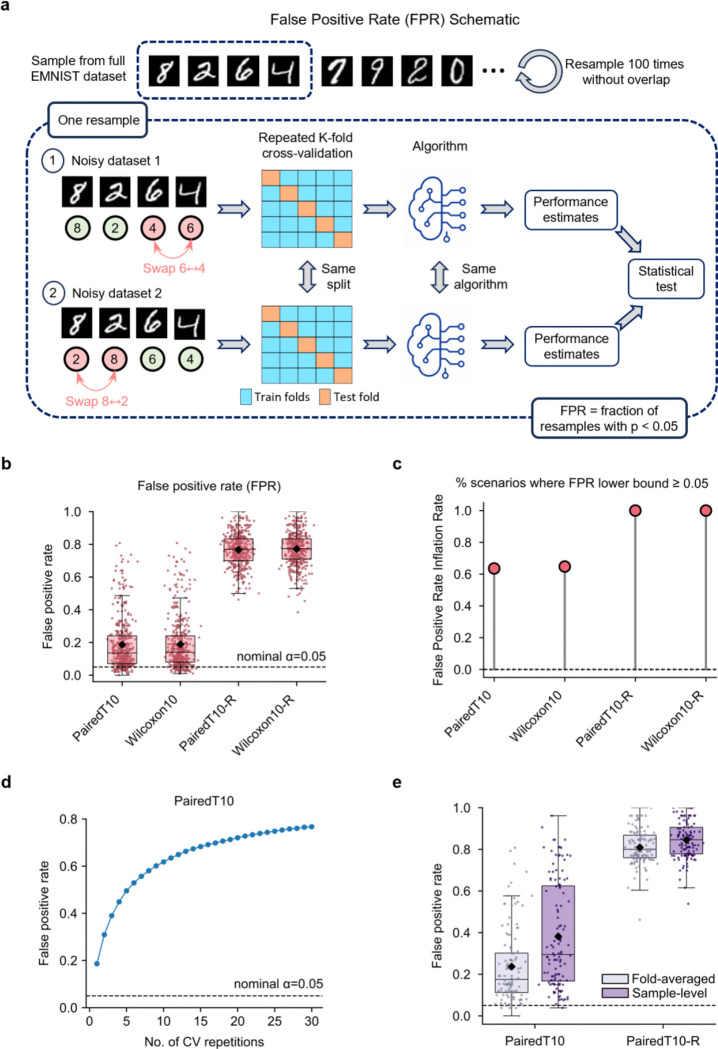
Ignoring fold dependence leads to poor control of false positive rates. **a.** False positive rate (FPR) simulation illustrated for the EMNIST dataset. We randomly sampled a dataset (e.g., N=1,000) from a full dataset, then generated two noisy versions by independently permuting the target labels (ground-truth digits) for a fixed percentage of samples (e.g., 10%). For example, noisy dataset 1 (top) has the labels for digits 6 and 4 swapped, while noisy dataset 2 (bottom) has the labels for digits 8 and 2 swapped, reflecting the independence of the two permutations. Using identical cross-validation splits and the same machine learning algorithm on each noisy dataset, we obtained pairs of cross-validated performance estimates — e.g., 300 pairs for 10-fold cross-validation repeated 30 times. A statistical test was applied to the paired vector to obtain a p-value. Because the two noisy datasets had the same noise level, the two trained models had identical expected predictive performance. At α = 0.05, a well-calibrated test should reject no more than 5% of the time. This procedure was repeated up to 100 times using non-overlapping subsamples. **b.** FPR across four datasets and four sample sizes. Each boxplot shows 420 datapoints, one per scenario. The black dot indicates the mean FPR. The dashed line indicates the nominal FPR (0.05). Abbreviations: PairedT10 / Wilcoxon10: paired t-test / Wilcoxon signed-rank test based on 10-fold cross-validation; PairedT10-R / Wilcoxon10-R: same tests with 10-fold cross-validation repeated 30 times. **c.** FPR inflation rate: percentage of the 420 scenarios in which the lower bound of the 95% confidence interval for FPR exceeded 0.05. **d.** FPR of the paired t-test as a function of the number of 10-fold cross-validation repetitions, averaged over 420 scenarios. The dashed line indicates the nominal FPR (0.05). Wilcoxon signed-rank test yields the same conclusions (not shown). **e.** FPR of the paired t-test using sample-level versus fold-averaged statistics, across 120 scenarios in the KEGG Metabolic Pathway dataset (one datapoint per scenario). The black dot indicates the mean FPR. The dashed line indicates the nominal FPR (0.05).

To estimate FPR, we constructed pairs of trained models with identical expected predictive performance and compared them using a statistical test (Fig. 5a). First, we randomly sampled a dataset of a given sample size (e.g., N=1,000) from one of the four datasets. Next, we generated two noisy versions of this dataset by independently permuting the target labels (e.g., digit labels for EMNIST) for the same fixed fraction of samples (e.g., 10%) in each copy. Finally, the same machine learning algorithm and cross-validation scheme were applied to both noisy copies. The independent noise ensured that the two trained models were distinct, while the matched noise level ensured equal expected performance under the null. Therefore, a well-calibrated test at significance threshold α = 0.05 should reject the null hypothesis no more than 5% of the time (Fig. 5a).

For each combination of dataset, sample size, and noise level — hereafter a “condition” — we repeated the above procedure on non-overlapping subsamples of the full dataset (typically 100 repetitions per condition; see Methods Section 4.4), yielding one p-value per repetition. The FPR for each condition was estimated as the fraction of repetitions in which the null was rejected. The independent repetition also enabled 95% confidence intervals for the FPR. For each dataset, we varied the noise level (10%, 20%, …, 100% labels permuted) and sample size (N = 100, 500, 1,000, 2,000; two datasets each excluded one sample size; see Methods Section 4.4), yielding (4+4+3+3) × 10 = 140 conditions. For each condition, we considered three hyperparameter-selection strategies: fixed hyperparameters, a single training-validation split, and nested cross-validation. This yielded 140 × 3 = 420 scenarios.

Across the 420 scenarios, the two most widely used tests (the paired t-test and Wilcoxon signed-rank test; Fig. 2c) exhibited an elevated FPR of ~19% when 10-fold cross-validation was performed once (Fig. 5b). Type I error was not controlled (lower bound of the 95% CI exceeded 0.05) in ~64% of scenarios, hereafter the “FPR inflation rate” (Fig. 5c). Moreover, when 10-fold cross-validation was repeated 30 times – as is often recommended for stability (Bouckaert & Frank, 2004; Varoquaux et al., 2017) – the FPR jumped to 77% (Fig. 5b) and the tests were invalid in 100% of scenarios. FPR rose monotonically with the number of repetitions (Fig. 5d), eventually reaching 100%. We emphasize that the problem lies not with repeated cross-validation itself, which has well-established benefits, but with its interaction with tests that assume fold independence.

The escalation of FPR with repeated cross-validation is not specific to our simulations: any test that assumes fold independence will behave this way when repeated cross-validation is applied. Even when two models have the same expected performance in the population (i.e., null hypothesis is true), a finite dataset will almost certainly show a small, non-zero sample-specific difference between the two models. Standard tests such as the paired t-test treat each fold-level observation as independent, effectively assuming the sample size grows with the number of folds and repetitions. Since any effect, however small, becomes significant when the sample size is large enough, the FPR is expected to approach 100% as the number of repetitions grows.

### 2.5 Sample-level statistics compound false positive rate inflation

Section 2.4 focused on tests using “fold-averaged” statistics. Our meta-analysis revealed that some studies instead used “sample-level” statistics, which may inflate FPR further. Consider a study comparing two models via 10-fold cross-validation on 1,000 samples. A fold-averaged approach averages performance within each fold, yielding 10 paired metrics for the statistical test. A sample-level approach forgoes this aggregation and enters all 1,000 paired metrics directly into the test. Both approaches derive values from test folds and were therefore included in our meta-analysis. Because statistics within the same fold might be even more strongly correlated than statistics across folds, sample-level statistics may inflate FPR further.

We first examined this issue using the KEGG Metabolic Pathway dataset. As expected, switching from fold-averaged to sample-level statistics (while still ignoring fold dependence) elevated FPR — from 24% to 38% for a single run of 10-fold cross-validation, and from 81% to 84% when repeated 30 times (Fig. 5e). We next examined DeLong's test, another popular sample-level test (Fig. 2c). Because DeLong's test applies only to binary classification (Methods Section 4.5.4), we evaluated it on the Covertype dataset, where it exhibited FPRs of 21% and 77% for 10-fold cross-validation with one repetition and 30 repetitions, respectively (Fig. S1). For comparison, the paired t-test on fold-averaged statistics in the same dataset yielded FPRs of 13% and 72% (Fig. S2c). Together, these results show that sample-level statistics compound the FPR inflation already caused by ignoring fold dependence.

### 2.6 SHARP avoids strong assumptions in existing fold-dependence-aware tests

Having shown that tests ignoring fold dependence severely inflate FPRs (Fig. 5), we next considered tests that account for it (hereafter “fold-aware” tests). Accounting for fold dependence must address a fundamental statistical ambiguity. In a single run of K-fold cross-validation, the K fold-level differences can be used to compute sample mean and sample variance, but the sampling distribution of the average difference involves three unknown parameters: true mean, true variance, and true between-fold correlation. Since expected sample variance = true variance × (1 – true correlation), the true variance and correlation cannot be jointly estimated without additional assumptions (Nadeau & Bengio, 2003). Repeated K-fold cross-validation inherits this non-identifiability, and no universal distribution-independent unbiased estimator of the standard error exists (Bengio & Grandvalet, 2004).

Existing tests address this non-identifiability issue in different ways, each with its own limitations. Among the six studies in our meta-analysis that used fold-aware tests (Fig. 2c): four used the corrected resampled t-test (Nadeau & Bengio, 2003), one used the 5×2 paired t-test (Dietterich, 1998), and one used a variant of the empirical test of differences (Parkes et al., 2021a). The corrected resampled t-test assumes that the between-fold correlation is equal to the fraction of samples held out in each test fold (Supplementary Methods S3). If true correlation is higher, FPR is inflated, and if lower, power is diminished. The 5×2 paired t-test uses 5 repetitions of 2-fold cross-validation to approximately correct for the between-fold correlation. However, it estimates the mean performance difference from a single fold, discarding information from the remaining nine folds at severe cost to power (Supplementary Methods S4). The empirical test of differences avoids modeling between-fold correlation by using the empirical distribution of fold-level differences. It is conservative when true correlation ≤ 0.5 and loses power when true correlation is much lower (Supplementary Methods S5). Overall, these assumptions risk inflating FPR or sacrificing power.

**Figure 6.**
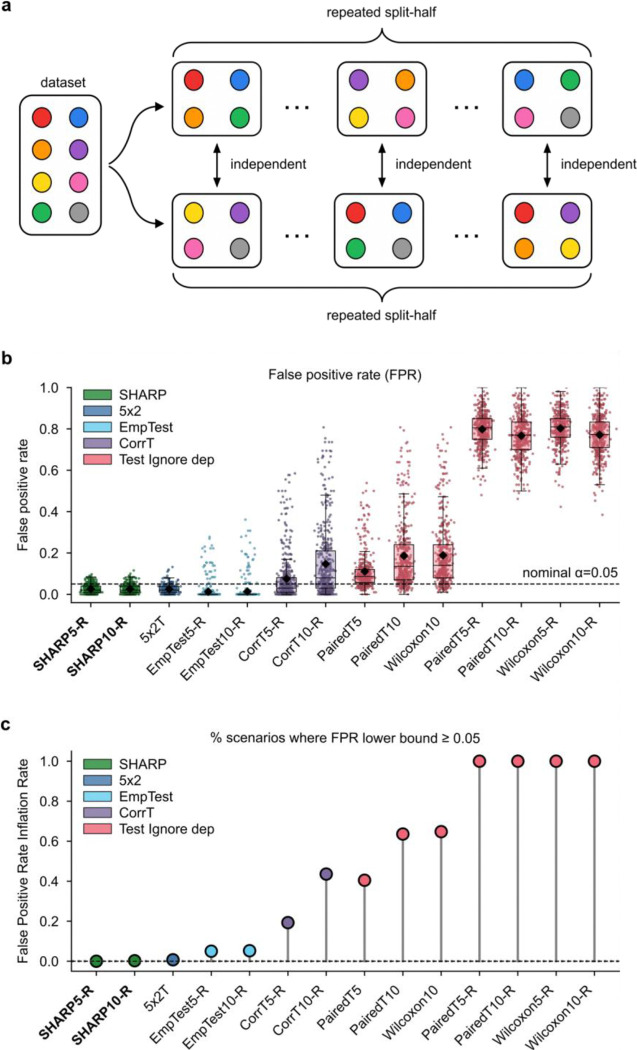
SHARP test and false positive rates (FPR) of statistical tests across 420 simulation scenarios. **a.** Schematic of the SHARP test (see Section 2.6 for motivation). The procedure performs J random splits of the dataset into disjoint halves (A and B) and runs K-fold cross-validation separately on each. Averaging the fold-level differences within a half produces a single statistic, giving two statistics per repetition. The disjoint sampling makes these two statistics independent within a repetition, even though statistics from different repetitions share data and are therefore correlated. This structure provides the two ingredients needed for a valid test: an estimate of each statistic's variance and an estimate of the across-repetition correlation. **b.** Boxplot of FPR for each statistical test, with one value per scenario (n = 420). Black dots mark the mean FPR across scenarios. The dashed horizontal line marks the nominal level of 0.05. **c.** FPR inflation rate: the percentage of the 420 scenarios in which the lower bound of the 95% confidence interval for FPR exceeded 0.05, indicating inadequate control of the Type I error rate. **Test naming conventions.** Numeric suffixes “5” and “10” denote 5-fold and 10-fold cross-validation. The “-R” suffix indicates repeated cross-validation: 5-fold repeated 60 times or 10-fold repeated 30 times, yielding 5 × 60 = 10 × 30 = 300 fold-level statistics. For example, “CorrT5-R” denotes the corrected resampled t-test evaluated under 60 repetitions of 5-fold cross-validation, while “PairedT10” denotes the naïve paired t-test under a single run of 10-fold cross-validation. “SHARP5-R” denotes the split-half procedure repeated 60 times with 5-fold cross-validation within each half; “SHARP10-R” denotes 30 repetitions with 10-fold cross-validation within each half. The number of repetitions was chosen such that the FPR of tests accounting for fold dependence had stabilized; tests that ignore fold dependence do not stabilize, with FPR increasing toward 1 as repetitions grow (Fig. 5d). “CorrT” denotes the corrected resampled t-test (Nadeau & Bengio, 2003). “EmpTest” denotes the empirical test of differences (Parkes et al., 2021a). For details of all tests, see Methods Sections 4.5 and 4.6.

We therefore propose the Split-HAlf RePeated (SHARP) test. In each of J repetitions, SHARP randomly partitions the dataset into two disjoint halves, A and B, and performs K-fold cross-validation independently within each half (Fig. 6a). Fold-level performance differences are averaged within each half, yielding one statistic per half. Because A and B share no observations, the two statistics from a given repetition are independent, while statistics from different repetitions remain correlated. This within-repetition independence is sufficient to estimate the variance of each split-half statistic and the across-repetition correlation, enabling a valid statistical test for assessing differences between two models (Methods Section 4.6, Supplementary Methods S7). We next evaluate whether this design delivers in practice, by comparing SHARP's false positive rate (Section 2.7), statistical power (Section 2.8), and confidence interval coverage (Section 2.9) against existing tests.

### 2.7 SHARP: reliable FPR control through direct variance estimation

Across 420 simulation scenarios (Section 2.4), the corrected resampled t-test, 5×2 paired t-test, and empirical test of differences markedly reduced FPR relative to tests ignoring fold dependence (Fig. 6b). The 5×2 paired t-test and empirical test of differences controlled FPR at the nominal 5% level. The corrected resampled t-test was borderline under 5-fold crossvalidation and failed under 10-fold, with FPR significantly exceeding 5% in 19% and 44% of scenarios, respectively (Fig. 6c). These conclusions held across datasets (Fig. S2) and sample sizes (Fig. S3). Because simulation results can depend on the underlying modeling assumptions, we repeated the analyses under an alternative scheme (Supplementary Methods S6) – results were unchanged, except that the corrected resampled t-test now controlled FPR (Fig. S4), indicating that its FPR control depends on the simulation scheme.

Across the same 420 scenarios, SHARP controlled FPR reliably (Fig. 6b), with CI lower bounds below 5% in nearly all scenarios (Fig. 6c), matching the 5×2 paired t-test and the empirical test of differences. Conclusions were consistent across datasets (Fig. S2), sample sizes (Fig. S3), and an alternative simulation scheme (Fig. S4). A broader comparison across additional tests (Methods Section 4.5, Supplementary Methods S8 & S9) confirmed the same pattern: tests that effectively ignored fold dependence — a misapplication of the corrected resampled t-test, the paired permutation test, and a bootstrap variant — had inflated FPR, while tests that accounted for it — the 5×2 paired F-test and two other bootstrap variants — controlled FPR reliably (Fig. S5).

### 2.8 SHARP matches or exceeds the power of existing valid tests

Aside from reliable FPR control, a statistical test should also have adequate power to detect genuine performance differences. As argued in Section 2.6, SHARP may have higher power than existing fold-aware tests. To test this prediction, we adapted the FPR simulation procedure from Section 2.4.

Instead of corrupting both copies of the sampled dataset with the same level of noise (Fig. 5a), we corrupted only one copy and left the other clean (Fig. S6). The model trained on the clean copy therefore had higher expected predictive performance by construction, and a sensitive test should reject the null hypothesis of equivalent performance. As with FPR, power was estimated within each of 420 scenarios as the fraction of repetitions in which the null was rejected, then averaged across scenarios. More details are in Methods Section 4.4.

**Figure 7.**
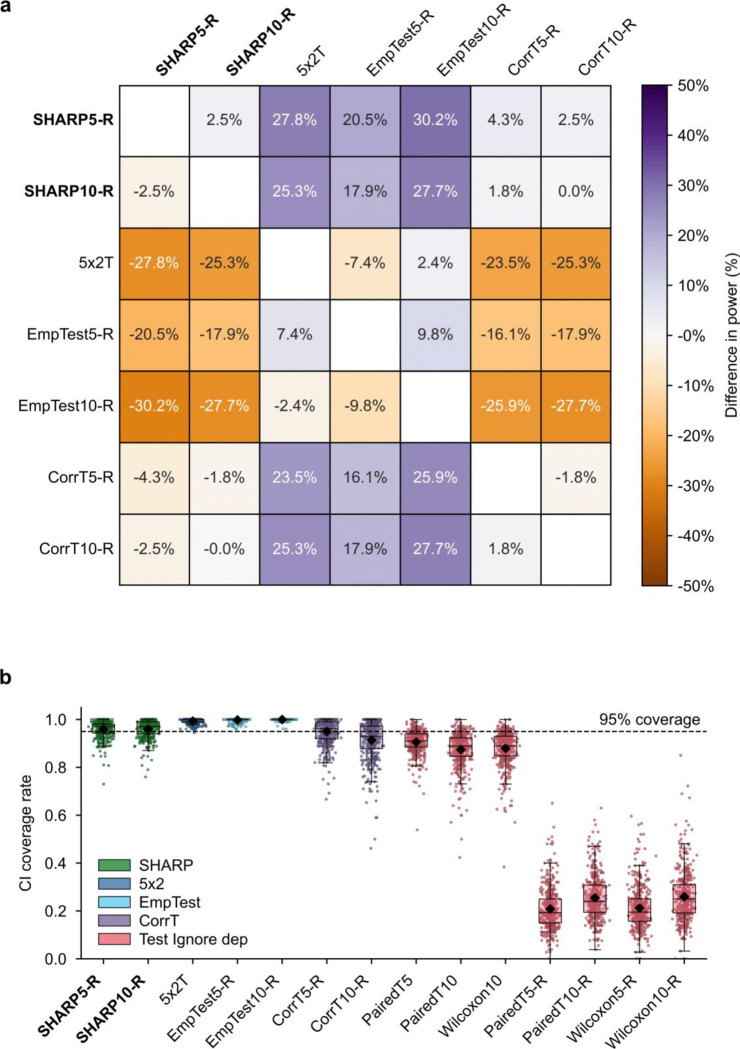
Comparison of statistical power and confidence interval coverage across 420 scenarios. **a**. Difference in power between pairs of statistical tests, computed as the power of the statistical test (on the row) minus the power of the statistical test (on the column), averaged across 420 scenarios. A purple cell indicates that the test on the row achieved higher power than the test on the column; an orange cell indicates the opposite. For example, the cell in the first row and third column is 27.8% and purple, indicating that SHARP5-R has higher power than the 5×2 paired t-test. The tests shown here are a subset of the tests in Fig. 6, restricted to only valid statistical tests. SHARP5-R won every pairwise comparison, as reflected in its entirely purple row. **b.** Comparison of 95% confidence interval (CI) coverage rate across 420 scenarios. The CI coverage rate of a scenario is defined as the percentage of non-overlapping subsamples in which the (estimated) true performance difference between models fell inside the 95% CI of a given test. For a well-calibrated test, the coverage rate should be exactly 95% (black dashed line). Each boxplot comprises 420 data points, each representing the CI coverage rate of one scenario. The black dot indicates the mean coverage rate across 420 scenarios. Tests that ignored fold dependence (red boxplots) had overly narrow CIs, so their coverage rates were much lower than 95%.

Fig. 7a shows the difference in average power between pairs of statistical tests. For example, the SHARP test based on 5-fold cross-validation (SHARP5-R) outperformed the 5×2 paired t-test by 27.8%, shown in the first row and third column of Fig. 7a. Overall, SHARP5-R had the highest power, reflected in its entirely purple row in Fig. 7a. A wider set of tests is shown in Fig. S7, where SHARP remained the most sensitive, followed by the corrected resampled t-test. As in Section 2.7, we cross-checked our findings under an alternative simulation scheme (Fig. S8; Supplementary Methods S6), which yielded the same conclusions, except that SHARP5-R and CorrT5-R achieved similar power.

Together with Section 2.7, these results show that SHARP achieves substantially higher power than the 5×2 paired t-test and the empirical test of differences, while matching them on FPR control. Compared with the corrected resampled t-test, SHARP had similar or slightly higher power, depending on the simulation scheme.

### 2.9 SHARP test demonstrates excellent confidence interval coverage

Beyond FPR and statistical power, good statistical practice requires more than a binary reject/fail-to-reject decision. A confidence interval communicates the magnitude and precision of the estimated effect, essential for interpreting results in context. A statistically significant difference may be too small to matter practically, while a non-significant result with a wide interval may suggest insufficient power rather than true equivalence.

For a well-calibrated test, the 95% confidence interval should contain the true performance difference between the two models 95% of the time (Methods Section 4.4.3). Across the 420 statistical power simulation scenarios, tests that ignored fold dependence produced overly narrow CIs, resulting in coverage well below 95% (Fig. 7b). The empirical test of differences and 5×2 paired t-test, which have low power (Fig. 7a), produced overly wide CIs with coverage approaching 100%.

The corrected resampled t-test achieved a mean coverage of 95% under 5-fold cross-validation but with wide variation across scenarios; this is consistent with its fixed between-fold correlation that cannot adapt to the data (Section 2.6). SHARP5-R (96%) and SHARP10R (96%) both performed well. More tests are shown in Fig. S9. These conclusions also held under an alternative simulation scheme (Fig. S10; Supplementary Methods S6), except that the corrected resampled t-test now achieved stable coverage – the same scheme-dependence noted for FPR in Section 2.7.

Across FPR, power, and confidence interval results, SHARP5-R best balances all three criteria among the tests examined. The corrected resampled t-test is a close second on power, but is less reliable on FPR control and CI coverage because it imposes assumptions about the magnitude of fold-level correlations – assumptions that SHARP's design avoids.

## 3 Discussion

### 3.1 Practical guidance for valid model comparison

**Figure 8.**
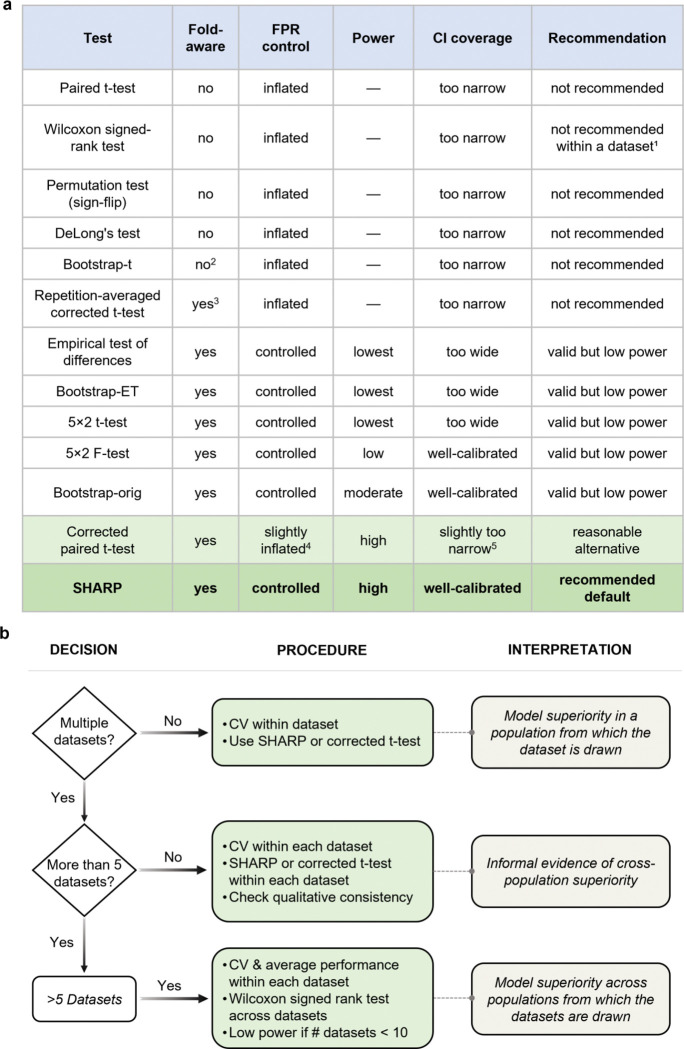
Guidelines for valid model comparison. **a.** Statistical properties of tests for comparing models in cross-validation. SHARP (highlighted) is recommended as the default for within-dataset comparison; the corrected resampled t-test is a reasonable alternative, especially when sample size is limited. **Footnotes:** ^1^The Wilcoxon signed-rank test is invalid within a dataset but valid for comparing dataset-level performance differences across datasets (Demšar, 2006; see panel b). ^2^Bootstrap-t resamples fold-level differences under an implicit independence assumption and is therefore invalid (see Methods Section 4.5.8). ^3^The repetition-averaged corrected resampled t-test uses an overly liberal assumption of between-fold correlation, leading to high FPR (see Supplementary Methods S9). ^4^FPR for the corrected resampled t-test is controlled in some regimes but mildly elevated in others. ^5^95% confidence interval for the corrected resampled t-test is well-calibrated in some regimes but mildly elevated in others. Power is reported as “—” for tests that do not control false positives at the nominal level, as power is not meaningfully defined in this case. **b.** Decision framework for selecting an appropriate statistical comparison procedure based on dataset availability. The flowchart guides users through sequential decisions (left) to arrive at a recommended procedure (middle) and the corresponding scope of inference that the resulting evidence supports (right).

We have established that statistical tests that neglect cross-validation fold dependence are widely used in biomedical publications, and such tests inflate false positive rates. Here we consolidate these findings into practical guidance. Fig. 8a summarizes the statistical properties of the tests evaluated in this study, and Fig. 8b translates these properties into a decision procedure extending from the single-dataset case — the focus of this paper — to the multi-dataset case.

When only a single dataset is available, SHARP is the recommended default (Fig. 8). It provided the best overall balance across the regimes we examined: reliable false-positive control, high statistical power, and well-calibrated confidence intervals. The corrected resampled t-test is also a reasonable choice, particularly when sample size makes SHARP’s split-half design less attractive. Further discussion about SHARP and corrected t-test are found in Section 3.5.

An alternative to cross-validation is a single train-validation-test split. Because the training set is fixed and the test set is used only once, test samples are independent and paired tests applied across test samples are valid. However, results can be highly sensitive to the particular split, (Varoquaux et al., 2017), so we do not recommend this procedure for small sample sizes.

A test within a single dataset supports inference only about the population from which the dataset was drawn. Access to multiple datasets enables broader inference. When 2–5 datasets are available, running a valid within-dataset test (SHARP or corrected t-test) on each dataset and checking for qualitative consistency provides *informal* evidence of cross-population superiority: a model that outperforms another across most or all datasets is more likely to be superior in new datasets drawn from the same populations (Fig. 8b).

When more than five datasets are available, a Wilcoxon signed-rank test across dataset-level performance differences (Demšar, 2006) supports formal cross-population inference (Fig. 8b). However, power is low when there are fewer than ten datasets. Furthermore, access to six or more comparable datasets is often infeasible in many areas of biomedical research, where even two or three can be difficult to obtain.

It is also important to note that cross-population inference might often not be the goal (Dietterich, 1998). Many domain-specific studies aim to identify the best-generalizing model within a given dataset, for example, to deploy in the population from which that dataset was drawn. In such settings, a valid within-dataset test (SHARP or corrected t-test) is the appropriate tool, and the inference it supports – generalization to the source population – is exactly what the study requires.

### 3.2 Invalid tests do not imply false findings

The fold-dependence problem concerns uncertainty quantification, not point estimation. Absent leakage or other circularity, cross-validated performance estimates remain valid estimates of generalization to the population from which the dataset was sampled. What fold dependence corrupts is the variance of those estimates — and hence p-values, confidence intervals, and any test statistic that relies on assumed independence.

While our meta-analysis focused on 210 studies, many others were excluded due to lack of methodological clarity, and these are unlikely to be free of invalid statistical tests. Although we restricted our analysis to journals with impact factors of at least 15, there is little reason to believe the issue is confined to this subset. Taken together, the scope of the fold-dependence problem likely extends to thousands of studies.

Going forward, we encourage researchers to adopt the inference procedures recommended in Fig. 8. However, for prior findings, an invalid statistical test does not imply a false positive: sufficiently large effects may remain significant under more conservative procedures, and any given model comparison may be only one component of a broader study whose overall conclusions remain valid. We therefore do not claim that 97% of studies contain false positives. Rather, 97% employed statistical tests that do not achieve the stated error control. This distinction is critical, and prior findings warrant reassessment rather than wholesale dismissal.

### 3.3 Widespread invalid tests reflect a knowledge gap, not a rigor gap

The dependence between cross-validation folds has been recognized for decades (Dietterich, 1998; Nadeau & Bengio, 2003; Bengio & Grandvalet, 2004), yet its neglect remains pervasive. The absence of any association with impact factor, transparency policies, or open science practices suggests a knowledge gap rather than a rigor gap. Existing safeguards — code sharing, reporting checklists, and data availability — cannot detect violations of statistical assumptions that are rarely taught and seldom reviewed. The issue is not that researchers are cutting corners, but that the dependence structure induced by resampling is not part of standard training in many biomedical disciplines.

Closing this gap will require changes at multiple levels. Graduate training should explicitly cover the statistical properties of cross-validation. Peer review guidelines should flag cross-validation-based statistical tests as requiring justification. Reporting standards should make it straightforward for readers to assess whether valid inference has been performed (see next section).

Intriguingly, researchers developing machine learning algorithms — as opposed to biomedical researchers employing them — mostly report point estimates across multiple datasets without formal statistical testing, despite established guidelines (Demšar, 2006; see Fig. 8b). This practice raises a complementary challenge: when performance differences are small, the absence of statistical inference makes it difficult to distinguish genuine improvements from noise, as recently demonstrated in medical imaging artificial intelligence (Christodoulou et al., 2025).

### 3.4 Recommendations for reporting standards

Our meta-analysis revealed substantial reporting deficiencies in the biomedical machine learning literature beyond the issue of fold dependence. Of 1,875 studies that compared model performance, 54% did not clearly apply a statistical test or report confidence intervals — either because no formal inference was conducted, or because reporting was too vague to tell. Among the remaining studies, many applied tests whose procedures were insufficiently described to evaluate, so they also had to be excluded.

Studies employing cross-validation for model comparison should clearly report: (1) the number of folds (e.g., 5-fold cross-validation), (2) the number of repetitions (e.g., 100 repeats), (3) the specific statistical test used, including whether it is one- or two-sided, and (4) how fold-level statistics are aggregated, including the size of the resulting input to the statistical test.

Clarifying the unit of analysis is particularly critical. For example, if 5-fold cross-validation is repeated 100 times, are the performance differences averaged within each repetition, yielding a vector of length 100 as input to the statistical test, or are all fold-level differences concatenated, giving a vector of length 500? These choices are not interchangeable: averaging within each repetition inflates the false positive rate of the misapplication of the corrected resampled t-test (Fig. S5; Supplementary Methods S9).

Bootstrap methods warrant similar attention. In our meta-analysis, bootstrap procedures were often described without sufficient detail to evaluate their validity. Authors should specify whether bootstrapping is applied to generate training and test sets or to resample cross-validation outputs, and how the resulting samples are used to compute p-values or confidence intervals. Treating bootstrapped samples as independent observations massively inflates the false positive rate, whereas using them as an empirical distribution yields valid inference (Fig. S5) — albeit with low power (Fig. S7).

Confidence interval reporting requires the same care. In our meta-analysis, we excluded all studies reporting only confidence intervals because too few specified how the intervals were computed. This ambiguity is consequential: an interval based on the standard error of fold-level statistics would incorrectly assume fold independence and be too narrow, while one based on the 2.5th and 97.5th percentiles of those statistics would be overly conservative (similar to the empirical test of differences). Authors should therefore specify the underlying variance estimator or resampling scheme.

### 3.5 SHARP and the corrected resampled t-test: practical considerations

SHARP builds directly on standard cross-validation by introducing a split-half step that eliminates overlap between paired statistics. In contrast to existing fold-aware tests, SHARP estimates the relevant variance and correlation from the data rather than imposing strong assumptions. We provide an open-source implementation compatible with scikit-learn, enabling adoption with minimal changes to existing workflows.

The primary limitation of SHARP is the reduction in effective sample size induced by the split-half procedure, which can be consequential when data are scarce. Different algorithms or biomarkers can exhibit distinct scaling behavior, such that their relative performance changes as training data increases. Because SHARP evaluates models using half the available data, it may not fully reflect their relative performance at the full sample size.

The corrected resampled t-test is a reasonable alternative. Its FPR control is mildly inflated in one simulation scheme (Fig. 6) but adequate in another (Fig. S4); its power is slightly below SHARP in the first scheme (Fig. 7a) and similar to SHARP in the second (Fig. S8); and its CI coverage is well-calibrated on average, though it shows substantial scenario-to-scenario variability in one scheme (Fig. 7b), likely reflecting its assumption about the magnitude of between-fold correlations.

Because the corrected resampled t-test uses the same inputs as standard cross-validation, it can be adopted with no methodological overhead. We therefore recommend it as a reasonable alternative to SHARP, especially when sample size is small.

### 3.6 Broader implications for reproducibility

A reproducibility crisis has been documented across multiple scientific fields, including psychology (Open Science Collaboration, 2015), cancer biology (Errington et al., 2021), and machine learning (Kapoor & Narayanan, 2023; Christodoulou et al., 2024). Common causes include p-hacking, selective reporting, and underpowered studies. The mechanism described here is different. Inflated false positive rates arise from a structural property of cross-validation – correlated folds – rather than from researcher incentives or analytical flexibility. Yet the consequence is similar: systematic overestimation of evidence that may fail to replicate.

A distinguishing feature of this class of problems is that it is unusually tractable. Addressing p-hacking or publication bias requires changes to incentives and research culture. By contrast, correcting for fold dependence requires only adopting appropriate statistical tests or evaluation procedures, which can be implemented without altering data collection or study design.

## 4 Methods

### 4.1 Primer on cross-validation

In this section, we provide an overview of different variants of cross-validation. In K-fold cross-validation, a dataset is divided into K non-overlapping partitions of data samples (folds). For each of K iterations, one fold is treated as the test set, while training is performed on the remaining folds. The procedure is repeated K times, so each fold has its turn to be the test. When comparing two models, the entire K-fold cross-validation procedure can be applied to each model separately. The K partitions are set up to be the same across the two runs of K-fold cross-validation, so that there is fold-level correspondence between the two models.

Within each fold, the performance of each model can be averaged across samples, yielding K pairs of performance metrics for the two models. For each pair of performance metrics, the difference can be computed, resulting in a vector D of J performance differences, where J=K. We note that the J performance differences are not independent because training sets overlap across iterations (for K>2) and the test set from one iteration contributes to the training set of all other iterations. Many studies apply a statistical test to the vector D of J performance differences to evaluate the null hypothesis of equivalent prediction performance between the two models.

For stability (Bouckaert & Frank, 2004; Varoquaux et al., 2017), many studies also repeat K-fold cross-validation R times. In this scenario, we still obtain a vector D of J performance differences, but J=K×R. We note that in this situation, there are two different fold-level correlations here. First, the fold-level statistics are correlated within a single instance of K-fold cross-validation. Across different repeats of K-fold cross-validation, there is now overlap in both training and test sets, so fold-level statistics from different repeats of K-fold cross-validation are correlated, and this correlation is likely different from the between-fold correlation within a particular instance of K-fold cross-validation. Once again, many studies apply a statistical test to the vector D of J performance differences to evaluate the null hypothesis of equivalent performance between the two models.

In Monte Carlo cross-validation, the datapoints that make up a dataset are repeatedly divided into training and test sets randomly. If the performance metric is averaged across samples in the test set and this procedure is repeated J times, we obtain a vector D of J performance differences. In this setup, there is overlap between both training and test sets across the J repeats, so the fold-level statistics are non-independent. Similar to the previous setups, many studies apply a statistical test to the vector D of J performance differences to evaluate the null hypothesis of equivalent performance between the two models.

In the above cross-validation schemes, the performance difference metric is averaged across all samples in a test set, so the statistical test utilizes fold-averaged statistics. During our meta-analysis, we observed some studies using “sample-level” instead of “fold-averaged” statistics. To illustrate the distinction, consider a study performing a single instance of 10-fold cross-validation on a dataset of 1,000 independent samples. A fold-averaged approach averages performance differences within each fold, yielding 10 performance differences metrics for the statistical test, i.e., the vector D has 10 values. A sample-level approach forgoes this aggregation and instead enters all 1,000 performance differences directly into the statistical test, i.e., the vector D has 1000 values. Correlations among performance differences in the same fold might be stronger than correlation between folds (since the trained model is the same within a fold), so sample-level statistics might further inflate the false positive rates.

A final variant collapses the fold-level estimates in the opposite direction: rather than disaggregating to individual samples, performance differences are averaged across folds within each repetition of the cross-validation, so that each repetition contributes a single value to the statistical test. We refer to these as “repetition-averaged” statistics, in parallel to the “fold-averaged” and “sample-level” statistics above. For example, if 10-fold cross-validation is repeated 30 times, a fold-averaged approach yields a vector D of length 300, whereas a repetition-averaged approach first averages the 10 fold-averaged performance differences within each repeat and then enters the resulting 30 values into the statistical test, i.e., the vector D has 30 values. The correlation among the 30 repetition-averaged statistics is larger than the correlation among the 300 fold-averaged statistics. Feeding this repetition-averaged vector into the corrected resampled t-test (Nadeau & Bengio, 2003; Methods Section 4.5.5) – which is calibrated for between-fold correlation, not the larger between-repetition correlation – leads to a high false positive rate (Supplementary Methods S9).

### 4.2 Meta-analysis

#### 4.2.1 Journal selection and search strategy

We conducted a literature search in PubMed on 18 September 2025 to identify original research articles that compared machine learning models using cross-validation. The search was restricted to articles published between 1 June 2020 and 1 June 2025. Eligible journals were defined using Journal Citation Reports (JCR) and were required to have a Journal Impact Factor (JIF) ≥ 15 in either 2023 or 2024. Review journals were excluded.

For each candidate journal, ISSN and eISSN information was obtained from JCR and matched to the corresponding National Library of Medicine (NLM) journal abbreviation in the NLM Catalog. Journals not indexed in the NLM Catalog were excluded, because PubMed searches by journal required this identifier. For each eligible journal, the following PubMed query was then executed:

(“outperform” OR “outperforms” OR “state-of-the-art” OR “benchmark” OR “benchmarking” OR “algorithm comparison” OR “model comparison” OR “performance evaluation” OR “method comparison”) AND (“cross validation” OR “cross-validation” OR “crossvalidation”) AND (“2020/06/01”[Publication Date] : “2025/06/01”[Publication Date]) AND (Journal Abbreviation [TA])

This search yielded 2,400 studies for subsequent screening (Fig. 2a).

#### 4.2.2 Eligibility criteria and PRISMA workflow

Screening of the 2400 studies followed PRISMA guidelines (Page et al., 2021). A final set of 210 studies met the following four criteria.

1. Direct model performance comparison.
  To satisfy this criterion, the study had to compare at least two distinct models – that might differ in terms of algorithms, pipelines, hyperparameter configurations, feature sets (e.g., biomarkers), or model-selection procedures – on the same prediction target. Eligible studies must compare models based on “direct” performance metrics (e.g., accuracy, AUC, MSE, MAE, correlation, etc.) computed from comparing predictions against ground truth. The models must be evaluated on the same test data. This criterion resulted in 1875 papers involving model comparisons and 525 without model comparisons (Fig. 2a).
  We excluded studies that compared only downstream quantities derived from predictions, as opposed to direct performance metrics. For example, a study might develop two brain-age models from MRI and compare them based on which model’s brain age predictions better differentiate Alzheimer’s disease from healthy individuals, as opposed to comparing models’ accuracy in predicting the training target (chronological age). Although the fold dependence problem would also arise in such cases, we excluded these studies because it was difficult to have a clear set of criteria for what downstream analyses to include or exclude in the meta-analysis.
2. Use of statistical inference for the comparison.
  The study had to report at least one p-value or confidence interval for a quantitative comparison of machine learning models. Studies that only reported descriptive statistics or performed statistical tests unrelated to model comparison (e.g., correlation between model predictions and confounding variables) were considered ineligible. This criterion resulted in 867 studies using statistical inference for comparing models, while 1008 studies were eliminated.
3. Fold-based statistical inference.
  Among the 867 studies, some did not use cross-validation, and a large fraction did not clearly describe their inference procedure. To be included in our final analyses, a study had to be clear that the values used to compute a statistical test or confidence interval were derived from the test folds of cross-validation. For example, we included studies that (i) performed K-fold cross-validation and used K pairs of values for statistical inference; or (ii) conducted K repeated random train–test splits (i.e., Monte Carlo cross-validation) and used K pairs of values for statistical inference. There were also studies that repeated K-fold cross-validation multiple times. For example, a study that repeated 10-fold cross-validation 100 times and used 1,000 pairs of values for a t-test, would be included in the meta-analysis. If such studies averaged performance across the 10 folds within each repetition and used the resulting 100 paired values in a t-test, they would also be included. The studies also had to clearly state the name of the statistical test. This criterion resulted in a set of 265 studies.
4. Use of statistical tests that ignore or account for fold dependence.
  Of the 265 studies, one employed a permutation test that was invalid for reasons unrelated to fold dependence; we therefore considered it outside the scope of the current study (see Supplementary Results for details). We also excluded studies that reported only confidence intervals, as most simply asserted the 95% interval without specifying how it was computed, a problem that has been independently documented in medical imaging (André et al., 2026). A 95% confidence interval based on the standard error of fold-level statistics would incorrectly assume fold independence, yielding an interval that is too narrow. Conversely, a 95% interval computed from the 2.5th and 97.5th percentiles of fold-level statistics approximates the empirical test of differences (Methods Section 4.5.7) and would be overly conservative. Without further methodological details, these studies could not be reliably analyzed. Applying these criteria resulted in a final set of 210 studies.

The PRISMA flow diagram describing identification, screening, eligibility assessment and inclusion is shown in Fig. 2a.

#### 4.2.3 Scientific field classification

To explore the prevalence of invalid statistical inference across scientific fields, we assigned each study to a scientific field. Scientific fields were defined using the Web of Science Journal Citation Reports (JCR) subject categories. JCR listed 254 categories, and each journal could belong to one or more categories. Because our PubMed-based search targeted biomedical and life-science content, not all JCR categories were represented. For journals assigned to one scientific category, all studies published in the journal were assigned to that category. For studies published in journals assigned to multiple categories (e.g., Nature), we used a large language model (LLM) to determine the most relevant category for each study. See Section 4.2.4 for more details about the LLM procedure, as well as the manual checks performed by human raters.

#### 4.2.4 LLM-assisted screening and manual human verification

To scale screening and data extraction, we used an LLM (Claude Opus 4.1, Anthropic) as an assistant in combination with manual human verifications. For each of 2400 studies, we provided the main text scraped from PubMed, including figure captions, but without figures, tables or supplementary material, together with a structured prompt. The full prompt text is provided in Supplementary Methods S1.1. The LLM was asked to answer five questions for each study:

1. **Scientific field classification (Q1).** If a study was published in a multi-field journal, the LLM was instructed to assign the study to a single scientific subfield using a predefined set of categories, or “OTHERS” if none applied.
  *Manual verification*: In a random audit of 50 studies, two human raters (TZ and HL) manually checked the studies. Any discrepancy was resolved by discussion and consensus. The LLM was in 100% agreement with the human raters. Therefore, we used the LLM results directly.
2. **Model comparison evaluation (Q2).** The LLM was asked to determine whether the study satisfied PRISMA criterion 1 (Methods Section 4.2.2).
  *Manual verification*: In a random audit of 50 studies, two human raters (TZ and HL) manually checked the studies. Any discrepancy was resolved by discussion and consensus. The LLM was in 100% agreement with the human raters. Therefore, we used the LLM results directly.
3. **Statistical inference (Q3).** If the LLM’s answer to Q2 was “Yes”, then the LLM was instructed to also identify every instance where a p-value or confidence interval was reported when comparing model performance. Articles with at least one instance were deemed eligible under PRISMA criterion 2 (Methods Section 4.2.2).
  *Manual verification:* In a random audit of 50 studies, two human raters (TZ and HL) manually checked the studies. Any discrepancy was resolved by discussion and consensus. The LLM agreed with two human raters (TZ and HL) on 39 studies (78%). For the remaining 11 studies, there was only one false negative, i.e., LLM counted a study as being ineligible under PRISMA criterion 2, but the human raters disagreed. We considered this level of false negatives to be acceptable. The remaining 10 errors were false positives, i.e., the LLM counted the studies as being eligible under PRISMA criterion 2, but human raters disagreed. Given the relatively high false positive rate, human raters read all 1197 studies that the LLM considered to satisfy PRISMA criterion 2. More specifically, each study was manually examined by two human raters (TZ, HL or SZ), and any discrepancy was resolved by discussion and consensus. After going through all studies, we ended up with 867 studies that satisfied PRISMA criterion 2.
4. **Statistical test details (Q4).** For each instance identified in Q3, the LLM was instructed to extract the name of the test or confidence interval, details about compared models, performance metric(s) used for the comparison, and the cross-validation procedure used. The LLM was asked to support its conclusions with direct quotations from the study.
  *Manual verification*: The extracted quotations served as a reference for human verification. However, we note that every study was manually examined by two human raters (TZ, HL or SZ) to re-derive the above information. Any discrepancy between human raters was resolved by discussion and consensus.
5. **Data classification (Q5).** For each instance identified in Q3, the LLM was also asked to classify whether the values entering the test were derived from cross-validation folds (referred to as “resampling units” in our prompt) or as “Other/Unclear”, corresponding to PRISMA criterion 3 (Methods Section 4.2.2).
  *Manual verification:* The LLM classification served as a reference for human verification. However, we note that every study was manually examined by two human raters (TZ, HL or SZ) to determine whether PRISMA criterion 3 was satisfied. Any discrepancy between human raters was resolved by discussion and consensus.

For each of the final set of 210 studies that satisfied all four PRISMA criteria, the human-verified information from Q4 and Q5 above was used to classify the article into one of two groups: (i) statistical tests ignoring fold dependence, and (ii) statistical tests accounting for fold dependence.

#### 4.2.5 Journal policy rigor scoring

To evaluate whether journal guidelines might influence the prevalence of invalid statistical inference, we defined a journal-level “policy rigor” score comprising two domains with a few criteria under each domain:

1. Transparency and reproducibility
  - *Code availability policy*: requirement or encouragement to share analysis code or model scripts.
  - *Data availability policy*: requirement or encouragement to share underlying or processed data.
  - *Data/code availability statement requirement*: requirement or encouragement for a formal data and/or code availability statement in the manuscript.
  - *Code for peer review*: requirement or encouragement to provide custom code to editors and reviewers during peer review.
2. Statistical rigor and methodological guidance
  - *Statistical test guidance*: explicit guidance on appropriate statistical testing (for example, paired vs unpaired tests, reporting of uncertainty, comparison under cross-validation).
  - *Statistical review*: whether dedicated statistical review is part of the editorial or peer-review process.
  - *Reporting standards or checklists*: requirement or encouragement to follow established methodological reporting guidelines (for example, TRIPOD-AI, CONSORT-AI, PRISMA).

In the case of transparency and reproducibility, journals were scored on a three-point scale for each of the four criteria: 0 = not mentioned or no guidance; 1 = encouraged or optional; 2 = mandatory or strongly enforced. Since there were four items, the total score ranged from 0 to 8.

In the case of statistical rigor and methodological guidance, journals were also scored on a three-point scale for each criterion with 0 corresponding to least rigor and 2 corresponding with most rigor. For more details about the scoring criteria, refer to Supplementary Table S1. Since there were three items, the total score ranged from 0 to 6.

To derive the scores, we used an LLM (GPT-5, OpenAI) to search each journal’s official webpages (Instructions for Authors, Editorial Policies, Submission Guidelines, Reporting Standards and related pages) on November 7, 2025 to identify the most authoritative policy statements. We changed the LLM from Anthropic to OpenAI, because the OpenAI LLM interface was more suitable for open web search at the time of the search. The LLM prompt is provided in Supplementary Methods S1.2.

Scoring from the LLM were verified by two human raters (TZ and HL). Any discrepancy between human raters was resolved by discussion and consensus. Finally, scores were summed across the two domains, resulting in an overall journal rigor score that ranged from 0 to 14. The score was used in analyses presented in Fig. 4. See Methods Section 4.2.7 for details about the statistical analyses.

#### 4.2.6 Study-level code and data availability

For each study, we used an LLM (Claude Opus 4.1, Anthropic) to determine whether the study provided data or code. Similar to Methods Section 4.2.4, for each of 210 studies, we provided the main text scraped from PubMed, including figure captions, but without figures, tables or supplementary material, together with a structured prompt. The full prompt text is provided in Supplementary Methods S1.3. In a random audit of 50 studies, the LLM was in 100% agreement with two human raters (TZ and HL). Therefore, the LLM results were used in analyses presented in Fig. 4. See Methods Section 4.2.7 for details about the statistical analyses.

#### 4.2.7 Test for temporal trend & association with scientific field, impact factor & scientific rigor

We tested for a trend in the publication rate of the 210 studies over time. Letting $Y_{i}$ be number of studies published in the i-th 6-month period, we fit a log-linear Poisson regression as follows:

$$Y_{i}\sim Poisson\left(\mu_{i}\right),\;ln\;\mu_{i}=\beta_{0}+\beta_{1}\times i;i=0,\;\ldots \;,9,$$

where $\beta_{1}$ quantifies the trend over time and $\beta_{0}$ is the intercept. If $\beta_{1}=0$, then this would suggest that the number of studies stayed constant over time. If $\beta_{1}$ is positive, then this means that volume of relevant studies is increasing. For example, in the current study, we found that $\beta_{1}=0.07(p=6e-3)$, so $e^{0.07}=1.070$. This suggests that the volume of relevant studies increased by approximately 7.0% per 6-month period, or 14.4% per year. The Poisson regression model was fitted using python package statsmodels 0.14 .5 with a p-value based on a Wald test.

We tested for the association between the study attribute “ignored fold dependence” and various factors. For ordinal factors of time, impact factor and journal rigor, we used a permutation-based Cochran–Armitage trend test. We did not use logistic regression for these analyses because the proportions of studies ignoring fold dependence were close to one, leading to a high prevalence of zero- and low-rate cells. The Cochran–Armitage test is sensitive to monotonic differences in the rate of studies that ignored fold dependence. Specifically, we constructed a $N_{rows}\;\times \;2$ contingency table in which rows indexed ordered strata (time intervals, impact-factor bins, or rigor levels) and columns counted studies that ignored versus accounted for fold dependence. Under the null hypothesis of no monotonic trend, we generated an empirical null distribution by repeatedly sampling 100,000 contingency tables conditional on the observed marginal totals (fixed row and column sums). For each sampled table we computed the Cochran–Armitage trend statistic. The two-sided p-value was estimated as the proportion of sampled Cochran-Armitage trend statistics whose absolute value was greater than or equal to the absolute value of the observed statistic.

For the nominal factors of scientific field, study-level code availability and study-level data availability, we used a permutation-based analogue of the Fisher–Freeman–Halton exact test. We first constructed a $N_{rows}\;\times \;2$ contingency table with rows representing levels of the nominal factor and the same two outcome columns as above. Under the null hypothesis of no difference between fields, we generated 100,000 tables conditional on the observed margins. For each sampled table, we computed the Pearson $\chi^{2}$ statistic as a measure of deviation from independence, forming an empirical null distribution. The p-value was estimated as the proportion of permuted $\chi^{2}$ values greater than or equal to the observed $\chi^{2}$.

### 4.3 Datasets

We evaluated the performance of statistical tests and confidence interval estimation using four datasets: EMNIST Digits (Cohen et al., 2017), UK Biobank (UKB; Alfaro-Almagro et al., 2018), Covertype (Blackard, 1998), and KEGG Metabolic Pathway (Muhammad Naeem, 2011). These datasets spanned image recognition, neuroimaging, ecological classification, and systems biology, enabling a comprehensive comparison of statistical inference approaches across distinct data modalities and learning tasks (classification and regression).

#### 4.3.1 EMNIST

We used the “digits” subset of the Extended MNIST (EMNIST) dataset (Cohen et al., 2017), which comprised 240,000 grayscale images (28×28 pixels) in the training set and 40,000 grayscale images (28×28 pixels) in the test set. For the current study, we only utilized the training set. There were 24,000 images per digit class (0–9). The machine learning task was to classify each image into one of the 10-digit classes.

#### 4.3.2 UK Biobank

We analyzed data from 36,454 UK Biobank participants (Alfaro-Almagro et al., 2018), consistent with our previous studies (He et al., 2022; Wulan et al., 2024). The prediction task was regression, with age (defined as MRI scan date minus birth year and month) as the target variable. The volumes of 101 cortical and subcortical gray-matter regions generated by the FreeSurfer software (Fischl, 2012) were features used for prediction. Raw volumetric measures were first normalized by dividing by the intracranial volume of each participant. Feature z-normalization was then performed using the mean and standard deviation computed from a held-out subset of 454 randomly sampled participants, which were subsequently applied to the remaining 36,000 participants. This yielded a final dataset of 36,000 individuals, each represented by 101 standardized features.

#### 4.3.3 Covertype

The Covertype dataset enabled the benchmarking of forest cover type classification based on cartographic variables derived from remote sensing and US Forest Service data. Each instance corresponded to a 30×30 meter cell in one of four wilderness areas within the Roosevelt National Forest, Colorado (Blackard, 1998).

The dataset contained 581,012 instances and 54 features, including both continuous variables (elevation, slope, aspect, hillshade values, distance to hydrology/roads) and binary variables encoding soil types and wilderness area indicators. Labels represented one of seven forest cover types, derived from the USFS Region 2 Resource Information System (RIS).

Following previous studies (Collobert et al., 2001), we constructed a binary classification task: class 1 included all cover types except for type 2, while class 2 included only cover type 2 (commonly associated with lodgepole pine in Rawah and Comanche Peak). This transformation resulted in a more balanced distribution: 297,711 samples in class 1 and 283,301 samples in class 2.

#### 4.3.4 KEGG Metabolic Pathway

The KEGG (Kyoto Encyclopedia of Genes and Genomes) Metabolic Pathway database describes interactions between biochemical compounds, enzymes, and genes. These pathways can be represented as graphs using two models: (i) the reaction network, where nodes correspond to substrates and products, and edges correspond to catalyzing genes or enzymes; and (ii) the relation network, where compounds form edges between gene/enzyme nodes (Muhammad Naeem, 2011). A large set of pathway entries were parsed and transformed into graphs using both representations.

The dataset provided graph-based features extracted using Cytoscape (Shannon et al., 2003), a software platform for biological network visualization and analysis. There were a total of 53,414 samples, each described by 20 topological features (e.g., degree centrality, betweenness centrality, and clustering coefficient). In the current study, the learning objective was to predict the clustering coefficient of each network instance, making this a regression task.

### 4.4 Simulations to evaluate FPR, statistical power and confidence intervals

#### 4.4.1 Simulation procedure for evaluating false positive rates (FPR)

We conducted analyses on four datasets — EMNIST, UKB, Covertype, and KEGG Metabolic Pathway — using four sample sizes: N=100,500,1,000, and 2,000. For EMNIST, N=100 was excluded because with 10 digit classes, it was not possible to guarantee at least one instance of each class in the test set under our proposed SHARP test, which required a split-half step within the cross-validation scheme (see Methods Section 4.6). For UKB, N=2,000 was excluded because the maximum number of non-overlapping sampled datasets that could be drawn from UKB at this sample size was fewer than 20 (i.e., 36,000 / 2,000 = 18), which we considered too few to yield stable false positive rate estimates.

We drew m non-overlapping sampled datasets of size N from the full dataset. The value of m was set to 100 when feasible; otherwise, it was set to the maximum number of non-overlapping sampled datasets that could be drawn. For example, for the UK Biobank dataset at N=1,000, this yielded m=36 (i.e., 36,000 / 1,000).

For each sampled dataset of size N, two noisy versions were generated by independently injecting the same level of random noise. Noise was introduced by permuting the target variable (e.g., digit labels for EMNIST) for a fixed proportion of samples. These were referred to as Noisy Dataset 1 and Noisy Dataset 2. We tested 10 permutation percentages in total: 10% to 100% with an increment of 10%.

For each noisy version of the sampled dataset of size N, we applied (i) a single instance of K-fold cross-validation or (ii) multiple repeats of K-fold cross-validation or (iii) Monte Carlo cross-validation. In classification tasks, stratified splitting was used to preserve label proportions across folds. All data splits were kept identical across the original sampled dataset and the two noisy datasets to ensure correspondence for paired statistical tests. We note that the SHARP test utilized a different cross-validation scheme (see Methods Section 4.6 for details).

A single algorithm was selected for each dataset based on initial exploration using the PyCaret Python package (Ali, 2020): logistic regression classifier for EMNIST, ridge regression for UK Biobank, Extra Trees classifier for Covertype, and Extra Trees regression for KEGG Metabolic Pathway (see Methods Section 4.4.4). For each noisy version of the sampled dataset, the algorithm was trained on the training folds and evaluated on the test fold of the original (unpermuted) sampled dataset. Evaluating both models on the same unpermuted test data ensured fair comparison between the two noisy-trained models.

For every training-test split, a pair of fold-level performance metrics were computed for each pair of noisy datasets, and the model performance differences between the two noisy datasets were computed, resulting in a vector D comprising J fold-level differences (except for SHARP; see Methods Section 4.6). For example, if K-fold cross-validation was repeated R times, then vector D will be of length J=K×R. For a given two-sided statistical test (e.g., paired t-test), the vector D was used to evaluate the null hypothesis μ=0, where μ is the true expected difference between the pair of trained models.

Since the noise level and algorithm were held constant across the pair of noisy datasets, the two trained models had identical expected predictive performance. Any rejection of the null hypothesis constituted a false positive. The false positive rate (FPR) was estimated as the fraction of m sampled datasets in which the null hypothesis was rejected at a significance threshold of 0.05. A well-calibrated test should reject the null hypothesis no more than 5% of the time. Recall that the m datasets were non-overlapping, so traditional binomial methods can be used to compute confidence intervals for the FPR. Specifically, the 95% confidence interval for the FPR was computed using the Wilson score interval with continuity correction (Newcombe, 1998); the formula is provided in Supplementary Methods S2.

Depending on the analysis, K-fold cross-validation was either performed once (R=1) or repeated R times. When repeated, we targeted a total of 300 folds (K×R=300) to ensure convergence of the statistical tests — for example, R=30 for 10-fold and R=60 for 5-fold cross-validation, so J was 300. For Monte Carlo cross-validation, we always used an 80/20 train-test split repeated 300 times, so J was again 300. The exception was the 5×2 statistical tests, which by design required 2-fold cross-validation repeated 5 times, so J=10.

We note that given 10 noise levels (10% to 100%) and sample size (N=100 to 2,000) across four datasets, there were 140 conditions. For each condition, we considered three hyperparameter-selection strategies: (1) fixed hyperparameters, (2) hyperparameter selection via a single training-validation split of the training data, and (3) nested cross-validation, in which hyperparameters were selected using cross-validation within the training data (see Methods Section 4.4.4). This resulted in 140 × 3 = 420 simulation scenarios in total. The FPR results are reported in Figs 5 and 6.

#### 4.4.2 Simulation procedure for evaluating statistical power

The simulations to benchmark statistical power followed the same procedure as those for FPR (Section 4.4.1), with one key difference. For FPR, the same algorithm was trained on two equivalently noisy datasets, so that the resulting pair of models was expected to have equivalent predictive performance. To assess statistical power, the same algorithm was instead trained on one noisy dataset and one original (unpermuted) sampled dataset. In this case, the model trained on the original (clean) sampled dataset was expected to outperform the model trained on the noisy dataset. A sensitive test should reject the null hypothesis $H_{0}:\;\mu \;=\;0$ in a two-tailed test, where μ is the true expected difference in predictive performance between the two trained models.

As in the FPR case, power was estimated as the fraction of m datasets in which the null hypothesis was rejected at a significance threshold of 0.05. Crucially, a rejection was counted as a true positive only when the original-dataset model was found to significantly outperform the noisy-dataset model; cases in which the test rejected the null in the opposite direction were not counted as successes. Power was then averaged across 420 scenarios, yielding an average power for each statistical test.

#### 4.4.3 Confidence Intervals for Performance Difference

While there are different methods to compute a confidence interval (CI), here we used test-inversion which defines the CI by the set of null-hypothesis parameter values that cannot be rejected.

Recall that there were 420 simulation scenarios with each scenario corresponding to a given dataset (e.g., EMNIST), sample size (e.g., 2000), noise permutation percentage (e.g., 20%) and hyperparameter setting (e.g., fixed hyperparameters). For each scenario, there were m (non-overlapping) sampled datasets. For each sampled dataset, we injected noise to it, resulting in a noisy dataset. In the power simulations (Methods Section 4.4.2), the same algorithm was trained on one noisy dataset and one original (unpermuted) sampled dataset. The model trained on the original sampled dataset was expected to outperform the model trained on the noisy data.

Following the power simulations (Methods Section 4.4.2), for a given pair of original and noisy datasets, we performed cross-validation to obtain a vector D of J fold-level prediction performance differences, with mean $\bar{D}$. We applied various statistical tests to the vector D to evaluate the null hypothesis μ=0, where μ is the true expected difference between the pair of trained models.

To obtain the 95% confidence interval for μ for a given statistical test (e.g., paired t-test), we tested null hypotheses $\mu =\mu_{0}$ for different values of $\mu_{0}$ to discover the range of values such that p≥0.05. This search was achieved by starting with $\mu_{0}=\bar{D}$, and performing a binary search in the direction larger than $\bar{D}$ and in the direction smaller than $\bar{D}$. The set of non-significant parameter values, $\left[\mu_{L},\mu_{U}\right]$, is the 95% confidence interval for the performance difference between the two models.

Ideally, we would evaluate the accuracy of the computed 95% confidence intervals by comparing with the true difference $\mu_{\text{true}}$, but this is not observable. Instead, we averaged $\bar{D}$ across all m non-overlapping sets, resulting in the estimate ${\overset{ˆ}{\mu }}_{\text{true}}$. Across the m confidence intervals, we counted the fraction of times ${\overset{ˆ}{\mu }}_{\text{true}}$ fell within the confidence intervals, which we referred to as the overall coverage rate. A well-calibrated 95% confidence interval will cover $\mu_{\text{true}}$ 95% of the time. The whole procedure was repeated 420 times, once for each scenario.

#### 4.4.4 Choice of algorithms, metrics and hyperparameter tuning schemes

To determine an algorithm for each dataset, we used the python package PyCaret (Ali, 2020) to explore a set of algorithms available in the scikit-learn package with default hyperparameters (Pedregosa et al., 2011). We preferred algorithms that performed well on the original data, ensuring room for performance to decrease when trained on noisy data under our FPR and power simulation schemes. Computationally intensive algorithms with good performance were excluded (e.g., CatBoost).

We considered three hyperparameter tuning strategies. The first strategy used fixed hyperparameters, where a single predetermined configuration was applied based on scikit-learn defaults. The second strategy used an internal 80%/20% train-validation split within the training set. For each candidate hyperparameter value, the model was trained on the 80% subset and evaluated on the 20% validation set. The training-validation split was fixed across candidates, so validation performance was comparable. The hyperparameter value with the best validation performance was then used to train a final model on the full training set, which was applied to the test set.

The third strategy used nested cross-validation. For each candidate hyperparameter value, 2-fold cross-validation was performed on the training set. The cross-validation split was fixed across candidates, so performance was comparable. The hyperparameter value with the best cross-validation performance was then used to train a final model on the full training set, which was applied to the test set.

For the EMNIST dataset, where the goal was to classify images into one of 10-digit classes, we used multi-class logistic regression classifier. The performance metric was classification accuracy, defined as the fraction of samples in the test set that was classified correctly. The hyperparameter of interest was C, the inverse regularization strength. In the fixed hyperparameter setting, C was set to 1.0, while in the hyperparameter tuning settings, C was searched across the following values: [0.0001, 0.0005, 0.001, 0.005, 0.01, 0.05, 0.1, 0.5, 1, 5, 10, 50, 100, 500, 1000, 5000, 10000].

For the UKB dataset, where the goal was to predict age of the participant, we applied linear ridge regression. The performance metric was the coefficient of determination in the test set (Wright, 1921). The hyperparameter of interest was the regularization parameter α. In the fixed hyperparameter setting, α was set to 10, and for hyperparameter tuning, α was searched across the following values: [0.0001, 0.0005, 0.001, 0.005, 0.01, 0.05, 0.1, 0.5, 1, 1.5, 2, 3, 4, 5, 10, 15, 20, 30, 40, 50, 100, 150, 200, 300, 400, 500, 1000, 2000, 5000, 10000].

For the Covertype dataset, where the goal was to classify samples into one of two cover type classes, we used the Extra Trees classifier. The performance metric was classification accuracy, defined as the fraction of samples in the test set that was classified correctly. In the fixed hyperparameter setting, we used scikit-learn defaults with 100 estimators. For hyperparameter tuning, ranges were chosen following prior studies (Komer et al., 2014; Grinsztajn et al., 2022) and optimized using the Tree-structured Parzen Estimator (TPE; Bergstra et al., 2011, 2013).

For the KEGG Metabolic Pathway dataset, where the goal was to predict the clustering coefficient of each network sample, we applied the Extra Trees regressor. The performance metric was the mean squared error, computed by averaging the squared error across all test samples. In the fixed hyperparameter setting, we used scikit-learn defaults with 100 estimators. For hyperparameter tuning, ranges were chosen following prior studies (Komer et al., 2014; Grinsztajn et al., 2022) and optimized using the Tree-structured Parzen Estimator (TPE; Bergstra et al., 2011, 2013).

Across the four datasets, we evaluated four sample sizes each, with the exception of EMNIST and UKB, for which only three sample sizes each were considered (see Methods Section 4.4.1). Combined with ten noise levels and three hyperparameter optimization schemes, this yielded (4 + 4 + 3 + 3) × 10 × 3 = 420 simulation scenarios in total.

The KEGG Metabolic Pathway dataset was the only dataset in which performance was recorded at the individual sample level (squared error per test sample), rather than aggregated across the entire test set. This made it the only dataset suitable for comparing fold-averaged and sample-level statistical tests (Results Section 2.5). For this analysis, the combination of four sample sizes, ten noise levels, and three hyperparameter optimization schemes yielded 4 × 10 × 3 = 120 scenarios.

### 4.5 Existing hypothesis testing approaches for cross-validation

We consider the problem of testing whether one machine learning model statistically outperforms another machine learning model in a given dataset based on cross-validation. For example, in K-fold cross-validation, the dataset of N samples is partitioned into K mutually exclusive folds. Each fold serves once as a test set, while the remaining K-1 folds form the training set. The training set is used to train each model, and model performance is evaluated in the test set. This yields K pairs of performance metrics on which we want to perform a statistical test.

If we repeat K-fold cross-validation R times, then we have J=K×R pairs of performance metrics. In general, we assume the statistical test operates on a vector D (of length J), where each element of vector D corresponds to a fold-level performance difference between the two models for a particular test fold.

For example, in the case of performing 10-fold cross-validation once, the vector D will be of length J=10. If we perform 10-fold cross-validation 30 times, vector D will be of length J=300. In the case of 80–20 Monte Carlo cross-validation where the dataset is repeatedly split into 80% training set and 20% test set 300 times, the vector D will again be of length J=300.

In the following subsections, we briefly summarize various statistical tests used in the literature. We note that the resampled paired t-test (Section 4.5.1), Wilcoxon signed-rank test (Section 4.5.2), permutation test (Section 4.5.3) and DeLong’s test (Section 4.5.4) are expected to have elevated FPR. The corrected resampled t-test (Section 4.5.5), the 5×2 tests (Section 4.5.6) and the empirical test of differences (Section 4.5.7) implicitly or explicitly try to account for fold dependence. The bootstrap may or may not be valid depending on how it was implemented (Section 4.5.8).

#### 4.5.1 Resampled paired t-test

As demonstrated in our meta-analysis, the most common statistical test used in comparing models in cross-validation is the paired t-test on the fold-level accuracy differences, referred to as the “resampled paired t-test” (Nadeau & Bengio, 2003). Given a vector D of J fold-level performance differences between two models, we test the null hypothesis μ=0, where μ is the true expected performance difference between the two models. The paired t-test utilizes the following statistic:

$$T=\frac{\bar{D}}{\sqrt{\frac{1}{J}S^{2}}}$$

where $\bar{D}$ is the mean of vector D, $S^{2}=\frac{1}{J-1}\sum_{j}{\left(D_{j}-\bar{D}\right)}^{2}$ is the sample variance of vector D,

The paired t-test assumes independence among the elements of vector D, an assumption that is violated under cross-validation. In K-fold cross-validation, test folds are mutually disjoint across iterations, but the training sets overlap substantially (for K>2), and the test fold in one iteration contributes to the training set in another iteration. In repeated K-fold cross-validation, the folds are redefined, but the cross-dependence remains. Similarly, in Monte Carlo cross-validation, where the dataset is repeatedly split into training and test sets, there will be overlap of both training and test sets across random dataset splits.

Because the correlations among fold-level statistics are positive, the paired t-test is expected to have elevated false positive rates, as demonstrated empirically in Fig. 5. A more detailed discussion of this issue can be found in Supplementary Methods S3.

#### 4.5.2 Wilcoxon signed-rank test

As demonstrated in our meta-analysis, the second most common statistical test used in comparing models in cross-validation is the Wilcoxon signed-rank or Wilcoxon rank-sum test. The Wilcoxon rank-sum test is also referred to as the Mann–Whitney U test, and is the unpaired version of the Wilcoxon signed-rank test. The Wilcoxon signed-rank test is a nonparametric alternative to the paired t-test, designed to test whether the median of paired differences is zero (Wilcoxon, 1945; Sidney, 1957).

Similar to the paired t-test, the Wilcoxon signed-rank test takes in a vector of paired differences and ranks the absolute differences between the J paired observations. The test then computes a test statistic based on the sum of these ranks, weighted by the sign of the original differences. The resulting test statistic is then compared against a reference distribution to obtain a p-value (Japkowicz & Shah, 2011).

Like the paired t-test, the Wilcoxon signed-rank test assumes independence among the paired differences. Because the fold-level statistics are positively correlated, the Wilcoxon signed-rank test is expected to have elevated false positive rates, as demonstrated empirically in Fig. 5.

#### 4.5.3 Sign-flip permutation test

We found that there are two different permutation tests used in literature, but both are invalid. One permutation test is invalid not because of fold dependence but because the wrong variables are permuted. We excluded this permutation test from our meta-analysis. For details, see Supplementary Results.

We will describe the second permutation test here. Once again, the null hypothesis is μ=0, where μ is the true expected difference between the two models. Given a vector D of J fold-level performance differences between two models, let $\bar{D}$ be the mean of vector D. A permutation of the data generates a null distribution: For each iteration, the sign of each element of vector D is flipped independently with probability 0.5 , thus creating a new vector $D_{0}$. The mean of $D_{0}$ is then computed, contributing a single null value to the null distribution. After many iterations (e.g., 10,000 ), the original statistic $\bar{D}$ is compared against the null distribution to generate a p-value.

This permutation test wrongly assumes the elements of vector D are independent. Therefore, similar to the naïve t-test, we expect the permutation test to yield higher FPR, as demonstrated empirically in Fig. S5. A more detailed discussion of this issue can be found in Supplementary Methods S8.

#### 4.5.4 DeLong’s test

The DeLong’s test is a statistical procedure specific to model comparison based on a Receiver Operating Characteristic (ROC) curve (DeLong et al., 1988). The test only applies to binary classification tasks, in which the model predicts one of two possible outcomes (e.g., spam vs. non-spam, disease vs. no disease). In such tasks, classifier performance can be summarized by an ROC curve, which plots the True Positive Rate (TPR) against the False Positive Rate (FPR) across different decision thresholds. To compare two ROC curves, the Area Under the ROC Curve (AUC) is used as a scalar performance metric, with larger AUC values indicating better performance.

DeLong’s test evaluates whether the difference in AUCs between two algorithms is statistically significant. Specifically, the test statistic is constructed as the difference between the two estimated AUCs divided by an estimate of its standard error, yielding an asymptotic z-statistic. The AUC estimator is a function of the pairwise comparisons between positive and negative samples and can be expressed as a normalized Mann–Whitney U-statistic. DeLong’s test estimates the 2×2 covariance matrix of the two AUC estimators using an influence-function-based approach for vectors of U-statistics (DeLong et al., 1988). The joint asymptotic normality of the AUC estimators justifies the use of a normal approximation to compute p-values.

Although the variance estimation in DeLong’s test is mathematically more involved than that of simpler tests, the U-statistic variance derivation underlying DeLong’s test assumes that predictions are independent across data samples, which is violated under cross-validation. We also note that the DeLong’s test is based on sample-level statistics, as opposed to fold-averaged statistics (see Methods Section 4.1). As such, we expect the DeLong’s test to have elevated FPR, as empirically shown in Fig. S1.

#### 4.5.5 Corrected resampled paired t-test

The corrected resampled paired t-test was proposed to adjust the variance estimator to reflect the positive correlation between cross-validation folds (Nadeau & Bengio, 2003). Although originally developed for Monte Carlo cross-validation, the corrected paired t-test has also been applied to repeated K-fold cross-validation (Bouckaert & Frank, 2004).

Similar to previous tests, the corrected resampled t-test operates on the vector D of J fold-level performance differences between two models. Again, we wanted to test the null hypothesis μ=0, where μ is the true expected difference between the two models. The corrected resampled t-test assumes that the correlation between folds is equal to the fraction of the full dataset used in the test fold, resulting in the following statistic:

$$T=\frac{\bar{D}}{\sqrt{\left(\frac{1}{J}+\frac{N_{2}}{N_{1}}\right)S^{2}}},$$

where $\bar{D}$ is the mean of vector D, $S^{2}=\frac{1}{J-1}\sum_{j}{\left(D_{j}-\bar{D}\right)}^{2}$ is the sample variance of vector D, and $D_{j}$ refers to the j-th element of vector $D;\;N_{1}$ and $N_{2}$ are the number of training and test samples, respectively, in a particular split of the dataset into training and test sets. The statistic T is assumed to follow a Student’s t distribution with J-1 degrees of freedom, from which a p-value can be computed.

A more detailed discussion of the corrected resampled t-test can be found in Supplementary Methods S3. One problem with the corrected resampled t-test is the assumption that the correlation between cross-validation folds is solely due to the overlap in the test set. In reality, the correlation likely depends on the complex interaction between the machine learning algorithms and the dataset being analyzed. We also note that some studies applied the corrected resampled t-test wrongly, resulting in a high FPR (Fig. S5; Supplementary Methods S9).

#### 4.5.6 5×2 paired t-test and 5×2 paired F-test

The 5×2 t-test (Dietterich, 1998) and 5×2 F-test (Alpaydin, 1999) involve 5 repetitions of 2-fold cross-validation, hence the name 5×2. For each repetition r∈{1,2,3,4,5}, the data is randomly split into two halves A and B. Both models are trained on set A and evaluated on set B, resulting in performance difference $D_{Br}$; likewise, both models are trained on set B and evaluated on set A, resulting in performance difference $D_{Ar}$.

The 5×2 t-test utilizes the following statistic (Dietterich, 1998):

$$T=\frac{D_{A1}}{\sqrt{\frac{1}{5}\sum_{r=1}^{5}S_{r}^{2}}}$$

where $S_{r}^{2}={\left(D_{Ar}-D_{Br}\right)}^{2}/2$. The statistic is assumed to follow a Studenťs t distribution with 5 degrees of freedom, from which a p-value can be computed. Supplementary Methods S4.1 explains why the 5×2 t-test will have a much lower false positive rate than the naïve t-test. However, the numerator in the T statistic $D_{A1}$ only uses a single accuracy difference from the first fold of the first 2-fold cross-validation, thus discarding information from the other folds.

To address the inefficiency of the 5×2 t-test, Alpaydin (Alpaydin, 1999) proposed the 5×2 paired F-test, which uses the following statistic:

$$F=\frac{\sum_{r=1}^{5}\left(D_{Ar}^{2}\;+\;D_{Br}^{2}\right)}{2\sum_{r=1}^{5}S_{r}^{2}}$$

where $S_{r}^{2}={\left(D_{Ar}-D_{Br}\right)}^{2}/2$ (same as the 5×2 t-test). The statistic is assumed to follow the F-distribution with 10 and 5 degrees of freedom in the numerator and denominator respectively, from which a p-value can be defined. Supplementary Methods S4.2 explains why the 5×2 F-test will have a much lower false positive rate than the naïve t-test. Intuitively, since the numerator uses fold-level differences from all 5 repetitions of 2-fold cross-validation (as opposed to just a single fold in the 5×2 t-test), the 5×2 F-test might be a more sensitive test than the 5×2 t-test (Alpaydin, 1999).

Indeed, our results suggest that both the 5×2 t-test and 5×2 F-test reliably control FPR, and that the 5×2 F-test exhibits higher power than the 5×2 t-test. However, both tests had lower power than SHARP.

#### 4.5.7 Empirical test of differences

Given a vector D of J fold-level performance differences between two models, suppose the number of positive entries is greater than the number of negative entries. The empirical test of differences then computes the p-value as the fraction of negative entries multiplied by two.

On the other hand, suppose the number of positive entries is less than the number of negative entries. The empirical test of differences then computes the p-value as the fraction of positive entries multiplied by two.

The empirical test of differences has been utilized by a few brain imaging studies (Dhamala et al., 2021; Parkes et al., 2021a; Parkes et al., 2021b), which referred to it as the exact test of differences. Our results suggest that the empirical test of differences reliably controls false positives (Fig. 6) while having the lowest power among statistical tests that account for fold dependence (Fig. 7). Supplementary Methods S5 provides a theoretical explanation for these findings.

#### 4.5.8 Three bootstrap variants

Bootstrap is a common technique for estimating confidence intervals (Efron & Tibshirani, 1994; DiCiccio & Efron, 1996). There are different bootstrapping variants for cross-validation (Raschka, 2018; Cai et al., 2025). Our current meta-analysis excluded all studies that only reported a confidence interval (PRISMA criterion 4; Methods Section 4.2). As such, all studies using bootstrap were also excluded. However, we note that only one study described their bootstrapping procedure in sufficient details for us to evaluate its validity (Peneder et al., 2021).

Below we outlined three broad bootstrap strategies that we evaluated in the current study. The three strategies were not meant to be exhaustive but were aimed to cover the good and bad variants of bootstrap. First, we implemented the bootstrap procedure from Raschka (2018), since it coincided with the exemplary study in our meta-analysis that clearly described the procedure (Peneder et al., 2021). More specifically, given a dataset of N samples, we bootstrapped (sampled with replacement) N samples from the dataset, which served as the training set. We note that on average, 63% of the training samples will be unique. The test set comprised samples that were not included in the training set.

To compare two models, the bootstrapped training set was used to train each model and the trained models were evaluated on the test set, yielding a single bootstrapped difference value. The entire bootstrapping procedure was repeated M times, and the 95% confidence interval was constructed from the M performance difference estimates based on the 2.5 and 97.5 percentiles. If the 95% confidence interval did not cover zero, we considered the difference to be statistically significant. We referred to this bootstrapping procedure as “bootstrap-orig”. For the evaluation of FPR and power, we followed the procedure in Section 4.4 with M being set to 300.

The other two bootstrapping procedures performed bootstraps after normal cross-validation. For both approaches, given a vector D of J fold-level performance differences, bootstrapping (sampling with replacement) was performed on the entries of vector D, yielding a new vector $D^{*}$ of 1000 bootstrapped fold-level differences. An empirical test of differences (Methods Section 4.5.7) was applied to $D^{*}$, which we referred to as bootstrap-ET. Alternatively, the naïve paired t-test (Section 4.5.1) was applied to $D^{*}$, which we referred to as bootstrap-t.

Our results suggest that bootstrap-orig and bootstrap-ET reliably control FPR, but bootstrap-t has high FPR (Fig. S5). However, both bootstrap-orig and bootstrap-ET had markedly worse power than SHARP, with bootstrap-ET being the least powerful (Fig. S7).

### 4.6 Split-HAlf RePeated (SHARP) test

The challenge of inference on performance differences comes down to estimating the standard error of the sample mean difference while accounting for the dependence between the J differences. Perhaps the clearest illustration of the problem is to consider a single application of K-fold cross-validation (discussed in Section 2.6): The J entries in the vector D of fold-level differences are correlated, and for Gaussian data, there are two sufficient statistics (sample mean and sample variance), but three unknown parameters (mean, variance, and correlation). Thus, the model is overparameterized and, specifically, the variance and correlation cannot both be estimated without restrictive assumptions. The same fundamental statistical ambiguity applies to Monte Carlo cross-validation.

In their seminal study, Bengio and Grandvalet (Bengio & Grandvalet, 2004) considered a single instance of K-fold cross-validation and worked from sample-level prediction errors instead of fold-averaged performance (see Methods Section 4.1). They showed that there is no universal, distribution-independent unbiased estimator of the standard error. In particular, for Gaussian data, the maximum likelihood estimator does not exist. The covariance structure in their setting (based on sample-level prediction performance) is equivalent to the covariance structure of fold-averaged prediction performance under repeated K-fold cross-validation, so their findings generalize to repeated K-fold cross-validation.

The key idea behind the SHARP test is to create a situation where independent data are generated that will allow estimation of the variance parameters $\sigma^{2}$ and ρ. To achieve this, we randomly divide the dataset into two disjoint halves A and B. For each machine learning model, we then perform K-fold cross-validation in subsets A and B separately. We then average the results across the K-fold cross-validation, resulting in a pair of model performance differences, $D_{A1}$ and $D_{B1}$ respectively. Alternatively, one iteration of Monte Carlo cross-validation can be done within each subset A and B, likewise producing a pair of performance $D_{A1}$ and $D_{B1}$ based on the held-out testing data in each half.

We note that $D_{A1}$ and $D_{B1}$ are independent since subsets A and B are non-overlapping. For the analyses in the current study, this process is repeated J times, resulting in two vectors $D_{A}=\left[D_{A1},\;\ldots \;,D_{AJ}\right]$ and $D_{B}=\left[D_{B1},\;\ldots \;,D_{BJ}\right]$. We note that while $D_{Aj}$ and $D_{Bj}$ (from the j-th iteration) are independent, the entries within $D_{A}$ (and $D_{B}$ ), and $D_{Aj}$ and $D_{Bj^{\prime }}$ for $j\neq j^{\prime }$ are dependent with a common correlation. Therefore, we have J pairs of independent observations and three unknowns: mean performance μ, the variance $\sigma^{2}$ of each entry in $D_{A}$ and $D_{B}$, and correlation ρ among all non-paired entries in $D_{A}$ and $D_{B}$. Given $D_{A}$ and $D_{B}$, we can estimate all three unknowns to perform the statistical test.

We define $\bar{D}$ to be the grand average of the two vectors $D_{A}$ and $D_{B}$. We seek to test the null hypothesis of equal expected performance:

$$H_{0}:\mu =0,\;\text{where}\;\mu =E[\bar{D}]$$

We show that the SHARP estimator of performance difference

$$\bar{D}=\left({\bar{D}}_{A}+{\bar{D}}_{B}\right)/2,$$

where ${\bar{D}}_{A}$ and ${\bar{D}}_{B}$ are the respective sample means, is the generalized least-squares estimator of μ (Supplementary Methods S7.2), with variance

$$Var(\bar{D})=\sigma^{2}\left(\frac{1}{2J}+\frac{J\;-\;1}{J}\rho \right)$$

and so inference reduces to estimating $\sigma^{2}$ and ρ. To estimate $\sigma^{2}$ and ρ, we considered method-of-moments (Supplementary Methods S7.3), maximum likelihood (Supplementary Methods S7.4) or restricted maximum likelihood (Supplementary Methods S7.5), followed by a Wald test. We also considered likelihood-based tests including score test (Supplementary Methods S7.6) and likelihood ratio test (Supplementary Methods S7.7).

Based on simulated data (Supplementary Methods S7.8), we found that the score test provided the best control of FPR (Fig. S11). Therefore, all results in the current study utilized the score test. For the score test, the Z-score is a ratio of $\bar{D}$ to a standard error computed using $Var(\bar{D})$ above, but with estimates ${\overset{ˆ}{\sigma }}_{0}^{2}$ and ${\overset{ˆ}{\rho }}_{0}$ obtained by optimizing the Gaussian likelihood assuming the null hypothesis is true, i.e., μ=0.

More details about the SHARP test can be found in Supplementary Methods S7. For the purpose of evaluating FPR and statistical power (Methods Section 4.4), we considered two settings: (1) K=5, J=60 and (2) =10, J=30. Here, J was chosen to ensure convergence of the SHARP test.

## Supplementary Material

Supplement 1

## Data Availability

Use of de-identified data from UKB datasets is approved by the National University of Singapore (NUS) Institutional Review Board (IRB). This study used publicly available data from the UK Biobank (https://www.ukbiobank.ac.uk/), EMNIST (https://www.kaggle.com/datasets/crawford/emnist), Covertype (https://archive.ics.uci.edu/dataset/31/covertype) and KEGG Metabolic Pathway (https://archive.ics.uci.edu/dataset/220/kegg+metabolic+relation+network+directed).

## 4.8 Code availability

Code for this study can be found here (GITHUB_LINK). Co-authors (TZ and HL) reviewed each other’s code before merging into the GitHub repository to reduce the chance of coding errors.
