# Supplementary material for: Widespread use of invalid statistical tests in biomedical machine learning
Source: bioRxiv. 2026 May 22:2026.05.17.724301. Preprint. [Version 2] doi: 10.64898/2026.05.17.724301 (PMC13228388; doi:10.64898/2026.05.17.724301)
Supplement: Supplement 1 [file media-1.docx]

**Widespread use of invalid statistical tests in biomedical machine learning**

# Supplemental Material

This supplemental material consists of Supplemental Methods, Results, Tables and Figures to complement the Methods and Results sections in the main text.

# Supplemental Methods

## Large Language Model (LLM) settings

### Prompt for screening papers

We developed an automated pipeline for systematically evaluating academic papers using a large language model (LLM) to identify and classify methodological characteristics related to statistical inference in predictive modeling studies. The implementation leverages the Claude API (claude-opus-4-1-20250805) to perform structured content analysis on full-text scientific articles. We set LLM temperature at 0 for deterministic outputs. No system-level prompt was employed; all instructions were contained within the user message. The maximum output token limit was set to 15,000. The full prompt is shown below:

You are tasked with evaluating a scientific paper by answering questions Q1 through Q5. Follow these instructions carefully and adhere to the specified output formats.

First, read the full text of the paper:

<paper_text>

{{PAPER_TEXT}}

</paper_text>

You will also use the category list below for Q1, the categories are separated by ";"

<category_list>

{{CATEGORY_LIST}}

</category_list>

Now, answer the following questions in order:

**Q1: Paper Classification**

Classify the paper into one of the categories from the category list based on its main content and purpose. Select the most appropriate category that best represents the paper's main goal, method, or contribution. If no category is clearly applicable, answer "OTHERS".

Output your answer in this format:

<answer>

Q1: [The most appropriate category/OTHERS]

Evidence_or_Reason: [One to three sentences justification]

</answer>

**Q2: Method Comparison Evaluation**

Evaluate whether the paper compares at least two distinct alternatives, where each alternative can represent a trainable method or pipeline that undergoes model training and evaluation on data.

Alternatives can differ in algorithms or architectures, hyperparameter configurations, training strategies, feature sets/biomarkers, data modalities/preprocessing pipelines, or can be model selection procedures applied to the development dataset. The comparison must be supported by reported metrics computed directly from predictions against ground truth (e.g., accuracy, AUC, MSE, Pearson correlation).

Answer "Yes" if the condition is met. Answer "No" if the condition is not met.

If your answer is "Yes", provide one to five verbatim sentences from the paper that directly support this.

If your answer is "No", provide a short reasoning explaining why the condition is not met.

Output your answer in this format:

<answer>

Q2: [Yes/No]

Evidence_or_Reason: [One to five sentences justification or verbatim quotes]

</answer>

If you answered "Yes" to Q2, create an internal scratchpad (do not output it) containing all verbatim sentences or short passages in the paper that report quantitative comparisons between methods. You will use this scratchpad for the remaining questions.

**Q3: Statistical Inference Count**

Only evaluate Q3 if Q2 = "Yes". Otherwise, answer "N/A".

Re-read your internal scratchpad from Q2 and the full paper. Identify every instance where the paper performs a statistical inference in a quantitative comparison by reporting either a p-value or a confidence interval. Include cases where p-values or confidence intervals are reported during model selection in the development dataset.

Count the total number of statistical test instances found.

Output your answer in this format:

<answer>

Q3: [Integer/N/A]

</answer>

If your answer to Q3 is greater than 0, create a new internal scratchpad (do not output it) to include the verbatim sentences documenting each statistical test and confidence interval, prefixed with index numbers (1, 2, 3, …).

**Q4: Statistical Test Details**

Only evaluate Q4 if Q3 returns a number greater than zero. Otherwise, answer "N/A".

For each indexed passage in your internal scratchpad created in Q3 that documents a statistical test or confidence interval, provide:

1. Test name: the exact name of the statistical test or interval the paper used (e.g., DeLong test, bootstrap confidence interval, paired t-test, 95% CI)

2. Data description:

a. What two alternatives are compared

b. What prediction performance metric(s) entered the test or confidence interval (e.g., AUCs, accuracies, MSE values)

c. How those performance data were obtained from model training and evaluation (train/test splits, cross-validation folds, bootstrap or permutation resampling, etc.)

Answer in a numbered list corresponding to each indexed statistical test passage from Q3.

Output your answer in this format, you don't need to include the evidence in your answer for Q4:

<answer>

Q4:

1. [Test name]

a: [alternatives compared]

b: [performance metrics used]

c: [how performance data were obtained]

2. [Test name]

a: [alternatives compared]

b: [performance metrics used]

c: [how performance data were obtained]

...

</answer>

After answering Q4, update your internal scratchpad by adding to each indexed test the verbatim sentences that support: (1) the test name, and (2) the description of the performance data used and how those data were obtained.

**Q5: Data Classification**

Only evaluate Q5 if Q3 returns a number greater than zero. Otherwise, answer "N/A".

For each indexed statistical test, classify the values that entered the test or built the confidence intervals into one of:

a. Cross-validation folds - every value is derived from a "resampling unit", which may correspond to: (i) one of the K partitions in K-fold cross-validation, (ii) the test set from one of the K repeated random train-test splits, (iii) one of the N averages obtained from N repetitions of K-fold cross-validation

b. Other / Unclear

Follow these rules:

1. Cross-validation includes K-fold CV, Monte-Carlo CV, Leave-one-out CV, and leave-one-dataset-out CV

2. Apply categories in order: if (a) is satisfied, do not consider (b)

3. Always provide your reasoning after your classification

Output your answer in this format:

<answer>

Q5 Answer:

1. [a/b]

Reason: [your justification]

2. [a/b]

Reason: [your justification]

...

</answer>

If your answer is "a" for any test, add to that indexed test in your scratchpad the verbatim sentences focusing on how the cross-validations are carried out for this test.

**Final Output**

After completing all questions, output your final scratchpad:

<final_scratchpad>

[Your complete scratchpad content with all indexed statistical tests and supporting verbatim sentences]

</final_scratchpad>

**General Rules:**

1. For Q4, always quote verbatim sentences describing the data that entered the test, not just the test name

2. Preserve the exact wording from the paper for all quoted text

3. Do not include any information that cannot be traced back directly to the paper text

4. Follow the conditional logic: Q3-Q5 depend on your answers to previous questions

5. Use the exact output formats specified for each question

### Prompt for evaluating journal rigor

We implemented an automated policy-auditing pipeline using the OpenAI API (gpt-5-2025-08-07) with integrated web-search capability. The model was configured with high reasoning effort, a temperature of 0.2, and a maximum output token limit of 20,000 to prevent truncation. The built-in web_search tool was enabled to allow retrieval of authoritative first-party journal webpages. All task instructions and scoring criteria were provided within the user prompt. The full prompt is shown below:

You are auditing journal policies for: {journal}.

Task:

1) For each criterion below, find the most authoritative public policy page(s) (Instructions for Authors, Editorial Policies, Submission Checklist, Reporting Guidelines).

2) Give exact quotes from webpages of the relevant policy concisely (maximum 3 sentences). If the criterion is not mentioned, say so.

3) Assign a score (0=not mentioned/none, 1=encouraged/optional, 2=required/mandatory).

4) Provide the 1 or 2 best source URLs as evidence for each criterion (prefer first-party journal/publisher pages).

Criteria:

{json.dumps(CRITERIA, ensure_ascii=False, indent=2)}

Description of criteria:

- Code availability policy: Whether the journal encourages or requires authors to share analysis code or model scripts.

- Data availability policy: Whether the journal encourages or requires sharing of underlying datasets or processed data.

- Availability statement requirement: Whether a formal data or code availability statement is encouraged or required in the manuscript.

- Code required for peer review: Whether the journal encourages or requires authors to provide the custom code or scripts used for analysis to editors and reviewers during peer review.

- Statistical test review policy: Whether review of statistical tests is an optional or mandatory part of the editorial or peer review process.

- Reporting standards or checklist: Whether the journal encourages or mandates adherence to recognized methodological reporting standards (e.g., TRIPOD-AI, CONSORT-AI, PRISMA).

Return STRICT JSON matching the provided schema.

Scoring must be conservative; if unclear, give 1 (encouraged) not 2.

Schema:

{json.dumps(schema, ensure_ascii=False)}

### Prompt for extracting study-level code and data availability

We implemented an automated pipeline using the Anthropic API (claude-opus-4-1-20250805) to classify whether each study reported data and/or code availability. The model was configured with a temperature of 0 to ensure deterministic outputs and a maximum output token limit of 15,000 to prevent truncation. No system-level prompt was used; all instructions were contained within the user message. The full prompt is shown below:

You are an expert research auditor evaluating whether a scientific paper shares its data or code publicly.

Below is the full text of a paper (as scraped from PubMed Central).

Please carefully read it and rate the paper according to the criteria described below.

---

<paper text begins here>

{{PAPER_TEXT}}

<paper text ends here>

---

### Your task:

For each criterion, assign a numerical score, provide a one-sentence justification.

If the score is 1, include a short direct quote (1–2 sentences or a few phrases) from the paper text that supports your judgment.

---

#### Scoring guidelines

**1. Data availability (0-1):**

- 0 = No mention of data availability, or data not publicly available.

- 1 = Data partially available or available upon request or publicly available in a repository (e.g., Zenodo, Dryad, GEO, OSF) with a clear link or accession number.

**2. Code availability (0-1):**

- 0 = No mention of code availability or code not available.

- 1 = Code partially available or available upon request or code is publicly available in a repository (e.g., GitHub, Zenodo, OSF) with a clear link.

---

### Output format (JSON only):

{

"data_availability": {

"score": <0-1>,

"justification": "<one-sentence summary of reasoning>",

"quote": "<short direct quote from paper text, empty string if score=0>",

},

"code_availability": {

"score": <0-1>,

"justification": "<one-sentence summary of reasoning>",

"quote": "<short direct quote from paper text, empty string if score=0>",

}

}

## Confidence interval calculation for false positive rate

Methods Section 4.4 describes the simulations to estimate the false positive rate (FPR) of various statistical tests across *m* independent scenarios. To compute confidence intervals for the FPR, we treated the FPR as a binomial proportion, representing the number of significant detections (*p* < 0.05) out of *m* independent tests.

Since binomial proportions, especially at the extremes or with limited sample sizes, often deviate from the normal approximation, we used the **Wilson score interval with continuity correction**, as recommended by a previous study (Newcombe, 1998). Given a total of *m* independent realizations and *n_s_* observed rejections of the null hypothesis, the sample proportion is $\hat{p}=\frac{n_{s}}{m}$. For a 95% confidence level, the critical value from the standard normal distribution is $z_{.025}=1.96$. The continuity-corrected Wilson interval was then computed as (Newcombe, 1998):

$w_{cc}^{-}=\max\left\{ 0,\frac{2m\hat{p}+z_{.025}^{2}-\left[ z_{.025}\sqrt{z_{.025}^{2}-\frac{1}{m}+4m\hat{p}\left( 1-\hat{p} \right)+\left( 4\hat{p}-2 \right)}+1 \right]}{2\left( m+z_{.025}^{2} \right)} \right\}$ (S2.1)

$w_{cc}^{+}=\min\left\{ 1,\frac{2m\hat{p}+z_{.025}^{2}+\left[ z_{.025}\sqrt{z_{.025}^{2}-\frac{1}{m}+4m\hat{p}\left( 1-\hat{p} \right)-\left( 4\hat{p}-2 \right)}+1 \right]}{2\left( m+z_{.025}^{2} \right)} \right\}$ (S2.2)

This method provides more accurate interval bounds than the standard Wald interval, particularly when the proportion is near 0 or 1, or when *m* is small (Newcombe, 1998).

## Dependence in cross-validation, naïve t-test & corrected t-test

In this section we develop a model for performance difference under different cross-validation schemes. First, we assume that two models are being compared by performing a single run of K-fold cross-validation. The data split for the K-fold cross-validation is identical for both models, so there is fold-level correspondence between the two models. In each of *K* iterations we train the two models on *K*−1 folds and test the two resulting models on the remaining fold. Therefore, each test fold yields a performance metric difference, and any pair of performance differences is correlated due to overlap of training data (for $K>2$) and overlap between testing data in one fold and the training data for a different fold. Throughout, we denote $\boldsymbol{D}$ as the length-$J$vector of performance metric differences; here $J=K$.

Now consider Monte Carlo cross-validation, where the dataset is repeatedly divided into training and test sets $J$ times, again leading to $J$ fold-level differences. In this case, there is overlap in both training and testing data between folds. The performance metric differences $\boldsymbol{D}$ are equally correlated, just like a single run of K-fold cross-validation.

Assuming the performance metric differences follows a Gaussian distribution, we can write

$\boldsymbol{D}\sim N\left( \mu\mathbf{1},\boldsymbol{\Sigma} \right)$ (S3.1)

where $\mu$ is a scalar representing the true population average performance metric difference. $\boldsymbol{1}$ is a length-$J$ column vector of ones, and

$\boldsymbol{\Sigma}=\sigma^{2}\left[ \begin{matrix} 1 & \rho& \rho\\ \rho& \ddots& \rho\\ \rho& \rho& 1 \end{matrix} \right]$ (S3.2)

is the $J\times J$ covariance matrix, $\sigma^{2}$is the variance of a given fold-level statistic and $\rho$ is the correlation between the fold-level statistics. The diagonals of $\boldsymbol{\Sigma}$ are $\sigma^{2}$, while all off-diagonal entries are $\sigma^{2}\rho$. By symmetry of the cross-validation procedure, $\boldsymbol{\Sigma}$ has a compound symmetric correlation structure, where each fold is correlated to a different fold with correlation $\rho$.

To perform a statistical test of whether the mean of a Gaussian variable is statistically different from zero, we need an estimate of the mean and the variance of the estimator of the mean. In the current cross-validation setup, the mean accuracy difference is $\bar{D}=\frac{1}{J}\boldsymbol{1}^{\top}\boldsymbol{D}$, which is an unbiased estimator of $\mu$, i.e., $E\left( \bar{D} \right)=\mu.$

The variance of the estimator $\bar{D}$ is not easy to estimate (Nadeau & Bengio, 2003). Based on Equations S3.1 and S3.2 (above), the variance of the estimator $\bar{D}$ is given by

$\mathrm{Var}\left( \bar{D} \right)=\frac{1}{J^{2}}\mathbf{1}^{\top}\boldsymbol{\Sigma1}=\frac{\sigma^{2}}{J^{2}}\left( J+J\left( J-1 \right)\rho\right)=\sigma^{2}\left( \frac{1}{J}+\frac{J-1}{J}\rho\right).$ (S3.3)

However, we have two unknown variance parameters $\sigma^{2}$ and $\rho$ and a single sufficient statistic for the variance, the sample variance $S^{2}$. The sample variance of $\boldsymbol{D}$can be written

$S^{2}=\frac{1}{J-1}\sum_{j} \left( D_{j}-\bar{D} \right)^{2}=\frac{1}{J-1}\boldsymbol{D}^{\top}\left( \mathbf{I}-\frac{1}{J}\mathbf{J} \right)\boldsymbol{D},$ (S3.4)

where $D_{j}$ is the *j*-th entry of $\boldsymbol{D}$, $\mathbf{I}-\frac{1}{J}\mathbf{J}$ is the centering matrix, $\mathbf{I}$ is the identity and $\mathbf{J}$ is a square matrix of 1’s. Unfortunately, $S^{2}$ is a biased estimator of $\sigma^{2}$:

$\text{E}\left( S^{2} \right)=\frac{1}{J-1}\text{tr}\left( \boldsymbol{\Sigma}\left( \mathbf{I}-\frac{1}{J}\mathbf{J} \right) \right)$

$=\frac{1}{J-1}\left( J\sigma^{2}-\frac{1}{J}\left( J\sigma^{2}\left( 1+\left( J-1 \right)\rho\right) \right) \right)$

$=\sigma^{2}\left( 1-\rho\right)$ (S3.5)

where we have used the following result: for a given random vector $\boldsymbol{x}$ with mean $\boldsymbol{\mu}$ and covariance $\boldsymbol{\Sigma}$ and a constant matrix $\mathbf{A}$, $\text{E}\left( \boldsymbol{x}^{\top}\mathbf{A}\boldsymbol{x} \right)=\text{tr}\left( \boldsymbol{A\Sigma} \right)+\boldsymbol{\mu}^{\top}\mathbf{A}\boldsymbol{\mu}$.

Since we expect the correlation $\rho$ to be positive between cross-validation folds, $1-\rho$ is smaller than one and so the sample variance $S^{2}$ underestimates $\sigma^{2}$. The relationship between $E(S^{2})$ and $\mathrm{Var}\left( \bar{D} \right)$ is thus given by (Nadeau & Bengio, 2003):

$\mathrm{Var}\left( \bar{D} \right)=\left( \frac{1}{J}+\frac{\rho}{1-\rho} \right)\text{E}\left( S^{2} \right)$ (S3.6)

$= \frac{1}{J}\text{E}\left( S^{2} \right)+ \frac{\rho}{1-\rho}\text{E}\left( S^{2} \right)$ (S3.7)

Since the correlation $\rho$ is expected to be positive between cross-validation folds, the second term in Equation S3.7 is positive. A conventional paired t-test applied in this setting is called a resampled paired t-test, and has the form:

$T=\frac{\bar{D}}{\sqrt{\frac{1}{J}S^{2}}}$. (S3.8)

Therefore, when studies directly apply the paired-sample t-test to compare model performance, they are using only the first term in Equation S3.7, which is thus an underestimate of the variance of the mean estimator $\mathrm{Var}\left( \bar{D} \right)$. Consequently, the resampled paired-sample t-test is expected to yield an inflated false positive rate.

Equation S3.6 provides the form of a bias correction needed to be used in the corrected resampled t-test (Nadeau & Bengio, 2003), which is to multiply the sample variance $S^{2}$ by $\frac{1}{J}+\frac{\rho}{1-\rho}$ (instead of $\frac{1}{J}$ as in Equation S3.8). Nadeau and Bengio assume that the correlation between folds $\rho=$ $\frac{N_{2}}{N_{1}+N_{2}}$ , where $N_{1}$​ and $N_{2}$ are the number of training and test samples, respectively. Therefore, $\frac{\rho}{1-\rho}$ becomes $\frac{N_{2}}{N_{1}}$, leading to the following statistic:

$T=\frac{\bar{D}}{\sqrt{\left( \frac{1}{J}+\frac{N_{2}}{N_{1}} \right)S^{2}}},$ (S3.9)

which Nadeau & Bengio (2003) call the “corrected resampled t-test”.

Many studies perform repeated K-fold cross-validation, where the K-fold procedure is repeated $R$ times, resulting in $J = K \times R$ fold-level differences*.* While the corrected paired t-test has also been applied to repeated K-fold cross-validation (Bouckaert & Frank, 2004), the theoretical justification is weaker. The repeated K-fold cross-validation produces a $\boldsymbol{D}$ with a nested correlation structure, where the correlation between folds within a particular instance of K-fold cross-validation is different from the correlation between folds from two different repeats of K-fold cross-validation. Nevertheless, in practice we find there is no difference in FPR between the application of the corrected resampled t-test in Monte Carlo cross-validation and repeated K-fold cross-validation (Fig. S5).

The strongest assumption of the corrected resampled t-test is that the correlation between folds $\rho$ is $\frac{N_{2}}{N_{1}+N_{2}}$. In reality, the correlation magnitude likely depends on the complex interaction between the machine learning algorithm and the dataset being analyzed. As shown in the main results, the corrected resampled t-test can have a slightly elevated FPR (Fig. 6), and worse power than the SHARP test (Fig. 7).

## Conservativeness of 5×2 t-test and 5×2 F-test

Our results suggest that both 5×2 t-test (Dietterich, 1998) and 5×2 F-test (Alpaydin, 1999) reliably control FPR (Fig. 6 & Fig. S5) and both tests had lower statistical power than the SHARP test (Fig. 7 & Fig. S7). In this section, we provide some insights on why the 5×2 tests are more conservative than the resampled paired t-test.

Recall that the 5×2 t-test tests involve 5 repetitions of 2-fold cross-validation. For each repetition $r\in\left\{ 1,2,3,4,5 \right\}$, the data is randomly split into two halves A and B. Both models are trained on set A and evaluated on test set B, resulting in performance difference $D_{Br}$. Both models are also trained on set B and evaluated on test set A, resulting in performance difference $D_{Ar}$.

### 5×2 paired t-test

The 5×2 t-test utilizes the following statistic (Dietterich, 1998):

$T=\frac{D_{A1}}{\sqrt{\frac{1}{5}\sum_{r=1}^{5} S_{r}^{2}}}$ (S4.1)

where $S_{r}^{2}=\left( D_{Ar}-D_{Br} \right)^{2}/2$. The statistic is assumed to follow a Student’s t distribution with 5 degrees of freedom, from which a p-value can be computed.

Following the notation in Supplementary Methods S3, we note that $E\left( D_{Ar} \right)=E\left( D_{Br} \right)=\mu$, the fold-level statistics have a variance of $\sigma^{2}$ and the correlation between fold-level statistics within a single run of two-fold cross-validation is $\rho$. For convenience, we can write $D_{Ar}=\mu+\epsilon_{Ar}$, and $D_{Br}=\mu+\epsilon_{Br}$, where $\epsilon_{Ar}$ and $\epsilon_{Br}$ are zero mean random variables with variance $\sigma^{2}$. Furthermore, ${\mathrm{Cov}(\epsilon}_{Ar}, \epsilon_{Br})=\sigma^{2}\rho$. Note that there is also a correlation $\rho'$ across the 5 repeats of the 2-fold cross-validation, but this $\rho'$ is not necessary for the analysis below.

The numerator $D_{A1}$ has mean $\mu$ and variance $\sigma^{2}$. Ideally, we would like the denominator $\frac{1}{5}\sum_{i=1}^{5} S_{r}^{2}$ to be an unbiased estimate of the variance of the numerator. While Dietterich assumes that the $D_{Ar}$ and $D_{Br}$ are independent, we find that

$E\left( \frac{1}{5}\sum_{r=1}^{5} S_{r}^{2} \right)=\frac{1}{5}\sum_{r=1}^{5} E\left( \frac{\left( D_{Ar}-D_{Br} \right)^{2}}{2} \right)$

$=\frac{1}{5}\sum_{r=1}^{5} E\left( \frac{\left( \epsilon_{Ar}-\epsilon_{Br} \right)^{2}}{2} \right)$

$=\frac{1}{5}\sum_{r=1}^{5} \frac{1}{2}\left( \sigma^{2}+\sigma^{2}-2\rho\sigma^{2} \right)$

$= \sigma^{2}\left( 1-\rho\right)$. (S4.2)

Therefore, ideally the 5×2 t-statistic should be multiplied by$\sqrt{1-\rho}$ (but $\rho$ is unknown). When the 5×2 t-test was first introduced, it was demonstrated that correlation $\rho$ between two folds can be positive, which might cause the test to be liberal (Dietterich, 1998).

Let us contrast this with the paired t-test applied to a single run of K-fold cross-validation. Based on Supplementary Methods S3, the correction factor required for the paired t-test is $\sqrt{\frac{1}{K}/\left( \frac{1}{K}+\frac{\rho}{1-\rho} \right)}=\sqrt{\frac{1-\rho}{1+\left( K-1 \right)\rho}}$ (based on Equation S3.6), which is a much stronger correction factor than the 5×2 test ($\sqrt{1-\rho}$). Therefore, the 5×2 t-test is more conservative than the paired t-test applied to a single run of K-fold cross-validation.

However, we note that although the 5×2 t-test is theoretically slightly liberal, in practice, the 5×2 t-test reliably controls FPR (Fig. 6), but its power is significantly worse than the SHARP test (Fig. 7).

### 5×2 paired F-test

To address the inefficiency of the 5×2 t-test, Alpaydin (Alpaydin, 1999) proposed the 5×2 paired F-test, which uses the following statistic:

$F=\frac{\sum_{r=1}^{5} \left( D_{Ar}^{2}+D_{Br}^{2} \right)}{2\sum_{r=1}^{5} S_{r}^{2}}$ (S4.3)

where $S_{r}^{2}=\left( D_{Ar}-D_{Br} \right)^{2}/2$ (same as the 5×2 t-test). The statistic is assumed to follow the F-distribution with 10 and 5 degrees of freedom in the numerator and denominator, from which a p-value can be defined.

We can check the validity of this F-test by comparing the expectation of numerator to that of denominator under the null hypothesis. Ideally, these two expectations should be equal. Under null hypothesis, $\mu=0$, we have:

$E\left( \sum_{r=1}^{5} \left( D_{Ar}^{2}+D_{Br}^{2} \right) \right)= 10\sigma^{2}$ (S4.4)

$E\left( 2\sum_{r=1}^{5} S_{r}^{2} \right)= 10\sigma^{2}(1-\rho)$ (S4.5)

The expectation of the denominator is smaller than the expectation of the numerator, suggesting that the 5×2 F-test might be slightly liberal, similar to the 5×2 t-test. However, in practice, the 5×2 F-test reliably controls FPR (Fig. S5), but its power is significantly worse than the SHARP test (Fig. S7).

## Conservativeness of the empirical test of differences

As explained in Methods Section 4.5.7, given a vector ***D*** of *J* fold-level differences between two models, the empirical test of differences constructs an empirical histogram from the entries in the vector ***D*** and computes the fraction of entries in which the (overall) worse model outperforms the (overall) better model. To derive a two-sided test p-value, the empirical test of differences then multiplies the fraction by two.

We explore why the empirical test of differences is conservative through the following framework. Assume the *J* fold-level differences follow a Gaussian distribution. Given a single instance of K-fold cross-validation or Monte Carlo cross validation, the empirical distribution of the accuracy differences in $\boldsymbol{D}$ is approximately Gaussian, centered at $\bar{D}$ with spread measured by $S^{2}$, where $E\left( S^{2} \right)=\sigma^{2}\left( 1-\rho\right)$ (Equation S3.5). The sample variance reflects only the variability within vector $\boldsymbol{D}$ and not the marginal variance $\sigma^{2}$. Without loss of generality, suppose $\bar{D}$ is negative, then the empirical test of differences is essentially computing 2× the area of the right positive tail of this Gaussian distribution.

On the other hand, we note that an oracle-based paired t-test that has knowledge of $\rho$ can be thought of as computing 2× the area of the right positive tail (again, assuming negative $\bar{D})$ of a Gaussian distribution with mean $\bar{D}$ and variance given by $\mathrm{Var}\left( \bar{D} \right)=\left( \frac{1}{J}+\frac{\rho}{1-\rho} \right)\text{E}\left( S^{2} \right)$ (Equation S3.6).

Considering these two distributions that generate p-values, both centered at $\bar{D}$ but with different variances, illustrates why the empirical test of differences is less powerful. The empirical test of differences’ distribution has variance expected to be $\sigma^{2}\left( 1-\rho\right)$, while the oracle paired t-test has variance that differs by a factor of $\left( \frac{1}{J}+\frac{\rho}{1-\rho} \right)$. Therefore, if $\left( \frac{1}{J}+\frac{\rho}{1-\rho} \right)<1$, then the empirical test is likely to be more conservative than the oracle paired t-test. This condition can be re-written as $\rho<\frac{J-1}{2J-1}$, so if J is very large, then this is equivalent to $\rho$ < 0.5. Therefore, if $\rho$ is much smaller than 0.5, then the empirical test of differences will be significantly less powerful than an oracle paired t-test.

Indeed, we find that the empirical test of differences reliably controls the FPR (Fig. 6), while having the worst power (Fig. 7), suggesting that $\rho$ is positive, but much smaller than 0.5.

## Two-algorithm simulation scheme

Our main results utilized a simulation scheme for evaluating FPR, in which the same algorithm was trained on two noisy versions of the same dataset. Similarly, statistical power was evaluated by training the same algorithm on a clean dataset and a noisy version of the same dataset. As a supplementary analysis, we considered the two-algorithm simulation scheme (Dietterich, 1998; Nadeau & Bengio, 2003). The drawback of the two-algorithm simulation scheme is that we have to assume the true difference between the two algorithms is known, in order to evaluate the FPR. However, in practice, the results of the two-algorithm simulations were very similar to the noisy-model simulations from the main results.

### Algorithm Selection

We considered four datasets: EMNIST, Covertype, KEGG Metabolic, and UK Biobank. For each dataset, we selected two algorithms to compare. Similar to the noisy-model simulation, we used the AutoML package PyCaret (Ali, 2020) to explore a set of algorithms available in the scikit-learn package with default hyperparameters (Pedregosa et al., 2011). Because the simulations were highly computationally expensive, similar to the main results, we prioritized algorithms that were fast to run. We also prioritized pairs of algorithms that exhibited a large performance difference.

More specifically, we randomly selected 20,000, 20,000, 10,000, and 5,000 samples from EMNIST, Covertype, KEGG Metabolic, and UK Biobank datasets, respectively. Each sampled dataset was then partitioned into disjoint subsets of 1,000 samples. For each candidate algorithm, we conducted a 5-fold cross-validation on each subset. The performance metric of interest (e.g., classification accuracy) was averaged across five folds and recorded. This procedure yielded 20, 20, 10, and 5 averaged performance metrics for EMNIST, Covertype, KEGG Metabolic, and UK Biobank, respectively, which were then further averaged to obtain a single performance estimate per algorithm on each dataset. Additionally, we recorded and averaged the runtime required to complete one 5-fold cross-validation for each algorithm.

Based on the selection criteria outlined above, we chose the following algorithm pairs:

1. EMNIST: Extra Tree Classifier and Linear Discriminant Analysis
2. Covertype: Random Forest Classifier and Support Vector Machine with Linear Kernel
3. KEGG Metabolic: Extra Tree Regressor and K-Nearest Neighbors Regressor
4. UK Biobank: Extra Tree Regressor and K-Nearest Neighbors Regressor

For each dataset, the algorithm listed first in the pair achieved numerically better prediction performance. Furthermore, the data samples used to select the two algorithms were excluded for subsequent analyses to avoid biasing the FPR and power simulations in the following sections. We will first explain how to evaluate power and then discuss how FPR can be obtained.

### Simulation procedure for statistical power

Consistent with the main results, we conducted analyses on four datasets — EMNIST, UKB, Covertype, and KEGG metabolic pathway — using four sample sizes: *N* = 100, 500, 1000, and 2000. For EMNIST, *N* = 100 was excluded because with 10 digit classes, it was not possible to guarantee at least one instance of each class in the test set under the SHARP test, which required a split-half step within the cross-validation scheme (see Methods Section 4.6). For UKB, *N* = 2,000 was excluded because the maximum number of non-overlapping sampled datasets that could be drawn from UKB at this sample size was fewer than 20, which we considered too few to yield stable estimates. Therefore, across the four datasets and sample sizes, there were 14 scenarios.

For each sample size *N*, we drew *m* non-overlapping sampled datasets of size *N* from the full dataset. The value of *m* was set to 100 when feasible; otherwise, it was set to the maximum number of non-overlapping sampled datasets that could be drawn. For each of *m* sampled datasets and each of the two algorithms associated with the dataset (previous section), we performed either repeated K-fold cross-validation or Monte Carlo cross-validation. In classification tasks, stratified splitting was used to preserve label proportions across folds.

All data splits were kept identical across both algorithms, so that we ended up with a vector $\boldsymbol{D}$of $J$ performance differences. In the case of the SHARP test, each of the *m* non-overlapping datasets was first divided into two non-overlapping halves, and cross-validation was performed within each half (see Methods Section 4.6 for details). We note that SHARP operates on a vector $\boldsymbol{D}$ of $2J$ performance differences.

Finally, the vector $\boldsymbol{D}$ of differences between the two algorithms was used in different statistical tests with the null hypothesis $\mu=0$, where $\mu$ is the true expected difference between the two trained models. Since the two algorithms have been selected so that their prediction performance was different from each other, a well-calibrated test should reject the null hypothesis. Statistical power was estimated as the fraction of *m* sampled datasets in which the null hypothesis was rejected at a significance threshold of 0.05. We then averaged the power across 14 scenarios, resulting in an average power for each test.

### Simulation procedure for false positive rate (FPR)

To estimate the FPR, the simulation procedure is the same as evaluating statistical power. However, since the two algorithms were selected so that their prediction performance was different from each other, the FPR was more difficult to estimate since we expected the null hypothesis $\mu=0$ to be rejected.

If we knew the true difference between the two algorithms $\mu_{\mathrm{true}}$, we could instead evaluate the null hypothesis $\mu=\mu_{\mathrm{true}}$, in which case rejection of the null hypothesis constituted a false positive. However, we note that $\mu_{\mathrm{true}}$ was unknown. The value of $\mu_{\mathrm{true}}$ was also likely to be different across sample sizes and datasets.

Therefore, for each of the 14 scenarios, we estimated $\mu_{\mathrm{true}}$, by averaging the entries of the vector $\boldsymbol{D}$ to obtain $\bar{D}$, and then averaged $\bar{D}$ across the *m* non-overlapping datasets, yielding $\hat{\mu}_{\mathrm{true}}$. For each statistical test, we then evaluated the null hypothesis $\mu=\hat{\mu}_{\mathrm{true}}$. Rejection of the null hypothesis constituted a false positive.

FPR was estimated as the fraction of *m* sampled datasets in which the null hypothesis was rejected at a significance threshold of 0.05. The 95% confidence interval for the FPR was computed using the Wilson score interval with continuity correction (Newcombe, 1998); the formula is provided in Supplementary Methods S2. Therefore, for each statistical test, we ended up with 14 FPRs and 95% confidence intervals for the FPRs.

### Confidence interval of prediction performance differences

To estimate the confidence interval of performance difference between the two algorithms, we followed a similar procedure as the noisy-model simulations (Section 4.4.3). Recall that we had 14 scenarios in the two-algorithm simulation scheme. For each scenario and each of *m* non-overlapping datasets, we found the range $[\mu_{L},\mu_{U}]$ (i.e., confidence interval), such that each null hypothesis $H_{0}:\mu=\mu_{0}$, $\mu_{0}\in[\mu_{L},\mu_{U}]$, could not be rejected, i.e., $p\geq0.05$. Since we have *m* non-overlapping datasets, we had *m* confidence intervals in total. We then counted the fraction of times $\hat{\mu}_{\mathrm{true}}$ (Supplementary Methods S6.3) fell within the confidence interval, which we referred to as the overall coverage rate. For a well-calibrated 95% confidence interval, $\mu_{\mathrm{true}}$ should fall into the confidence interval 95% of the times.

## SHARP tests (full details)

### Covariance structure of Split-HAlf RePeated (SHARP) cross-validation

Our goal is to compare two machine learning models. Instead of the traditional cross-validation scheme, we randomly divide the dataset into two disjoint halves A and B. For each machine learning model, we then perform K-fold cross-validation in subsets A and B separately. The data split for the K-fold cross-validation is identical for both models, so there is fold-level correspondence between the two models. We then averaged the results across the K-fold cross-validation, resulting in model performance differences $D_{A1}$ and $D_{B1}$ respectively. This process is repeated *J* times, resulting in two vectors $\boldsymbol{D}_{A}=\left[ D_{A1},\ldots,D_{AJ} \right]^{\top}$ and $\boldsymbol{D}_{B}=\left[ D_{B1},\ldots,D_{BJ} \right]^{\top}$.

Suppose there are model hyperparameters to be estimated, then the K-fold cross-validation can be modified to become a nested cross-validation where hyperparameters are estimated in the inner cross-validation loop. Alternatively, we can perform Monte Carlo cross-validation, where the subset A (or B) is repeatedly divided into training, validation and test sets. Regardless of the type of cross-validation scheme, we end up with two vectors $\boldsymbol{D}_{A}=\left[ D_{A1},\ldots,D_{AJ} \right]^{\top}$ and $\boldsymbol{D}_{B}=\left[ D_{B1},\ldots,D_{BJ} \right]^{\top}$.

Let $\boldsymbol{D}=\left[ \boldsymbol{D}_{A}^{\top},\boldsymbol{D}_{B}^{\top} \right]^{\top}=\left[ D_{A1},\ldots,D_{AJ}, D_{B1},\ldots,D_{BJ} \right]^{\top}$, so $\boldsymbol{D}$ is a column vector of length $2J$. We denote $\mathrm{Var}\left( D_{Aj} \right)=Var\left( D_{Bj} \right)=\sigma^{2}$. Because of the disjoint subsets A and B, for any iteration *j*, $\mathrm{Corr}\left( D_{Aj},D_{Bj} \right)=0$. On the other hand, for two different iterations *j* and *k*, $\mathrm{Corr}\left( D_{Aj},D_{Ak} \right)=Corr\left( D_{Bj},D_{Bk} \right)=Corr\left( D_{Aj},D_{Bk} \right)=\rho$. Therefore, the covariance matrix of $\boldsymbol{D}$ can be written as

$\boldsymbol{\Sigma}\left( \sigma^{2},\rho\right)=\sigma^{2}\left[ \begin{matrix} \boldsymbol{M} & \boldsymbol{C} \\ \boldsymbol{C} & \boldsymbol{M} \end{matrix} \right]$ (S7.1)

where $\boldsymbol{M}\in\mathbb{R}^{J\times J}$ has ones on the diagonal and *ρ* in all off-diagonal entries, and $\boldsymbol{C}\in\mathbb{R}^{J\times J}$ has zeros on the diagonal and *ρ* in all off-diagonal entries:

$M_{jk}=\left\{ \begin{aligned} 1,j=k \\ \rho,j\neq k \end{aligned} \right. \mathrm{or} \boldsymbol{M}=\left[ \begin{matrix} 1 & \rho& \rho\\ \rho& \ddots& \rho\\ \rho& \rho& 1 \end{matrix} \right]$ (S7.2)

and

$C_{jk}=\left\{ \begin{aligned} 0,j=k \\ \rho,j\neq k \end{aligned} \right.\mathrm{or} \boldsymbol{C}=\left[ \begin{matrix} 0 & \rho& \rho\\ \rho& \ddots& \rho\\ \rho& \rho& 0 \end{matrix} \right]$ (S7.3)

### GLS estimator of *μ* reduces to the sample mean

We assume that the vector $\boldsymbol{D}$ (of length 2J) follows a Gaussian distribution:

$\boldsymbol{D}\sim N(\mu\boldsymbol{1,}\boldsymbol{\Sigma}\boldsymbol{)}$ (S7.4)

The generalized least-squares (and maximum-likelihood) estimator for *μ* and the associated variance are as follows (Yan, 2009):

$\hat{\mu}_{GLS}=\left( \mathbf{1}^{\top}\boldsymbol{\Sigma}^{-1}\mathbf{1} \right)^{-1}\mathbf{1}^{\top}\boldsymbol{\Sigma}^{-1}\boldsymbol{D},$ (S7.5)

Because each row of $\boldsymbol{\Sigma}$sums to a constant value, we note that the vector $\boldsymbol{1}$ is an eigenvector of $\boldsymbol{\Sigma}$ (and $\boldsymbol{\Sigma}^{-1}$). Therefore, $\boldsymbol{\Sigma}^{-1}\boldsymbol{1}=\left( 1/c \right)\boldsymbol{1}$ for some scalar c > 0, so

$\left( \boldsymbol{1}^{\top}\boldsymbol{\Sigma}^{-1}\boldsymbol{1} \right)^{-1}\boldsymbol{1}^{\top}\boldsymbol{\Sigma}^{-1}=\frac{1}{2J}\boldsymbol{1}^{\top}\boldsymbol{.}$ (S7.6)

Note that $1/c$ is the sum of any row of $\boldsymbol{\Sigma}^{-1}$, and $c$ is the sum of any row of $\boldsymbol{\Sigma}$. Plugging Equation S7.6 back into Equation S7.5, we get

$\hat{\mu}_{GLS}=\frac{1}{2J}\boldsymbol{1}^{\top}\boldsymbol{D}=\bar{D},$ (S7.7)

thus showing that the GLS and sample mean coincide. We also note that $E\left( \bar{D} \right)=\mu$, and furthermore, we have:

$\mathrm{Var}\left( \bar{D} \right)=Var\left( \frac{1}{2J}\boldsymbol{1}^{\top}\boldsymbol{D} \right)$

$=\frac{1}{4J^{2}}\boldsymbol{1}^{T}\boldsymbol{\Sigma}\boldsymbol{1}$

$=\frac{\sigma^{2}}{4J^{2}}\left[ 2J+4J\left( J-1 \right)\rho\right]$

$=\sigma^{2}\left( \frac{1}{2J}+\frac{J-1}{J}\rho\right),$ (S7.8)

where we used the following result: for a random vector $\boldsymbol{x}$ with covariance matrix $\boldsymbol{\Sigma}$ and constant vector $\boldsymbol{a}$, $\mathrm{Var}\left( \boldsymbol{a}^{T}\boldsymbol{x} \right)=\boldsymbol{a}^{T}\boldsymbol{\Sigma}\boldsymbol{a}$. Therefore, if we can estimate $\sigma^{2}$ and $\rho$, we will be able to estimate $\mathrm{Var}\left( \bar{D} \right)$, and perform a statistical test. In the following sections, we outline different approaches to performing the statistical test.

### Method-of-moments (MoM) estimates and Wald test

In this section, we use the method of moments (MoM) to estimate $\sigma^{2}$ and $\rho$. The Wald test is then used to perform the statistical test. We first compute the mean within subsets A and B.

$\bar{D}_{A}=\frac{1}{J}\sum_{j=1}^{J} D_{Aj},$ (S7.9)

$\bar{D}_{B}=\frac{1}{J}\sum_{j=1}^{J} D_{Bj},$ (S7.10)

$\bar{D} =\frac{\bar{D}_{A}+\bar{D}_{B}}{2}.$ (S7.11)

We then compute sample variances within subsets A and B:

$S_{A}^{2}=\frac{1}{J-1}\sum_{j=1}^{J} \left( D_{Aj}-\bar{D}_{A} \right)^{2}$ (S7.12)

$S_{B}^{2}=\frac{1}{J-1}\sum_{j=1}^{J} \left( D_{Bj}-\bar{D}_{B} \right)^{2}.$ (S7.13)

Similar to Equation S3.5 in Supplementary Methods S3, $E\left( S_{A}^{2} \right)=E\left( S_{B}^{2} \right)=\sigma^{2}\left( 1-\rho\right)$. Let us also define the between-subset sample variance:

$\hat{\sigma}_{\Delta}^{2}=\frac{1}{2J}\sum_{j=1}^{J} \left( D_{Aj}-D_{Bj} \right)^{2}$ (S7.14)

Using Equation S4.2 in the 5×2 t-test (Supplementary Methods S4.1), except that under SHARP cross-validation scheme, $\mathrm{Cov}\left( D_{Aj},D_{Bj} \right)=0$, we get $E\left( \hat{\sigma}_{\Delta}^{2} \right)=\sigma^{2}$. We then use the following MoM estimators of *ρ* and $\sigma^{2}$:

$\hat{\rho}_{\mathrm{MoM}}=\frac{\hat{\sigma}_{\Delta}^{2}-\frac{1}{2}\left( S_{A}^{2}+S_{B}^{2} \right)}{\hat{\sigma}_{\Delta}^{2}},$ (S7.15)

$\hat{\sigma}_{\mathrm{MoM}}^{2}=\hat{\sigma}_{\Delta}^{2}$ (S7.16)

Furthermore, by substituting $\hat{\rho}_{\mathrm{MoM}},\hat{\sigma}_{\mathrm{MoM}}^{2}$ into Equation S7.8, we get $\hat{\mathrm{Var}}\left( \bar{D} \right)=\hat{\sigma}_{\mathrm{MoM}}^{2}\left( \frac{1}{2J}+\frac{J-1}{J}\hat{\rho}_{\mathrm{MoM}} \right)$. Finally, the MoM Wald test uses the following statistic:

$Z_{\mathrm{MoM}}=\frac{\bar{D}}{\sqrt{\hat{\sigma}_{\mathrm{MoM}}^{2}\left( \frac{1}{2J}+\frac{J-1}{J}\hat{\rho}_{\mathrm{MoM}} \right)}}$ (S7.17)

Under the null hypothesis of equal performance between the two algorithms, $Z_{\mathrm{MoM}}$ is approximately distributed as $N\left( 0, 1 \right)$, and the corresponding two-sided p-value is given by $p=2\left( 1-\Phi\left( \left| Z_{\mathrm{MoM}} \right| \right) \right)$, where $\Phi\left( \cdot\right)$ denotes the standard normal cumulative distribution function.

### Maximum likelihood (ML) estimation and Wald test

Recall that we assume $\boldsymbol{D}\sim N\left( \mu\boldsymbol{1},\boldsymbol{\Sigma}\left( \sigma^{2},\rho\right) \right)$. The log likelihood is given by

$\mathcal{l}\left( \mu,\sigma^{2},\rho\right)=-\frac{1}{2}\log\left| \boldsymbol{\Sigma} \right|-\frac{1}{2}\left( \boldsymbol{D}-\mu\boldsymbol{1} \right)^{\top}\boldsymbol{\Sigma}^{-1}\left( \boldsymbol{D}-\mu\boldsymbol{1} \right)+\text{constant}$ (S7.18)

From Supplementary Methods S7.2, the ML estimate of *μ* is equal to $\bar{D}$. The remaining parameters $\sigma^{2}$ and $\rho$ can be estimated by maximizing $\mathcal{l}\left( \bar{D},\sigma^{2},\rho\right)$, yielding $\hat{\sigma}_{\mathrm{ML}}^{2}$ and $\hat{\rho}_{\mathrm{ML}}$. In our implementation, we utilized the “minimize” function in the scipy.optimize package, and initialized the optimization with the method of moments estimates $\hat{\rho}_{\mathrm{MoM}},\hat{\sigma}_{\mathrm{MoM}}^{2}$.

The MLE-based Wald statistic is

$Z_{\mathrm{ML}}=\frac{\bar{D}}{\sqrt{\hat{\sigma}_{\mathrm{ML}}^{2}\left( \frac{1}{2J}+\frac{J-1}{J}\hat{\rho}_{\mathrm{ML}} \right)}}$ (S7.19)

and the corresponding two-sided p-value is given by $p=2\left( 1-\Phi\left( \left| Z_{\mathrm{ML}} \right| \right) \right)$, where $\Phi\left( \cdot\right)$ denotes the standard normal cumulative distribution function.

### Restricted maximum likelihood (ReML) and Wald test

Variance parameters estimated with maximum likelihood are well known to have bias when estimated alongside mean parameters, particularly in small samples (Lehmann & Casella, 1998; Hogg et al., 2013). Building a likelihood based on contrasts that remove the mean parameters while retaining all other information in the data gives estimates with reduced bias, which is known as Restricted Maximum Likelihood (ReML).

In the SHARP cross-validation scheme, recall that we assume $\boldsymbol{D}\sim N(\mu\boldsymbol{1}, \boldsymbol{\Sigma})$. ReML can be implemented by centering the mean of the distribution to zero. We define the following residual-forming matrix

$\boldsymbol{R}=\mathbf{I}_{2J\times2J}-\frac{1}{2J}\mathbf{J}_{2J\boldsymbol{\times}2J}$ (S7.20)

where $\mathbf{I}$ is the identity matrix and $\mathbf{J}$ is the matrix of all ones. To ensure a full-rank transformation for likelihood evaluation, we drop the last row of $\boldsymbol{R}$, resulting in $\boldsymbol{R}^{*}=\left( \boldsymbol{R} \right)_{1:\left( 2J-1 \right),1:2J}$. The transformed residuals have the distribution

$\boldsymbol{R}^{*}\boldsymbol{D}\sim N\left( 0,\boldsymbol{R}^{*}\boldsymbol{\Sigma}\boldsymbol{R}^{*\top} \right)$ (S7.21)

Similar to the previous section (Supplementary Methods S7.4), we can maximize the new likelihood to estimate $\hat{\sigma}_{\mathrm{ReML}}^{2},\hat{\rho}_{\mathrm{ReML}}$, which can be used to compute the corresponding Wald statistic

$Z_{\mathrm{ReML}}=\frac{\bar{D}}{\sqrt{\hat{\sigma}_{\mathrm{ReML}}^{2}\left( \frac{1}{2J}+\frac{J-1}{J}\hat{\rho}_{\mathrm{ReML}} \right)}}$ (S7.22)

The two-sided p-value is given by $p=2\left( 1-\Phi\left( \left| Z_{\mathrm{ReML}} \right| \right) \right)$, where $\Phi\left( \cdot\right)$ denotes the standard normal cumulative distribution function.

### Score test (used in the main Methods)

We also consider the use of likelihood-based score test, which evaluates the slope of the log-likelihood (known as the score) with respect to $\mu$ at $\mu=0$ (under our null hypothesis), with nuisance parameters $(\sigma^{2},\rho)$ estimated under the null.

In our current implementation, we utilize the “minimize” function in the scipy.optimize package to optimize the log likelihood $\mathcal{l}\left( 0,\sigma^{2},\rho\right)$ (from Equation S7.18 in Supplementary Methods S7.4) to estimate $\hat{\sigma}_{0}^{2},\hat{\rho}_{0}$. The optimization was initialized with the method of moments estimates $\hat{\rho}_{\mathrm{MoM}},\hat{\sigma}_{\mathrm{MoM}}^{2}$ from Supplementary Methods S7.3.

The score under the Gaussian model is

$U\left( 0 \right)=\frac{\partial}{\partial\mu}\mathcal{l}\left( \mu,\sigma^{2},\rho\right)\left. \right|_{\mu=0}=\left( \frac{\partial\mathcal{l}}{\partial\left( \boldsymbol{D}-\mu\boldsymbol{1} \right)} \right)^{\top}\cdot\frac{\partial\left( \boldsymbol{D}-\mu\boldsymbol{1} \right)}{\partial\mu}\left. \right|_{\mu=0}$ (S7.23)

$=-\frac{1}{2}\left( 2\boldsymbol{\Sigma}^{-1}\left( \boldsymbol{D}-\mu\boldsymbol{1} \right) \right)^{\top}\cdot\left( -\boldsymbol{1} \right)\left. \right|_{\mu=0}$ (S7.24)

$=\left( \boldsymbol{D}-\mu\boldsymbol{1} \right)^{\top}\boldsymbol{\Sigma}^{-1}\boldsymbol{1}\left. \right|_{\mu=0}=\boldsymbol{D}^{\top}\boldsymbol{\Sigma}^{-1}\boldsymbol{1}$ (S7.25)

Equation S7.23 follows from chain rule and Equation S7.24 follows from matrix calculus identity $\frac{\partial}{\partial\boldsymbol{x}}\left( \boldsymbol{x}^{\top}\boldsymbol{\Sigma}^{-1}\boldsymbol{x} \right)=\left( \boldsymbol{\Sigma}^{-1}+{\boldsymbol{\Sigma}^{-1}}^{\top} \right)\boldsymbol{x}=2\boldsymbol{\Sigma}^{-1}\boldsymbol{x}$, where $\boldsymbol{x}=\boldsymbol{D}-\mu\boldsymbol{1}$. The Fisher information under the null hypothesis is

$I\left( 0 \right)=-\frac{\partial^{2}}{\partial^{2}\mu}\mathcal{l}\left( \mu,\sigma^{2},\rho\right)\left. \right|_{\mu=0} =-\frac{\partial}{\partial\mu}\left( \frac{\partial}{\partial\mu}\mathcal{l}\left( \mu,\sigma^{2},\rho\right) \right)\left. \right|_{\mu=0}$ (S7.26)

$=-\frac{\partial}{\partial\mu}\left( \boldsymbol{D}-\mu\boldsymbol{1} \right)^{T}\boldsymbol{\Sigma}^{-1}\boldsymbol{1}\left. \right|_{\mu=0}=\mathbf{1}^{\top}\boldsymbol{\Sigma}^{-1}\mathbf{1}$ (S7.27)

The ratio of the squared score to the Fisher’s information (again, both evaluated under the null hypothesis) converges in distribution to a chi-squared distribution with one degree of freedom (Rao, 1948). Equivalently, the standardized score $U(0)/\sqrt{I\left( 0 \right)}$ converges in distribution to a standard normal.

Let ${\hat{\boldsymbol{\Sigma}}}_{0}=\boldsymbol{\Sigma}\left( \hat{\sigma}_{0}^{2},\hat{\rho}_{0} \right)$. The score test statistic is therefore

$Z_{\text{score}}=\frac{\boldsymbol{D}^{\top}{{\hat{\boldsymbol{\Sigma}}}_{0}}^{-1}\boldsymbol{1}}{\sqrt{\boldsymbol{1}^{\top}{{\hat{\boldsymbol{\Sigma}}}_{0}}^{-1}\boldsymbol{1}}}=\frac{\bar{D}}{\sqrt{\hat{\sigma}_{0}^{2}\left( \frac{1}{2J}+\frac{J-1}{J}\hat{\rho}_{0} \right)}}$ (S7.28)

which is asymptotically standard Gaussian under the null hypothesis. The two-sided p-value is given by $p=2\left( 1-\Phi\left( \left| Z_{\mathrm{score}} \right| \right) \right)$, where $\Phi\left( \cdot\right)$ denote the standard normal cumulative distribution function.

### Likelihood ratio test (LRT) and signed-root transformation

As a final statistical test, we compare the maximum log likelihood under the null hypothesis with the maximum unrestricted log likelihood:

$X_{\text{LRT}}^{2}=-2 \left[ \mathcal{l}\left( 0,\tilde{\sigma}_{0}^{2},\tilde{\rho}_{0} \right)\mathcal{-l}\left( \tilde{\mu},\tilde{\sigma}^{2},\tilde{\rho} \right) \right]$ (S7.29)

where $\left( \tilde{\sigma}_{0}^{2},\tilde{\rho}_{0} \right)$ maximizes the log likelihood assuming the null is true (same as $\left( \hat{\sigma}_{0}^{2},\hat{\rho}_{0} \right)$from the score test in Supplementary Methods S7.6), and $\left( \tilde{\mu},\tilde{\sigma}^{2},\tilde{\rho} \right)$ maximizes the unrestricted log likelihood (same as $\left( \bar{D},\hat{\sigma}_{\mathrm{ML}}^{2},\hat{\rho}_{\mathrm{ML}} \right)$ from the maximum likelihood test in Supplementary Methods S7.4).

Under the null hypothesis, $X_{\mathrm{LRT}}^{2}$ follows the chi square distribution with one degree of freedom. The test statistic is converted to a signed-root form for interpretability:

$Z_{\text{LRT}}=\text{sign}\left( \tilde{\mu} \right)\sqrt{X_{\text{LRT}}^{2}} .$ (S7.30)

The two-sided p-value is given by $p=2\left( 1-\Phi\left( \left| Z_{\mathrm{LRT}} \right| \right) \right)$, where $\Phi\left( \cdot\right)$ denotes the standard normal cumulative distribution function.

### SHARP tests toy example selection

To select among the variants of SHARP, we sampled $\boldsymbol{D}\sim N\left( \mu\mathbf{1},\boldsymbol{\Sigma}(\sigma^{2}, \rho) \right)$ with $\mu=0,\sigma^{2}=1$ while varying $\rho$ from 0.01 to 0.49. *J* = 300, so ***D*** is a vector of length 600. The whole procedure was repeated independently 100 times to compute a FPR for each value of $\rho$. The score test showed the best control of FPR (Fig. S11) and was therefore used for the rest of the manuscript.

### Split-HAlf (SHA) test

As a variant of the SHARP test, we can also randomly divide the dataset into two disjoint halves A and B once (as opposed to *J* times like the SHARP test). For each machine learning model, we then perform K-fold cross-validation in subsets A and B separately. The data split for the K-fold cross-validation is identical for both models, so there is fold-level correspondence between the two models. Denote the fold-level model performance differences $D_{Ak}$ and $D_{Bk}$ respectively, for $k=1,\ldots, K$. In this version of the procedure, there is no replication. This results in two length-$K$ vectors $\boldsymbol{D}_{A}=\left[ D_{A1},\ldots,D_{AK} \right]^{\top}$ and $\boldsymbol{D}_{B}=\left[ D_{B1},\ldots,D_{BK} \right]^{\top}$.

Let $\boldsymbol{D}=\left[ \boldsymbol{D}_{A}^{\top},\boldsymbol{D}_{B}^{\top} \right]^{\top}=\left[ D_{A1},\ldots,D_{AK}, D_{B1},\ldots,D_{BK} \right]^{\top}$, so $\boldsymbol{D}$ is a column vector of length $2K$. We denote $\mathrm{Var}\left( D_{Ak} \right)=Var\left( D_{Bk} \right)=\sigma^{2}$. Because of the disjoint subsets A and B, $\mathrm{Corr}\left( D_{Ak},D_{Bk} \right)=0 but also$for any pair of folds $k$ *&* $k^{'}$, $\mathrm{Corr}\left( D_{Ak},D_{Bk^{'}} \right)=0$. On the other hand, within subsets, $\mathrm{Corr}\left( D_{Ak},D_{Ak^{'}} \right)=Corr\left( D_{Bk},D_{Bk^{'}} \right)=\rho$. Therefore, the covariance matrix of $\boldsymbol{D}$ can be written as

$\boldsymbol{\Sigma}\left( \sigma^{2},\rho\right)=\sigma^{2}\left[ \begin{matrix} \boldsymbol{M} & \boldsymbol{0} \\ \boldsymbol{0} & \boldsymbol{M} \end{matrix} \right]$ (S7.31)

where $\boldsymbol{M}\in\mathbb{R}^{K\times K}$ has ones on the diagonal and *ρ* in all off-diagonal entries,

$M_{jk}=\left\{ \begin{aligned} 1,j=k \\ \rho,j\neq k \end{aligned} \right. \mathrm{or} \boldsymbol{M}=\left[ \begin{matrix} 1 & \rho& \rho\\ \rho& \ddots& \rho\\ \rho& \rho& 1 \end{matrix} \right]$ (S7.32)

and $\boldsymbol{0}\in\mathbb{R}^{K\times K}$ is the zeros matrix. Similar to the SHARP test, we can use various approaches to estimate $\sigma^{2}$ and $\rho$, followed by a Wald test. A likelihood ratio test or score test can also be utilized. The SHA test likely has worse statistical power than the SHARP test, so we did not consider the SHA test in the current study.

## How fold dependence invalidates the permutation test

There are two different permutation tests used in the literature, but both are invalid. The first permutation test is discussed in Supplementary Results. Here we discuss the second permutation test.

Given a vector $\boldsymbol{D}$ of $J$ performance differences between two models, let $\bar{D}$ be the mean of $\boldsymbol{D}$. To generate a null distribution, for each iteration, the sign of each entry of $\boldsymbol{D}$ is flipped with probability 0.5, thus creating a new vector $\boldsymbol{D}_{u}$. The mean of $\boldsymbol{D}_{u}$ is then computed, which we denote as $\bar{D}_{u}$. By repeating the procedure $U$ times, we obtain $U$ null values. The final two-sided p-value is computed as

$p=\frac{1}{U}\sum_{u=1}^{U} \mathbb{I(}\left| \bar{D}_{u} \right|\geq\left| \bar{D} \right|)$ (S8.1)

where $\mathbb{I(}\left| \bar{D}_{u} \right|\geq\left| \bar{D} \right|)$ is an indicator variable that is equal to one if $\left| \bar{D}_{u} \right|\geq\left| \bar{D} \right|$, and zero otherwise. Note that to avoid a p-value of 0, the permutation test p-value is typically computed by adding one to the numerator and denominator of Equation S8.1, i.e., $p=\frac{1}{U+1}\left( 1+\sum_{u=1}^{U} \mathbb{I(}\left| \bar{D}_{u} \right|\geq\left| \bar{D} \right|) \right)$. However, for the purpose of the analysis below, we will use Equation S8.1.

This permutation test wrongly assumes the elements of $\boldsymbol{D}$ are independent. To show this more explicitly, we first note that based on Supplementary Methods S3, we can write:

$\text{E}\left( \bar{D} \right)=\mu$ (S8.2)

$\mathrm{Var}\left( \bar{D} \right)=\sigma^{2}\left( \frac{1}{J}+\frac{J-1}{J}\rho\right)$ (S8.3)

For the null permutation distribution to be valid, we note that under the null hypothesis, we require the following conditions to be true:

$\text{E}\left( \bar{D}_{u} \right)=0$ (S8.4)

$\mathrm{Var}\left( \bar{D}_{u} \right)=\sigma^{2}\left( \frac{1}{J}+\frac{J-1}{J}\rho\right)$ (S8.5)

To evaluate if the above conditions are met, let $D_{j}$ be the *j*-th element of $\boldsymbol{D}$. Then we can write

$\bar{D}_{u}=\frac{1}{J}\sum_{j=1}^{J} s_{j}D_{j}$ (S8.6)

where each $s_{j}$ independently takes values from $\{+1 ,-1\}$ with probability $\frac{1}{2}$. Therefore,

$\text{E}\left( \bar{D}_{u} \right)=\frac{1}{J}\sum_{j=1}^{J} \text{E}\left( s_{j} \right)\text{E}\left( D_{j} \right)$ $=\frac{1}{J}\sum_{j=1}^{J} 0\times\mu=0$ (S8.7)

$\mathrm{Var}\left( \bar{D}_{u} \right)=\frac{1}{J^{2}}\text{E}\left( \text{Var}\left( \sum_{j=1}^{J} s_{j}D_{j} | D_{j} \right) \right)+$ $\frac{1}{J^{2}}\text{Var}\left( \text{E}\left( \sum_{j=1}^{J} s_{j}D_{j} | D_{j} \right) \right)$ $=\frac{1}{J^{2}}\text{E}\left( \sum_{j=1}^{J} D_{j}^{2} \right)+\frac{1}{J^{2}}\text{Var}\left( \text{0} \right)$ $=\frac{1}{J^{2}}\text{E}\left( \boldsymbol{D}^{\top}\boldsymbol{D} \right)$ $=\frac{1}{J^{2}}\left( \text{tr}\left( \boldsymbol{\Sigma} \right)+\mu^{2}J \right)$ $=\frac{\sigma^{2}+\mu^{2}}{J}$, (S8.8)

where we have used the following result: for a given random vector $\boldsymbol{x}$ with mean $\boldsymbol{\mu}$ and covariance $\boldsymbol{\Sigma}$, $\text{E}\left( \boldsymbol{x}^{\top}\boldsymbol{x} \right)=\text{tr}\left( \boldsymbol{\Sigma} \right)+\boldsymbol{\mu}^{\top}\boldsymbol{\mu}$.

Under the null hypothesis $\mu=0$,

$\mathrm{Var}\left( \bar{D}_{u} \right)=\sigma^{2}\frac{1}{J}\leq\sigma^{2}\left( \frac{1}{J}+\frac{J-1}{J}\rho\right)=Var\left( \bar{D} \right)$ (S8.9)

Therefore, the first condition above (Equation S8.4) is met, but the second condition (Equation S8.5) is not met. In particular, since we expect the elements of $\boldsymbol{D}$ to be positively correlated, then $\mathrm{Var}\left( \bar{D}_{u} \right)<\mathrm{Var}\left( \bar{D} \right)$, suggesting that the null distribution is overly narrow. Therefore, the permutation is expected to have an elevated FPR, which we observe empirically (Fig. S5).

## Incorrectly using the corrected resampled paired t-test leads to high FPR

In repeated K-fold cross-validation (CV), the dataset is partitioned into *K* folds, and this procedure is repeated *R* times using different random splits. This yields a vector ***D*** of *J* (= *K×R*) fold-level differences. As described in Methods Section 4.5.5, the corrected resampled paired t-test was proposed to account for fold dependence. The corrected paired t-test utilizes the following statistic:

$T=\frac{\bar{D}}{\sqrt{\left( \frac{1}{J}+\frac{N_{2}}{N_{1}} \right)S^{2}}} ,$ (S9.1)

where $\bar{D}$ is the average of the vector $\boldsymbol{D}$. $N_{1}$​ and $N_{2}$ are the number of training and test samples respectively in a particular split of the dataset into training and test sets. The corrected paired t-test assumes that the correlation between folds is equal to the fraction of the full dataset used in the test fold. For example, if 10-fold cross-validation was repeated 30 times, then $\boldsymbol{D}$ is a vector of length 300, so $J$ = 300 and $N_{2}/N_{1}$=1/9. In this setup, $\rho=1/10$ (implicitly). As shown in Fig. 6, the corrected t-test has slightly elevated FPR.

However, we have observed certain studies implementing an incorrect version of the corrected resampled t-test. For example, if 10-fold cross-validation was repeated 30 times, then instead of creating a vector ***D*** (of length 300), some studies took the matrix of fold-level differences of size 10×30, and averaged each column of the matrix, so they ended up with a vector $\boldsymbol{D}^{*}$ (of length 30). They then utilized the above T statistic (Equation S9.1) using the mean of vector $\boldsymbol{D}^{*}$ in the numerator (which is actually the same as $\bar{D}$) with *J* = 30 and $N_{2}/N_{1}$=1/9.

In other words, the authors implicitly assumed the entries in $\boldsymbol{D}^{\boldsymbol{*}}$ were correlated with $\rho=1/10$. However, the entries in $\boldsymbol{D}^{\boldsymbol{*}}$ (vector of length 30) were likely to be much more correlated than the entries in $\boldsymbol{D}$ (vector of length 300). As such, we expected this incorrect version of the corrected t-test to have significantly higher FPR than the correctly-implemented corrected t-test, which was confirmed in Fig. S5.

# Supplemental Results

## Meta-analysis

In our meta-analysis, we encountered a study using a permutation test that was invalid for reasons unrelated to fold dependence. In this study, the test statistic was the performance difference between models evaluated using 10-fold cross-validation. The labels were then permuted 1000 times. For each permutation, both models were re-evaluated using the same 10-fold cross-validation procedure, thereby constructing a null distribution of performance differences. The p-value was obtained by comparing the observed performance difference to this permutation-based null distribution. However, this procedure did not actually test the null hypothesis of equal predictive performance between models. By permuting labels, the relationship between features and outcomes was destroyed, so the resulting null distribution reflected a no-signal scenario rather than the case where two models performed equally well. We note that this is not an infrequent mistake in the literature. However, since the invalidity reason was not due to fold dependence, we excluded this study from our meta-analysis, so we ended up with 210 studies.

In Fig. 2b, we reported that six studies utilized statistical tests that accounted for fold dependence. Four of them used the corrected resampled t-test (Nadeau and Bengio, 2003), while one of them used the 5×2 t-test (Dietterich, 1998). The final study utilized the following heuristic: a model was declared superior to another model only if the lower bound of its reported performance range (mean − 2 × SD across the 10 cross-validation folds) exceeded the other model’s mean performance. Because the variability term used was the standard deviation rather than the standard error (SD/$\sqrt{10}$), this criterion can be seen as a variant of the empirical test of differences, which implicitly accounts for fold dependence (Supplementary Methods S5). As such, we consider it to accommodate fold dependency by avoiding the independence-based $\sqrt{10}$ shrinkage of variability.

In Fig. 2c, we categorized 15 studies that employed statistical tests ignoring fold dependence under “Others.” Of these, eight studies used ANOVA (Fisher, 1992), a generalization of the resampled paired t-test for comparing more than two models. Two studies applied the Nemenyi test (Nemenyi, 1963), and one study each employed the Quade test (Quade, 1979), the correlated coefficient z-test (Meng et al., 1992), the Kolmogorov-Smirnov test (Massey, 1951), the U-test (Harrell, 2001) and the Tukey-Kramer test (Kramer, 1956).

# Supplemental Tables

Table S1. Criteria for evaluating journal statistical rigor & methodological guidance

| Category | Score 0 | Score 1 | Score 2 |
| --- | --- | --- | --- |
| Code availability policy | No mention in guidelines/policies; no encouragement or requirement to share code/algorithms. | Code sharing encouraged or optional but not required for publication. | Code sharing is mandatory and a condition for publication. |
| Data availability policy | No mention in guidelines/policies; no encouragement or requirement to share data. | Data sharing (any type) encouraged or optional but not required. | Data sharing is mandatory and a condition for publication. |
| Code for peer review policy | No mention of providing code specifically for peer review. | Code submission for review encouraged but not mandatory. | Code submission required for peer review. |
| Availability statement requirement | No request or mention of a data/code/materials availability statement. | Availability statement encouraged but not mandatory. | Formal availability statement required for submission/publication. |
| Statistical test guidance rigor (11 items*) | None of the 11 statistical reporting items mentioned. | 1–3 of the 11 items mentioned. | More than 3 of the 11 items mentioned. |
| Statistical review policy | No mention or requirement of statistical review in reviewer guidance or peer review policy. | Statistical review acknowledged and optional. | Statistical review required for specified article types (e.g., clinical trials, ML, meta-analysis, high-dimensional data). |
| Reporting standards/checklists | No scientific reporting checklist mentioned; no requirement or encouragement of external reporting guidelines. | Reporting checklists encouraged but not required. | Completion of one or more scientific reporting checklists required for submission, review, or acceptance. |

*Statistical Test Guidance Rigor – 11 Items Considered:

1. Report test name; 2. Report p-value with ns/s; 3. Report sample size per group;

4. State one- or two-tailed; 5. Justify test/assumption checks; 6. State alpha level;

7. Report degrees of freedom; 8. Carry out multiple testing correction;

9. Include clinical/practical significance; 10. Report confidence intervals;

11. Explain methods for constructing confidence intervals.

# Supplemental Figures

DeLong test


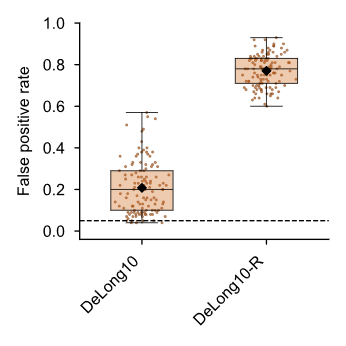


**Figure S1. False positive rate (FPR) for DeLong’s test with 10-fold cross-validation repeated once (“DeLong10”) or repeated 30 times (“DeLong10-R”).** The DeLong’s test is only applicable to binary classification, so the current analyses were based on the Covertype dataset. Each boxplot comprised 120 datapoints, corresponding to 120 scenarios (4 sample sizes × 3 hyperparameter schemes × 10 noise levels). The black dot indicates the mean FPR across 120 scenarios.

FPR across datasets


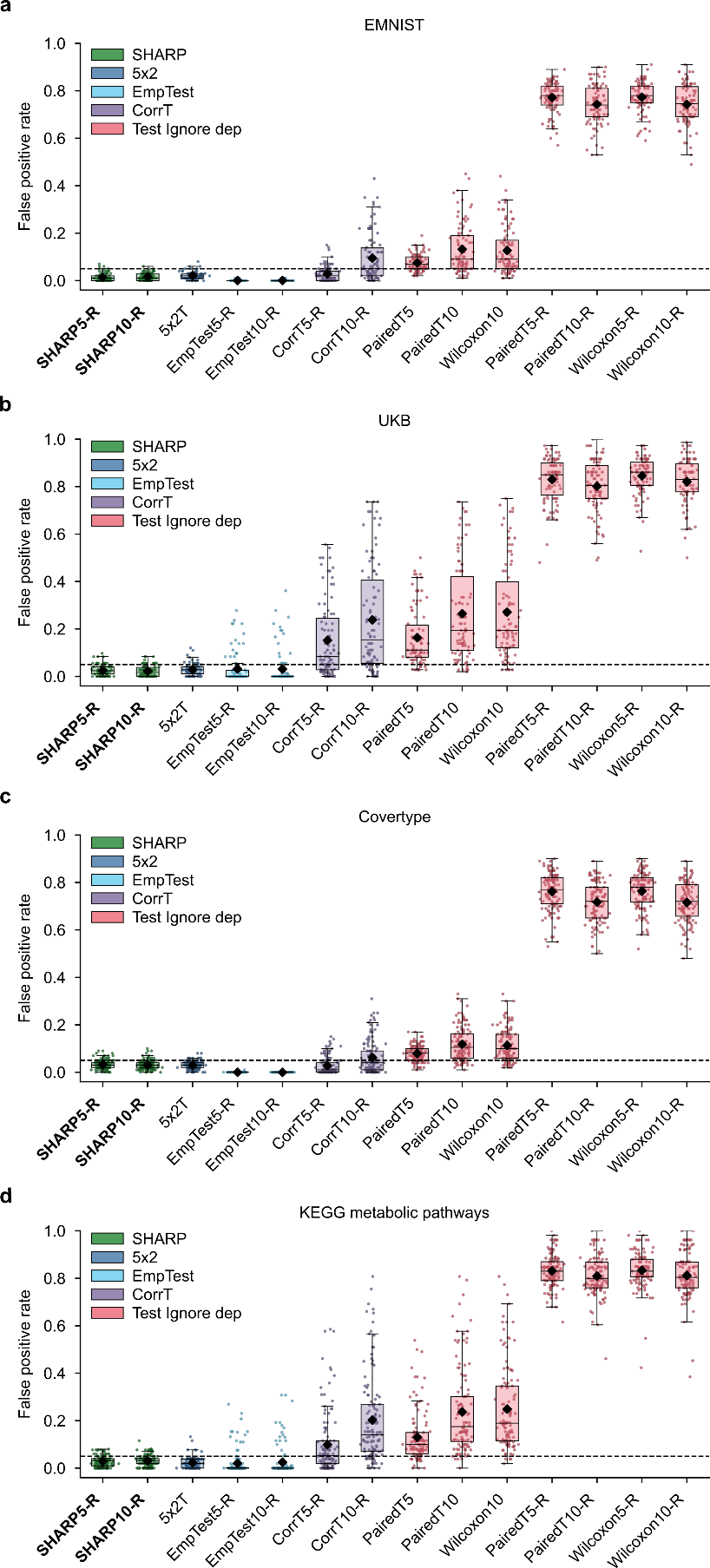


**Figure S2. False positive rates (FPR) of various statistical tests across 420 scenarios broken down by datasets. a.** Boxplots of FPRs in the EMNIST dataset. Each boxplot comprises 90 data points, corresponding to 90 scenarios (3 sample sizes × 10 noise levels × 3 hyperparameter schemes). **b.** Boxplots of FPRs in the UKB dataset. Each boxplot comprises 90 datapoints, corresponding to 90 scenarios (3 sample sizes × 10 noise levels × 3 hyperparameter schemes). **c.** Boxplots of FPRs in the Covertype dataset. Each boxplot comprises 120 datapoints, corresponding to 120 scenarios (4 sample sizes × 10 noise levels × 3 hyperparameter schemes). **d.** Boxplots of FPRs in the metabolic dataset. Each boxplot comprises 120 datapoints, corresponding to 120 scenarios (4 sample sizes × 10 noise levels × 3 hyperparameter schemes). **Test naming conventions.** Numeric suffixes "5" and "10" denote 5-fold and 10-fold cross-validation. The "-R" suffix indicates repeated cross-validation: 5-fold repeated 60 times or 10-fold repeated 30 times, yielding 5 × 60 = 10 × 30 = 300 fold-level statistics. For example, "CorrT5-R" denotes the corrected resampled t-test evaluated under 60 repetitions of 5-fold cross-validation, while "PairedT10" denotes the naïve paired t-test under a single run of 10-fold cross-validation. "SHARP5-R" denotes the split-half procedure repeated 60 times with 5-fold cross-validation within each half; "SHARP10-R" denotes 30 repetitions with 10-fold cross-validation within each half. The number of repetitions was chosen such that the FPR of tests accounting for fold dependence had stabilized; tests that ignore fold dependence do not stabilize, with FPR increasing toward 1 as repetitions grow (Fig. 5d). "CorrT" denotes the corrected resampled t-test (Nadeau & Bengio, 2003). “EmpTest” denotes the empirical test of differences (Parkes et al., 2021). For details on all tests, see Methods Sections 4.5 and 4.6.

FPR across sample sizes


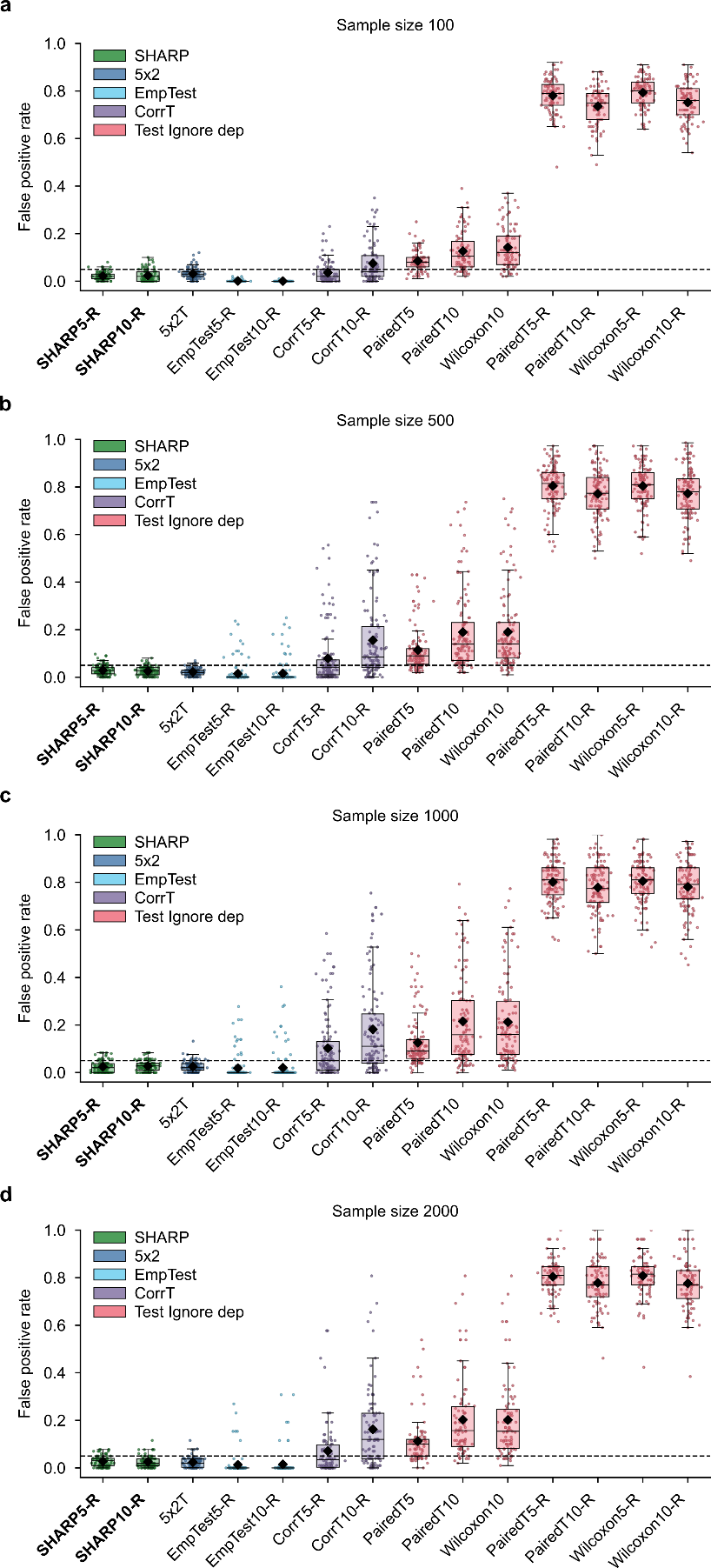


**Figure S3. False positive rates (FPRs) of various statistical tests across 420 scenarios broken down by sample sizes. a.** Boxplots of FPRs for simulations involving sample size of 100. Each boxplot comprises 90 datapoints, corresponding to 90 scenarios (3 datasets × 10 noise levels × 3 hyperparameter schemes). **b.** Boxplots of FPRs for simulations involving sample size of 500. Each boxplot comprises 120 datapoints, corresponding to 120 scenarios (4 datasets × 10 noise levels × 3 hyperparameter schemes). **c.** Boxplots of FPRs for simulations involving sample size of 1000. Each boxplot comprises 120 datapoints, corresponding to 120 scenarios (4 datasets × 10 noise levels × 3 hyperparameter schemes). **d.** Boxplots of FPRs for simulations involving sample size of 2000. Each boxplot comprises 90 datapoints, corresponding to 90 scenarios (3 datasets × 10 noise levels × 3 hyperparameter schemes). **Test naming conventions.** Numeric suffixes "5" and "10" denote 5-fold and 10-fold cross-validation. The "-R" suffix indicates repeated cross-validation: 5-fold repeated 60 times or 10-fold repeated 30 times, yielding 5 × 60 = 10 × 30 = 300 fold-level statistics. For example, "CorrT5-R" denotes the corrected resampled t-test evaluated under 60 repetitions of 5-fold cross-validation, while "PairedT10" denotes the naïve paired t-test under a single run of 10-fold cross-validation. "SHARP5-R" denotes the split-half procedure repeated 60 times with 5-fold cross-validation within each half; "SHARP10-R" denotes 30 repetitions with 10-fold cross-validation within each half. The number of repetitions was chosen such that the FPR of tests accounting for fold dependence had stabilized; tests that ignore fold dependence do not stabilize, with FPR increasing toward 1 as repetitions grow (Fig. 5d). "CorrT" denotes the corrected resampled t-test (Nadeau & Bengio, 2003). “EmpTest” denotes the empirical test of differences (Parkes et al., 2021). For details on all tests, see Methods Sections 4.5 and 4.6.

FPR for two-algorithm scheme


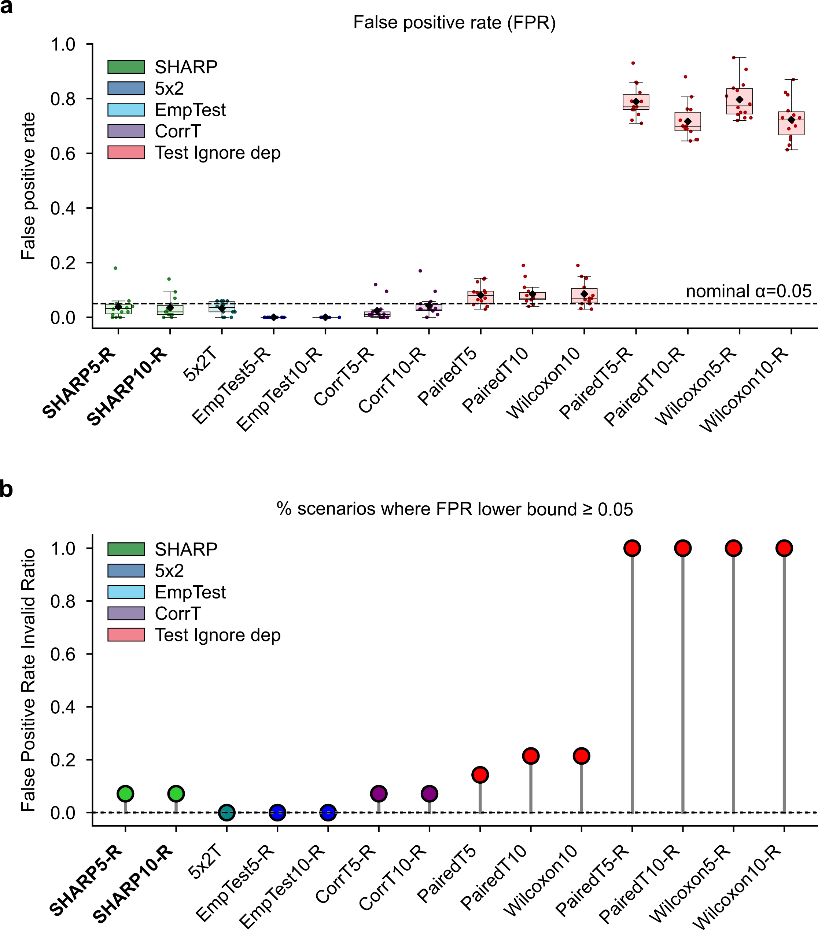


**Figure S4. False positive rates (FPRs) with the two-algorithm simulation scheme (Supplementary Methods S6). a.** Boxplots of FPR for each statistical test. Each boxplot contains 14 values, corresponding to the 14 scenarios. The black dot indicates the mean FPR across 14 scenarios. The dashed horizontal line marks the nominal FPR of 0.05. **b.** FPR inflation rate: the percentage of the 14 scenarios in which the lower bound of the 95% confidence interval for FPR exceeded 0.05, indicating inadequate control of the Type I error rate. **Test naming conventions.** Numeric suffixes "5" and "10" denote 5-fold and 10-fold cross-validation. The "-R" suffix indicates repeated cross-validation: 5-fold repeated 60 times or 10-fold repeated 30 times, yielding 5 × 60 = 10 × 30 = 300 fold-level statistics. For example, "CorrT5-R" denotes the corrected resampled t-test evaluated under 60 repetitions of 5-fold cross-validation, while "PairedT10" denotes the naïve paired t-test under a single run of 10-fold cross-validation. "SHARP5-R" denotes the split-half procedure repeated 60 times with 5-fold cross-validation within each half; "SHARP10-R" denotes 30 repetitions with 10-fold cross-validation within each half. The number of repetitions was chosen such that the FPR of tests accounting for fold dependence had stabilized; tests that ignore fold dependence do not stabilize, with FPR increasing toward 1 as repetitions grow (Fig. 5d). "CorrT" denotes the corrected resampled t-test (Nadeau & Bengio, 2003). “EmpTest” denotes the empirical test of differences (Parkes et al., 2021). For details on all tests, see Methods Sections 4.5 and 4.6. Conclusions were the same as Fig. 6, except that the corrected resampled t-test reliably controlled FPR in this simulation.

FPR for more tests


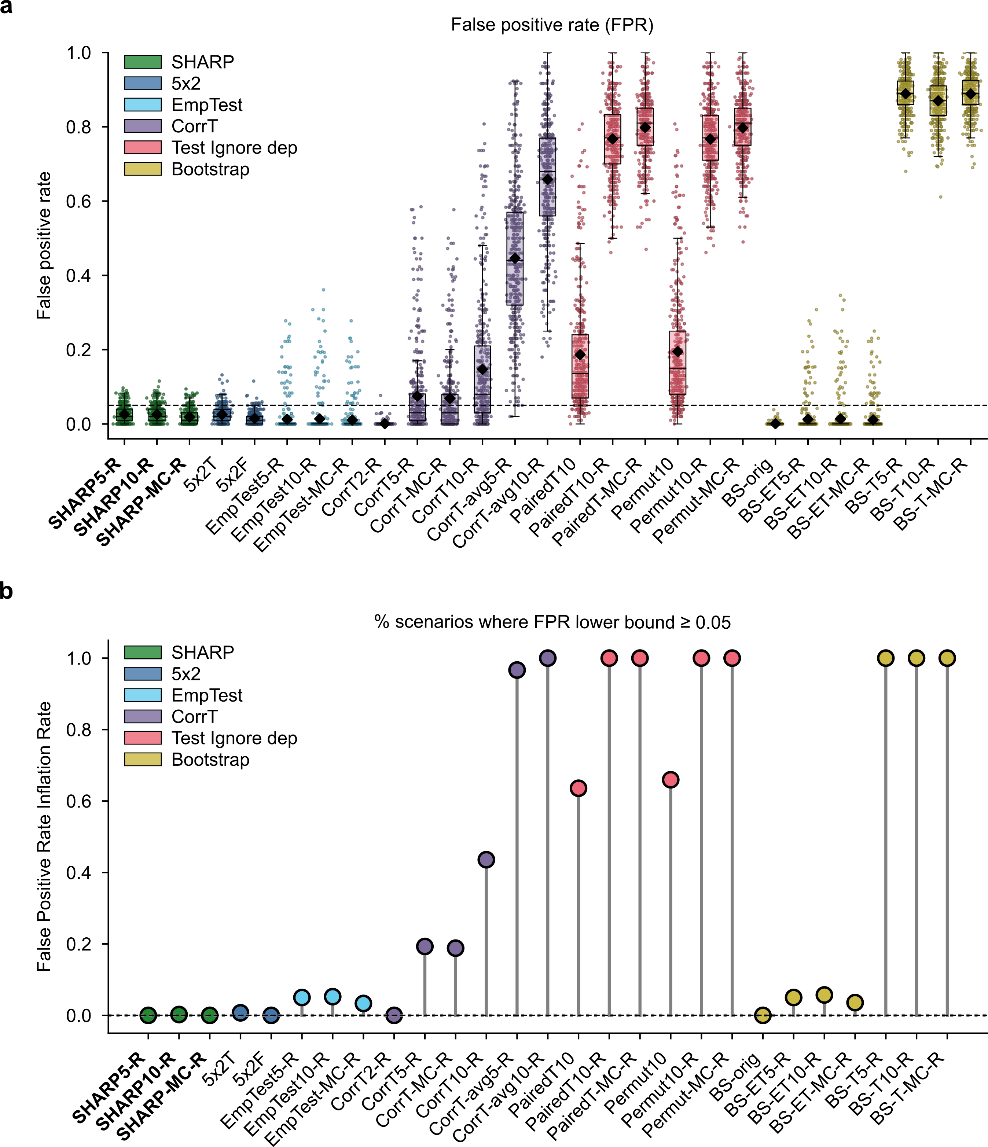


**Figure S5. False positive rate (FPR) across 420 simulation scenarios for an extended set of tests. a.** Boxplot of FPRs for each statistical test, with one value per scenario (n = 420). Black dots mark the mean FPR across scenarios. The dashed horizontal line marks the nominal level of 0.05. **b.** FPR inflation rate: the percentage of the 420 scenarios in which the lower bound of the 95% confidence interval for FPR exceeded 0.05, indicating inadequate control of the Type I error rate. **Test naming conventions.** Numeric suffixes "5" and "10" denote 5-fold and 10-fold cross-validation. The "-R" suffix indicates repeated cross-validation, with the number of repetitions chosen such that the FPR of tests accounting for fold dependence had stabilized. Under standard cross-validation, "-R" corresponds to 5-fold repeated 60 times or 10-fold repeated 30 times, yielding 5 × 60 = 10 × 30 = 300 fold-level statistics. For example, "EmpTest5-R" denotes the empirical test of differences under 60 repetitions of 5-fold cross-validation, while "PairedT10" denotes the naïve paired t-test under a single run of 10-fold cross-validation. For SHARP variants, each repetition produces one pair of statistics — one per half — so the number of pairs equals the number of repetitions: "SHARP5-R" denotes the split-half procedure repeated 60 times with 5-fold cross-validation within each half (yielding 60 pairs), and "SHARP10-R" denotes 30 repetitions with 10-fold cross-validation within each half (yielding 30 pairs). The "MC" suffix denotes Monte Carlo cross-validation, where the dataset is randomly split into 80% training and 20% test sets. Under Monte Carlo cross-validation, "-R" corresponds to 300 repetitions, again yielding 300 fold-level statistics; for "SHARP-MC-R", a single Monte Carlo split is performed within each half and the split-half procedure is repeated 300 times (yielding 300 pairs). "CorrT" denotes the corrected resampled t-test (Nadeau & Bengio, 2003); "CorrT-avg" denotes a misapplied variant (Supplementary Methods S9). "EmpTest" denotes the empirical test of differences (Parkes et al., 2021). "Permut" denotes the paired permutation test (Methods Section 4.5.3). "BS" denotes bootstrap with three variants (Methods Section 4.5.8): bootstrap-orig (“BS-orig”), bootstrap-empirical-test-of-differences (“BS-ET”) and bootstrap-t-test (“BS-T”). Two bootstrap variants (“BS-orig” and “BS-ET”) reliably controlled FPR, but the third bootstrap variant (“BS-T”) exhibited high FPR. For details about the various tests, see Methods Sections 4.5 and 4.6.

Power Simulation Scheme (Noisy-Model)


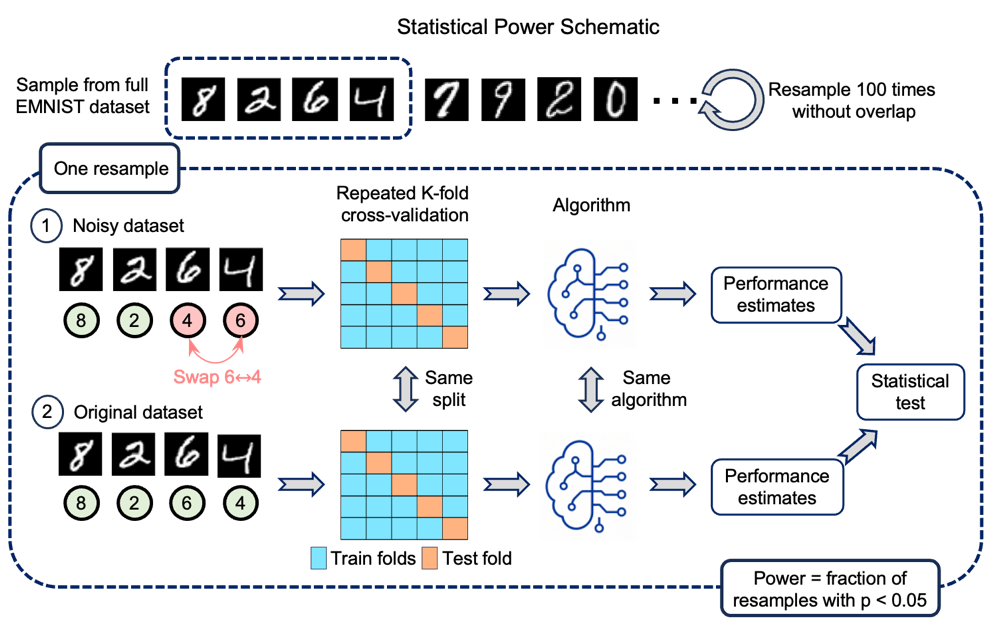


**Figure S6. Statistical power simulation scheme illustrated for EMNIST.** We randomly sampled a dataset (e.g., N = 1,000) from a full dataset, then generated a noisy version by permuting the target labels (ground-truth digits) for a fixed percentage of samples (e.g., 10%). For example, the noisy dataset (above) has the labels for digits 6 and 4 swapped. Using identical cross-validation splits and the same machine learning algorithm on the clean and noisy datasets, we obtained pairs of cross-validated performance estimates — e.g., 300 pairs for 10-fold cross-validation repeated 30 times. A statistical test was applied to the paired vector to obtain a p-value. Because only one dataset was corrupted, the model trained on the clean data had higher expected predictive performance by construction, so a sensitive test should reject the null hypothesis. This procedure was repeated up to 100 times using non-overlapping subsamples, with power computed as the percentage of repetitions in which the null was rejected. In total, there are 420 scenarios.

Power for more tests


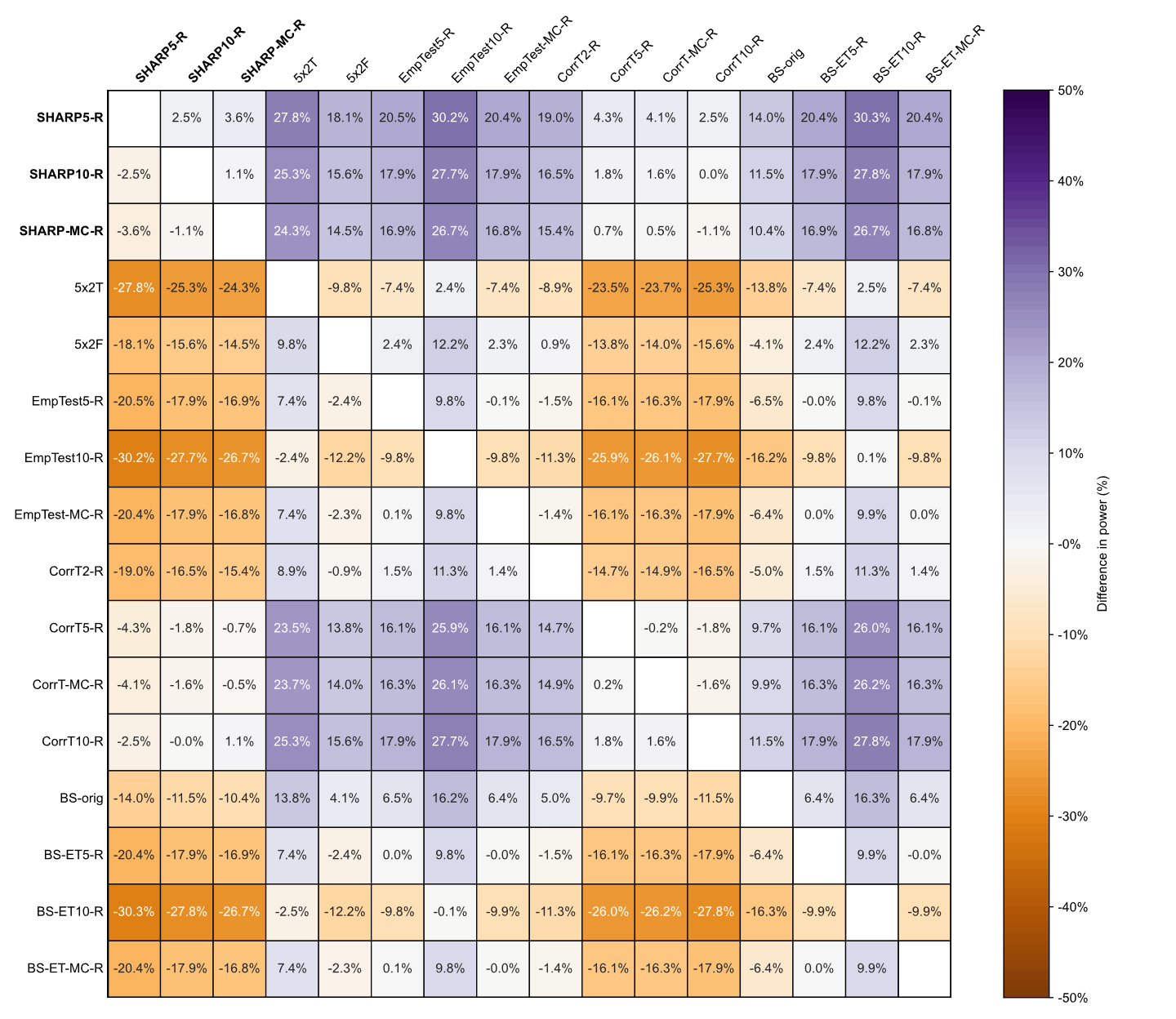


**Figure S7. Comparison of statistical power across 420 scenarios for an extended set of tests.** Difference in power between pairs of statistical tests, computed as the power of the statistical test (on the row) minus the power of the statistical test (on the column), averaged across 420 scenarios. A purple cell indicates that the test on the row achieved higher power than the test on the column; an orange cell indicates the opposite. The set of statistical tests was the same as Fig. S5, except that only valid statistical tests are included. The SHARP test with repeated 5-fold cross-validation (SHARP5-R) demonstrated the best power, as indicated by the entirely purple SHARP5-R row. **Test naming conventions.** Numeric suffixes "5" and "10" denote 5-fold and 10-fold cross-validation. The "-R" suffix indicates repeated cross-validation, with the number of repetitions chosen such that the power of tests accounting for fold dependence had stabilized. Under standard cross-validation, "-R" corresponds to 5-fold repeated 60 times or 10-fold repeated 30 times, yielding 5 × 60 = 10 × 30 = 300 fold-level statistics. For example, "EmpTest5-R" denotes the empirical test of differences under 60 repetitions of 5-fold cross-validation. For SHARP variants, each repetition produces one pair of statistics — one per half — so the number of pairs equals the number of repetitions: "SHARP5-R" denotes the split-half procedure repeated 60 times with 5-fold cross-validation within each half (yielding 60 pairs), and "SHARP10-R" denotes 30 repetitions with 10-fold cross-validation within each half (yielding 30 pairs). The "MC" suffix denotes Monte Carlo cross-validation, where the dataset is randomly split into 80% training and 20% test sets. Under Monte Carlo cross-validation, "-R" corresponds to 300 repetitions, again yielding 300 fold-level statistics; for "SHARP-MC-R", a single Monte Carlo split is performed within each half and the split-half procedure is repeated 300 times (yielding 300 pairs). "CorrT" denotes the corrected resampled t-test (Nadeau & Bengio, 2003); "EmpTest" denotes the empirical test of differences (Parkes et al., 2021). "BS" denotes bootstrap with two variants (Methods Section 4.5.8): bootstrap-orig ("BS-orig") and bootstrap-empirical-test-of-differences ("BS-ET"). For details about the various tests, see Methods Sections 4.5 and 4.6.

Power for two-algorithm scheme


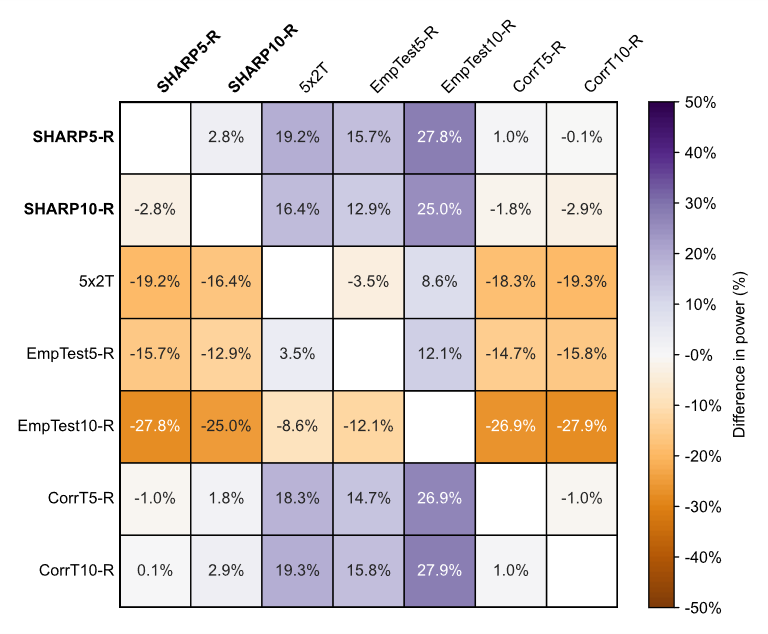


**Figure S8. Comparison of statistical power of statistical tests with the two-algorithm simulation scheme (Supplementary Methods S6).** Difference in power between pairs of statistical tests, computed as the power of the statistical test (on the row) minus the power of the statistical test (on the column), averaged across 14 scenarios. A purple cell indicates that the test on the row achieved higher power than the test on the column; an orange cell indicates the opposite. The set of statistical tests was the same as Fig. 7a. The SHARP test with repeated 5-fold cross-validation (SHARP5-R) and the corrected resampled t-test with repeated 10-fold cross-validation (CorrT10-R) had the best power among the tests that achieved nominal FPR control.

CI coverage for more tests


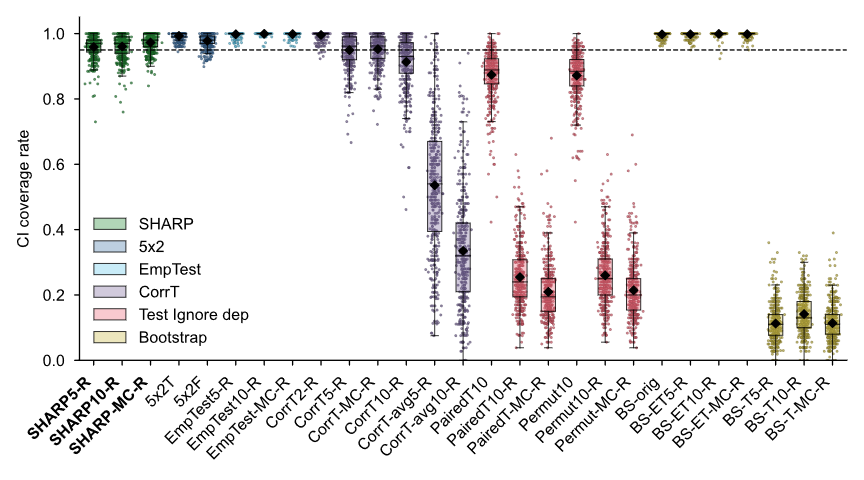


**Figure S9. Comparison of 95% confidence interval (CI) coverage rate across 420 scenarios for an extended set of tests.** The CI coverage rate of a scenario is defined as the percentage of non-overlapping subsamples in which the (estimated) true performance difference between models fell inside the 95% CI of a given test. For a well-calibrated test, the coverage rate should be exactly 95% (black dashed line). Each boxplot comprises 420 data points, each representing the CI coverage rate of one scenario. The black dot indicates the mean coverage rate across 420 scenarios. Tests that ignored fold dependence (red boxplots) had overly narrow CIs, so their coverage rates were much lower than 95%. **Test naming conventions.** Numeric suffixes "5" and "10" denote 5-fold and 10-fold cross-validation. The "-R" suffix indicates repeated cross-validation, with the number of repetitions chosen such that the FPR of tests accounting for fold dependence had stabilized. Under standard cross-validation, "-R" corresponds to 5-fold repeated 60 times or 10-fold repeated 30 times, yielding 5 × 60 = 10 × 30 = 300 fold-level statistics. For example, "EmpTest5-R" denotes the empirical test of differences under 60 repetitions of 5-fold cross-validation, while "PairedT10" denotes the naïve paired t-test under a single run of 10-fold cross-validation. For SHARP variants, each repetition produces one pair of statistics — one per half — so the number of pairs equals the number of repetitions: "SHARP5-R" denotes the split-half procedure repeated 60 times with 5-fold cross-validation within each half (yielding 60 pairs), and "SHARP10-R" denotes 30 repetitions with 10-fold cross-validation within each half (yielding 30 pairs). The "MC" suffix denotes Monte Carlo cross-validation, where the dataset is randomly split into 80% training and 20% test sets. Under Monte Carlo cross-validation, "-R" corresponds to 300 repetitions, again yielding 300 fold-level statistics; for "SHARP-MC-R", a single Monte Carlo split is performed within each half and the split-half procedure is repeated 300 times (yielding 300 pairs). "CorrT" denotes the corrected resampled t-test (Nadeau & Bengio, 2003); "CorrT-avg" denotes a misapplied variant (Supplementary Methods S9). "EmpTest" denotes the empirical test of differences (Parkes et al., 2021). "Permut" denotes the paired permutation test (Methods Section 4.5.3). "BS" denotes bootstrap with three variants (Methods Section 4.5.8): bootstrap-orig (“BS-orig”), bootstrap-empirical-test-of-differences (“BS-ET”) and bootstrap-t-test (“BS-T”). Two bootstrap variants (“BS-orig” and “BS-ET”) reliably controlled FPR, but the third bootstrap variant (“BS-T”) exhibited high FPR. For details about the various tests, see Methods Sections 4.5 and 4.6.

CI coverage (two-algorithm)


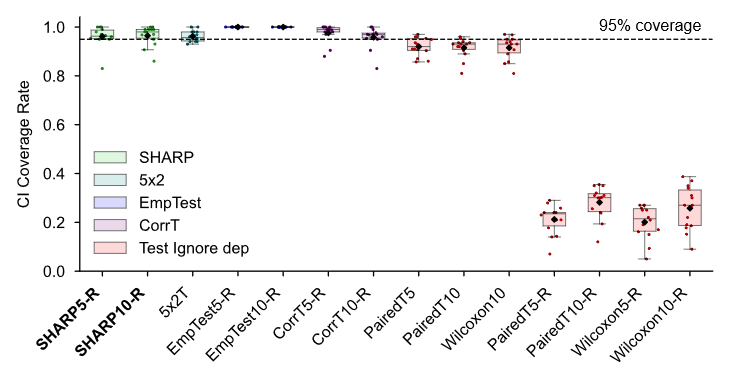


**Figure S10. Comparison of 95% confidence interval (CI) coverage rate across statistical tests under the two-algorithm simulation scheme (Supplementary Methods S6)**. The CI coverage rate of a scenario is defined as the percentage of non-overlapping subsamples in which the (estimated) true performance difference between models fell inside the 95% CI of a given test. For a well-calibrated test, the coverage rate should be exactly 95% (black dashed line). Each boxplot comprises 14 data points, each representing the CI coverage rate of one scenario. The black dot indicates the mean coverage rate across 14 scenarios. Tests that ignored fold dependence (red boxplots) had overly narrow CIs, so their coverage rates were much lower than 95%. The test naming conventions are the same as Fig. S4.

Toy simulation for SHARP test


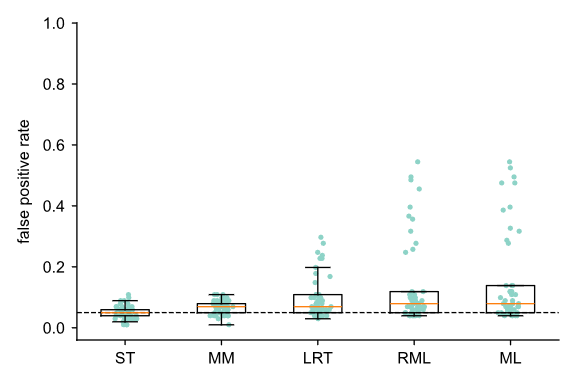


**Figure S11. Toy simulation to select the best variant of the SHARP test**. We randomly draw performance differences of two methods from a Gaussian distribution, $\boldsymbol{D} \sim N\left( \mu\mathbf{1},\boldsymbol{\Sigma}(\sigma^{2}, \rho) \right)$with $\mu=0, \sigma=1$, while $\rho$ varies from 0.01 to 0.49. There are in total 49 dots in each box corresponding to the 49 values of $\rho$. Score test shows the best false positive control. Test abbreviations: ST: Score Test; MM: Method of Moments; LRT: Likelihood Ratio Test; RML: Restricted Maximum Likelihood Test; ML: Maximum Likelihood Test. The score test performed the best, so was used throughout the manuscript.

# References

Ali, M. (2020). *PyCaret: An open source, low-code machine learning library in Python*. https://www.pycaret.org

Alpaydin, E. (1999). Combined 5\times 2 cv F test for comparing supervised classification learning algorithms. *Neural Computation*, *11*(8), 1885–1892.

Bouckaert, R. R., & Frank, E. (2004). Evaluating the Replicability of Significance Tests for Comparing Learning Algorithms. In H. Dai, R. Srikant, & C. Zhang (Eds.), *Advances in Knowledge Discovery and Data Mining* (Vol. 3056, pp. 3–12). Springer Berlin Heidelberg. https://doi.org/10.1007/978-3-540-24775-3_3

Dietterich, T. G. (1998). Approximate statistical tests for comparing supervised classification learning algorithms. *Neural Computation*, *10*(7), 1895–1923.

Fisher, R. A. (1992). Statistical Methods for Research Workers. In S. Kotz & N. L. Johnson (Eds.), *Breakthroughs in Statistics* (pp. 66–70). Springer New York. https://doi.org/10.1007/978-1-4612-4380-9_6

Harrell, F. E. (2001). *Regression Modeling Strategies: With Applications to Linear Models, Logistic Regression, and Survival Analysis*. Springer New York. https://doi.org/10.1007/978-1-4757-3462-1

Hogg, R. V., McKean, J. W., & Craig, A. T. (2013). *Introduction to mathematical statistics*. Pearson Education India.

Kramer, C. Y. (1956). Extension of multiple range tests to group means with unequal numbers of replications. *Biometrics*, *12*(3), 307–310.

Lehmann, E. L., & Casella, G. (1998). Minimaxity and Admissibility. In *Theory of Point Estimation* (pp. 309–427). Springer-Verlag. https://doi.org/10.1007/0-387-22728-8_5

Massey, F. J. (1951). The Kolmogorov-Smirnov Test for Goodness of Fit. *Journal of the American Statistical Association*, *46*(253), 68–78. https://doi.org/10.1080/01621459.1951.10500769

Meng, X.-L., Rosenthal, R., & Rubin, D. B. (1992). Comparing correlated correlation coefficients. *Psychological Bulletin*, *111*(1), 172.

Nadeau, C., & Bengio, Y. (2003). Inference for the Generalization Error. *Machine Learning*, *52*(3), 239–281. https://doi.org/10.1023/A:1024068626366

Nemenyi, P. B. (1963). *Distribution-free multiple comparisons.* Princeton University. https://search.proquest.com/openview/c1f3e8829e8351e9c2a1c5e51778c6cf/1?pq-origsite=gscholar&cbl=18750&diss=y

Newcombe, R. G. (1998). Two-sided confidence intervals for the single proportion: Comparison of seven methods. *Statistics in Medicine*, *17*(8), 857–872. https://doi.org/10.1002/(SICI)1097-0258(19980430)17:8%253C857::AID-SIM777%253E3.0.CO;2-E

Parkes, L., Moore, T. M., Calkins, M. E., Cieslak, M., Roalf, D. R., Wolf, D. H., Gur, R. C., Gur, R. E., Satterthwaite, T. D., & Bassett, D. S. (2021). Network controllability in transmodal cortex predicts positive psychosis spectrum symptoms. *Biological Psychiatry*, *90*(6), 409–418.

Pedregosa, F., Varoquaux, G., Gramfort, A., Michel, V., Thirion, B., Grisel, O., Blondel, M., Prettenhofer, P., Weiss, R., & Dubourg, V. (2011). Scikit-learn: Machine learning in Python. *The Journal of Machine Learning Research*, *12*, 2825–2830.

Quade, D. (1979). Using Weighted Rankings in the Analysis of Complete Blocks with Additive Block Effects. *Journal of the American Statistical Association*, *74*(367), 680–683. https://doi.org/10.1080/01621459.1979.10481670

Rao, C. R. (1948). Large sample tests of statistical hypotheses concerning several parameters with applications to problems of estimation. *Mathematical Proceedings of the Cambridge Philosophical Society*, *44*(1), 50–57. https://www.cambridge.org/core/journals/mathematical-proceedings-of-the-cambridge-philosophical-society/article/large-sample-tests-of-statistical-hypotheses-concerning-several-parameters-with-applications-to-problems-of-estimation/B83FAA6838A7E7D933EA3582C784ED06

Yan, X. (2009). *Linear regression analysis: Theory and computing*. world scientific. https://books.google.com/books?hl=en&lr=&id=MjNv6rGv8NIC&oi=fnd&pg=PR5&dq=Linear+Regression+Analysis:+Theory+and+Computing&ots=30-bdkOi2Q&sig=An6uEYyc0T5y37EIGJm8fJhWwS4
